# Fouling of Reverse Osmosis (RO) and Nanofiltration (NF) Membranes by Low Molecular Weight Organic Compounds (LMWOCs), Part 1: Fundamentals and Mechanism

**DOI:** 10.3390/membranes14100221

**Published:** 2024-10-17

**Authors:** Yasushi Maeda

**Affiliations:** LG Chem Japan Co., Ltd., Kyobashi Trust Tower 12F, 2-1-3 Kyobashi Chuo-ku, Tokyo 104-0031, Japan; jpmaeda@lgchem.com; Tel.: +81-3-5299-4530

**Keywords:** fouling, organic fouling, marginal water, internal fouling, surface fouling, flow loss, reverse osmosis, nanofiltration, thin-film composite, polyamide, piperazine, NOM, EfOM, surfactant, phonolics, plasticizer, oil and grease, leachables, antiplasticization, octanol-water partition coefficient, pore size, network pore, aggregate pore, free volume, troubleshooting, autopsy

## Abstract

Reverse osmosis (RO) and nanofiltration (NF) are ubiquitous technologies in modern water treatment, finding applications across various sectors. However, the availability of high-quality water suitable for RO/NF feed is diminishing due to droughts caused by global warming, increasing demand, and water pollution. As concerns grow over the depletion of precious freshwater resources, a global movement is gaining momentum to utilize previously overlooked or challenging water sources, collectively known as “marginal water”. Fouling is a serious concern when treating marginal water. In RO/NF, biofouling, organic and colloidal fouling, and scaling are particularly problematic. Of these, organic fouling, along with biofouling, has been considered difficult to manage. The major organic foulants studied are natural organic matter (NOM) for surface water and groundwater and effluent organic matter (EfOM) for municipal wastewater reuse. Polymeric substances such as sodium alginate, humic acid, and proteins have been used as model substances of EfOM. Fouling by low molecular weight organic compounds (LMWOCs) such as surfactants, phenolics, and plasticizers is known, but there have been few comprehensive reports. This review aims to shed light on fouling behavior by LMWOCs and its mechanism. LMWOC foulants reported so far are summarized, and the role of LMWOCs is also outlined for other polymeric membranes, e.g., UF, gas separation membranes, etc. Regarding the mechanism of fouling, it is explained that the fouling is caused by the strong interaction between LMWOC and the membrane, which causes the water permeation to be hindered by LMWOCs adsorbed on the membrane surface (surface fouling) and sorbed inside the membrane pores (internal fouling). Adsorption amounts and flow loss caused by the LMWOC fouling were well correlated with the octanol-water partition coefficient (log P). In part 2, countermeasures to solve this problem and applications using the LMWOCs will be outlined.

## 1. Introduction

Reverse osmosis (RO) is a liquid-phase pressure-driven separation process in which applied transmembrane pressure causes selective movement of solvent against its osmotic pressure difference [1]. Since RO is the tightest membrane, almost all impurities can be removed except dissolved gasses and low molecular weight substances such as methanol, urea, boron, etc. Thus, RO is mainly used to desalinate seawater/brackish water and concentrate valuable substances. Nanofiltration (NF) is also classified as a pressure-driven membrane process with a wide range of performance characteristics between RO and ultrafiltration (UF) [2]. NF membranes offer high rejection of salts of divalent anions as well as organics having a molecular weight above 200. NF has attracted the attention of municipalities due to its broad spectrum of separation capability. It involves natural organic matter (NOM) and color removal, disinfection by-product (DBP) precursor removal, membrane softening (MS) for hardness removal, and synthetic organic compound (SOC) removal [3].

RO was initially used for specialized or high-value applications, including seawater desalination, municipal water treatment, ultra-pure water (UPW) production, etc. However, as the technology matured and costs decreased, RO applications broadened to include industrial process waters and wastewater reuse areas. This expansion was facilitated by the development of advanced RO elements such as low fouling membranes and thicker feed spacer. Similarly, the NF technique is now used in various wastewater treatments together with hybrid processes [2,4].

Along with extending RO/NF applications to marginal water resources [5,6], fouling has been a critical issue. It is said that marginal waters with high fouling tendencies, like effluents or contaminated surface or well waters, cause significant fouling problems for membrane plants [7]. More than 30 years ago, Paul and Rahman asked themselves whether membrane fouling was the final frontier in RO [8]. While RO fouling may not actually be the “final” frontier, they mentioned that it was clearly a frontier, an incompletely known territory with many uncharted areas.

Despite significant advancements in RO/NF technologies, fouling remains a persistent and unresolved issue or challenge in RO operations and continues to be a frontier in the field. An increased number of published technical articles might support this observation. Ibrahim et al. [9] and Matin et al. [10] reported the number of publications on RO fouling. Publication numbers have appreciably increased after 2000 and have risen exponentially in the last 3–4 years. To better understand specific issues on RO/NF fouling, it is beneficial to examine the numerous review articles and their subject matter. Many review articles were published during the last few years [11,12,13,14,15,16,17,18,19,20], including on specific topics such as scaling [21,22,23], organic [24], and biofouling [21,25,26,27,28,29,30,31,32,33].

One of the typical symptoms of fouling is the observation of appreciable product flow decline or feed pressure increase during operation. Among those, RO/NF plants occasionally encounter unexplained rapid flow decline problems at the beginning of the operation, including phenomena of compaction and setting for unrelaxed membranes. An increase in rejection often accompanies this. The point different from typical fouling is that even if the membrane surface is analyzed (visual observation) by autopsy, no significant fouling substances can be seen [34,35]. According to the author’s experience, these phenomena are often attributed to the following causes:

1. Membrane oxidation by halogens;
2. Organic fouling by low molecular weight organic compounds (LMWOCs) (sorption of LMWOCs to RO/NF membranes).

Firstly, as oxidation deterioration progresses, a decrease in rejection and an increase in the flow rate are usually observed for halogen oxidation. However, at the beginning of oxidation, the permeate flow rate is decreased under some conditions, such as acidic pH, halogen species, etc. For example, Larson et al. [36] reported that 0.5 ppm of residual chlorine induced an immediate flux drop at pH 5.6. This flow loss is especially noticeable with bromine and iodine. It is reported that bromine and iodine cause flow decline at neutral and acidic conditions [37]. Therefore, a membrane manufacturer advises not to use iodine germicides. In this halogen oxidation, halogen can be detected by electron spectroscopy for chemical analysis (ESCA).

While the first case is not often encountered, the following organic fouling cases are frequently experienced, especially in wastewater treatment. The fundamental research during developing thin-film composite (TFC) RO membranes found that nonionic and cationic surfactants, quaternary germicides, and phenolic compounds cause flux loss [36,38]. It is reasonable to consider that the cationic substances cause flux decline due to electrostatic interaction since TFC RO membranes are typically negatively charged at a neutral pH. Surprisingly, neutral organic substances also induce heavy flow loss. Darton et al. [35] mention that the worst foulant for the membrane service company is the foulant that cannot be seen and sampled. Foulants such as cationic flocculants and other organic contaminants fall into this category, bonding to the membrane surface in a monomolecular layer, which is hard to identify and even harder to remove.

LMWOCs that cause flow reduction are not limited to nonionic surfactants and phenols. Exploring a wide variety of LMWOCs is the main subject of this review. This paper reviews the LMWOCs that cause RO/NF membrane fouling, their mechanisms, and countermeasures. Furthermore, some applications utilizing LMWOCs (i.e., rejection enhancement and rejuvenation) are also introduced in Part 2.

## 2. Fouling: Definition and Problems in Membrane Processes

Membrane separation processes can be categorized according to the applied driving forces. Macedonio and Drioli [39] listed the following four driving forces: (1) hydrostatic pressure, (2) concentration gradient or chemical potential-driven processes, (3) electrical potential, and (4) temperature. Among them, pressure-driven membranes play an important role in liquid separations. Four major pressure-driven liquid separation membrane processes can be divided by the pore sizes of membranes and the required transmembrane pressure (TMP), as shown in Table 1. Furthermore, these membrane processes can be classified into low-pressure membrane (LPM) systems (MF and UF) and high-pressure membrane (HPM) systems (NF and RO) depending on TMP [40].

**Figure 1:**
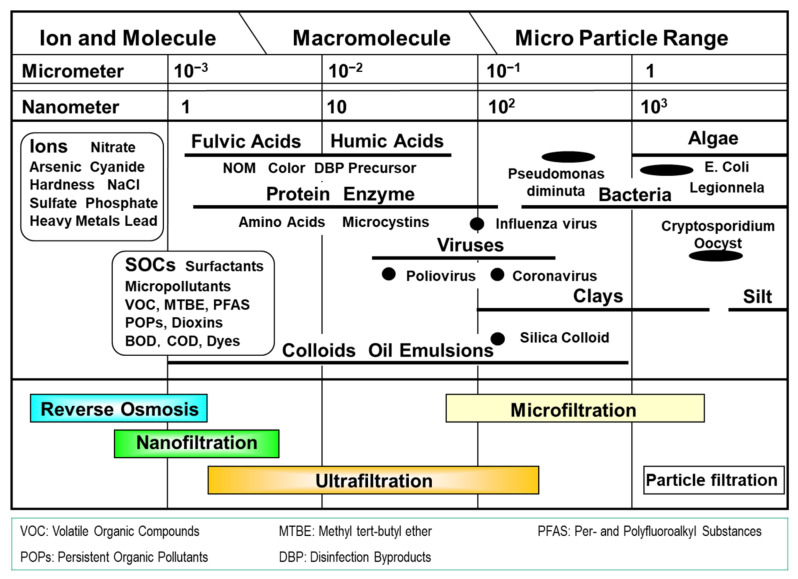
Roles of pressure-driven liquid separation membranes.

The roles of these liquid separation membranes are summarized in Figure 1. MF can remove particles and bacteria greater than 0.1 µm in diameter. UF can remove colloids, viruses, and high-molecular-weight organic compounds [40]. Since LPMs are used for removing turbidity and particles and pretreatment of RO/NF, they are sometimes called clarification membranes. LPMs, such as membrane bioreactor (MBR), have been widely used for solid-liquid separation. Further, removing pathogenic microbes, such as protozoa (cryptosporidium and giardia), has attracted attention in municipal water treatment.

RO and NF are also called desalting membranes since the key role of RO/NF is to remove dissolved ions from brackish and seawater. Therefore, they are commonly used for desalination and removal of heavy metal ions. NF is classified as an intermediate membrane process between RO and UF, with molecular weight cut-off (MWCO) ranging from 200 to 1000 and NaCl rejection of less than 90%. NF can be used to remove hardness (membrane softening), disinfection by-product (DBP) precursors, pesticides, etc. [2].

Although these membrane processes have been utilized in a variety of applications, fouling remains one of the significant challenges in membrane operation [42,43,44,45]. Furthermore, fouling problems became more severe and complicated when extending their applications to difficult waters such as industrial and municipal wastewater, oily wastewater (produced water and oil emulsion), etc. [46,47,48,49,50]. Lipnizki [51] mentioned that fouling is and will continue to be the Achilles’ heel of membrane filtration. So far, the term fouling has been used without clearly defining its phenomena and mechanisms. This section briefly explains the definition of fouling for pressure-driven membranes, foulant classifications, and fouling mechanisms. 

### 2.1. Definition of General Membrane Fouling

The definition of membrane fouling has been described in many publications. Some articles clearly and concisely explain the definition of membrane fouling [1,45,52,53,54,55,56]. Among them, two definitions are shown below:

- Irreversible deposition of retained particles, colloids, macromolecules, salts, etc., at the membrane surface or inside the membrane at the pore wall, which causes a continuous flux decline [52,53].
- A process resulting in the loss of performance of a membrane due to the deposition of suspended or dissolved substances on its external surfaces, at its pore openings, or within its pores [1].

In addition, Tay and Song [54] explain the time course of fouling. They introduce a traditional concept of membrane fouling that is directly defined phenomenologically as “flux decline with time”. In terms of the effect of fouling, Zainol Abidin et al. [45] mention that fouling leads not only to a reduction in the flux and lifespan of the membrane that is coupled with an increase in the differential pressure and feed pressure but also a reduction in the treated water quality, rise in the energy consumption and operation cost, and eventual deterrent of the widespread application of membrane technology. Thus, these definitions might be categorized into five key components as shown in Table 2.

Fouling locations are sometimes categorized into two cases: inside the membrane pores (internal fouling) and on the membrane surface (surface or external fouling) [57,58,59]. External fouling occurs on the top surface of the membrane due to the accumulation of bacteria, extracellular polymeric substances (EPS), macromolecules, or large particles that do not enter the pores. In contrast, internal fouling occurs within the internal pore structure of the membrane due to the deposition and adsorption of colloids, small particles, and proteins or other macromolecules that can pass into the pores [60].

### 2.2. Mechanism of Membrane Fouling

**Figure 2:**
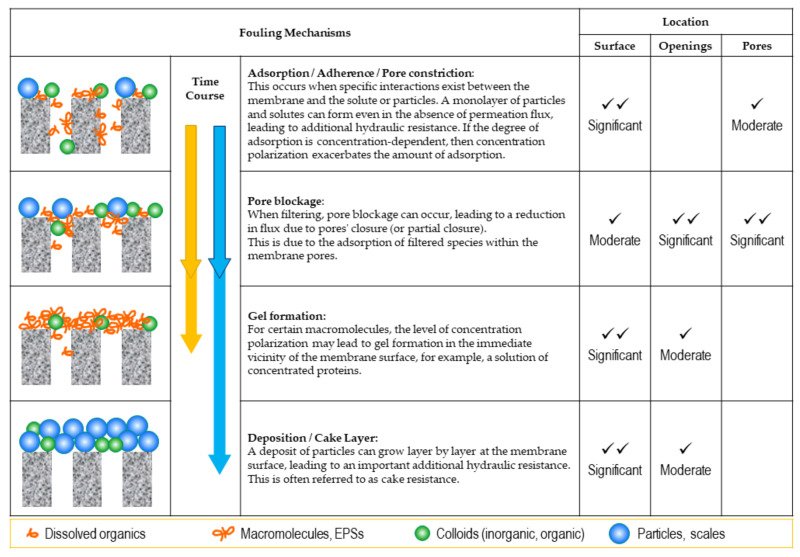
Fouling mechanisms of pressure-driven liquid separation membranes [53,61,62]. The yellow arrow indicates the gel formation process, and the blue arrow indicates the process leading to the cake layer formation.

Mulder [53] explained the fouling phenomena in four forms: adsorption, pore blocking, precipitation, and cake formation. Similarly, Field [61] mentioned that fouling may take the following forms: adsorption, pore blocking, deposition, and gel formation. Zainol et al. [45] explain slightly different mechanisms: standard blocking, complete blocking, intermediate blocking, and cake formation. Based on Field’s explanations about locations and time course, the four fouling mechanisms are schematically shown in Figure 2.

Song [63] showed a typical flux change behavior occurring in fouling:

1. Starting with a rapid initial drop compared with that of pure water filtration;
2. A long-term gradual flux decrease;
3. Reaching to a steady-state flux.

These three stages can be explained as follows:

The rapid initial drop from the flux of pure water filtration is attributed to the quick blocking of membrane pores. The maximum flux (the flux of pure water filtration) always occurs at the beginning of filtration because all membrane pores are available at that moment. Flux declines as the retained particles block membrane pores. The pores are more likely to be blocked partially, and the degree of pore blockage depends on the shape and the relative size of the particles and the pores. The blockage will generally be more complete when the particles and pores are similar in both shape and size [1,21,23].

Further flux decline after pore blockage is due to the formation and growth of the cake layer on the membrane surface. A cake layer is formed on the membrane surface as the retained particles increase. The cake layer creates additional resistance to the permeate flow, and the resistance of the cake layer increases with the growth of the cake layer’s thickness. Consequently, the permeate flux continues to decrease with time.

### 2.3. Typical Membrane Foulants

Foulant types are significantly different depending on membrane processes (LPM, HPM, MBR, etc.), membrane/module configurations (hollow fiber, tubular, plate and frame, spiral wound, etc.), membrane materials, and applications (drinking water, food and beverage, wastewater, seawater desalination, etc.). However, they are roughly classified into the following four categories: (1) particulates/colloids, (2) organics, (3) inorganics, and (4) micro-biological organisms [42,61,64]. Some examples of these foulants are shown in Table 3.

Scott [67] also introduced several examples of foulants in membrane separation. Among them, he listed smaller molecules, such as polypropylene glycol, which is used as an antifoam agent during fermentation, as examples of organic foulants.

## 3. RO/NF Fouling: Definition and Foulant Classification

Belfort [68] (1977) mentioned that membrane fouling is a common cause of poor performance in RO (hyperfiltration). Membrane fouling has been widely accepted as the single largest cause of permeate flux decline at normal operating pressures and temperatures in brackish water systems [8]. Despite a lot of efforts to resolve various fouling issues, fouling has remained a critical problem and challenge in RO/NF operations [55,67,69]. These problem statements might be verified by seeing the results of the membrane autopsy. Membrane autopsy is helpful in identifying membrane failures and their causes [70]. In recent years, membrane analysis companies and RO membrane suppliers have frequently performed such autopsy analyses. García et al. [71] mentioned that membrane failures could be attributed to the presence of fouling, chemical damage (oxidation processes), and physical damage. From more than 600 autopsies, they pointed out that in almost 75% of the cases, fouling is the main cause of membrane failures. As for seawater desalination membranes, 63% of cases were reported [72]. Thus, it is safe to say that fouling continues to be the primary failure of RO operations.

This section provides an overview of the definition of RO/NF fouling and the fouling substances (foulants) specific to RO/NF.

### 3.1. Definition of RO/NF Membrane Fouling

The definition of RO/NF fouling is essentially the same as that for general membrane separation, as previously described. However, there are specific considerations for RO/NF, such as fouling locations. Since it is recognized that fouling may occur in the feed channels in hollow fiber and spiral wound configurations [53] or occur between the membrane sheets of a module, where spacers are located to create space for cross-flow stream [70,73], one cannot neglect the feed channels as a fouling location. Given that the spiral wound type is currently the most commonly used RO/NF module, the following definition might be appropriate: “Fouling is the accumulation of foreign materials from feed water on the active membrane surface and/or on the feed spacer to the point of causing operational problems” [74].

Most review articles agree that the main fouling mechanism for RO membranes is surface fouling since these membranes do not have distinguishable pores and are considered to be essentially non-porous [15,55,73,75]. The idea [56,76] that surface fouling dominates the fouling mechanism for the TFC RO membranes is evident in the works conducted by Lee et al. [77,78]. By utilizing the resistance-in-series model, various hydraulic resistances were elucidated, including the intrinsic membrane resistance (*R_m_*), the cake resistance (*R_c_*), the polarization layer resistance (*R_p_*), the external fouling resistance (*R_ef_*), and the internal fouling resistance (*R_if_*). In a study of surface water (Han River, Korea) pretreated by either MF or UF, it was found that *R_if_* of NF membrane was negligible compared to other resistances, which suggests that membrane fouling due to organic adsorption was less significant than that due to particle deposition and cake formation [77].

Nevertheless, there are reports suggesting that internal fouling plays an appreciable role for specific membranes dependent on feedwater characteristics, e.g., total organic carbon (TOC) and chemical oxygen demand (COD). As for cellulose acetate (CA) RO membranes, Belfort [68] speculates that reversible, shear-sensitive fouling probably occurs on the surface, and irreversible fouling probably occurs inside the membrane skin. The fact that low-rejection, “loose” membranes foul more easily than high-rejection, “tight” membranes supports the presumption of pore blockage [79]. Combe et al. [80] studied the effect of annealing temperature on the amount of humic acid (HA) adsorption. The lower absorption amount was observed for the tight membrane annealed at a high temperature (80 °C). They speculated that HA adsorption occurs both inside the pore and on the membrane surface. Furthermore, Paul and Rahman [8] suggested that organics in the water that dissolve into the membrane (sorption) might change its characteristics. In the case of NF, the sorption of organic molecules into the membrane matrix changes the free volume in the membrane. Depending on the molecule, the interaction can either increase or decrease this free volume and hence flux [81].

Hoek et al. [82] proposed a new definition for internal fouling, describing it as a change in membrane structure due to physical compaction or chemical degradation, which alters solute and solvent transport. Chemical degradation occurs when specific chemicals, such as chlorine, attack the polymer structure. Physical compaction occurs when polymeric membranes are subjected to high hydraulic pressures, a phenomenon well-known in seawater desalination, especially under high temperatures and pressure [83]. Prominent flow decline has been observed in actual seawater desalination plants [84,85]. Flow decline phenomena associated with membrane structure changes have been reported, e.g., membrane oxidation by bromine or iodine, contact with glutaraldehyde, membrane setting for NF or ultra low-pressure (ULP) RO, etc. As mentioned in the Introduction, halogens induce a decrease in permeate flow under specific conditions, such as acidic pH, halogen species, etc. A case study from a Mediterranean seawater desalination plant reported a rapid degradation of RO membrane performance, manifesting as a 40% decrease in the normalized permeate flow. After extensive autopsy analysis, including ESCA, it was speculated that certain forms of iodine in seawater could react with PA and severely affect the membrane performance [86]. Glutaraldehyde, known as an effective biocide, tends to react with residual amines in the PA TFC RO membrane, causing permanent loss of permeate production [87].

Not to be confused with fouling, it is important to note that RO and NF elements may undergo structural changes during their initial use, often referred to as “membrane setting” [88]. Most RO/NF elements are shipped in an unstabilized form or swollen conditions due to residual monomers, additives (humectants and surfactants, etc.), and preservatives. Thus, appreciable initial flow decrease has occasionally been reported, especially for NF and ULP RO.

### 3.2. RO/NF Foulants

Regarding the foulants of RO/NF membranes, the fouling substances explained in the previous section can be applied in general. However, unlike other membrane separation processes, RO/NF can have its own problems with fouling. For example, with MF/UF, biofouling can be reasonably managed because chlorine is used for backwashing, whereas biofouling is a serious problem with PA membranes that are not chlorine-resistant. The shift from CA membranes (pre-1980s) to TFC RO membranes has impacted fouling patterns due to differences in chlorine tolerance. In addition, the current mainstream spiral wound RO elements are susceptible to turbidity, so fouling due to silt components and colloids becomes a problem. Furthermore, as the applications expand, the foulants also change. For example, when treating municipal wastewater, calcium phosphate is often detected from fouled RO elements, and organic fouling has become a critical problem. This section provides an overview of fouling substances that are problematic in RO/NF systems.

As shown in Table 3, RO/NF foulants have also been categorized like the foulant classifications for general membranes. Potts et al. [79] classified RO membrane foulants into three categories: (i) sparingly soluble inorganic compounds such as CaSO_4_, (ii) colloidal or particulate matter, and (iii) dissolved organic carbon (DOC). Biological growth was recognized as a potential foulant. This classification may stem from the fact that the CA membranes primarily used at the time had a certain level of chlorine tolerance and could inhibit biological growth [87,89], while their use in sewage and industrial wastewater treatment was also limited. The continuous application of chlorine (0.5 mg/L chlorine residual) was anticipated to inhibit the growth of most organisms encountered in the Water Factory 21 (WF-21) [90]. However, biofouling has become a serious concern after developing PA RO membranes in hollow fiber and TFC forms due to their low chlorine tolerance and membrane surface characteristics, such as surface charge and morphology [67]. When the PA hollow fiber RO started applying to seawater desalination, biofouling was recognized as a stumbling block in operation due to bacterial aftergrowth in the pretreatment chlorination-dechlorination process [91]. Hence, Flemming et al. [92] mentioned that biofouling represents the Achilles’ heel of the membrane process because all other fouling components, such as organic and inorganic dissolved substances and particles, can be removed by pretreatment.

Thus, the RO/NF foulants are generally classified into the following four groups [13,33,67,93,94]:

- Colloidal fouling [95]: Deposition of colloidal particles on the membrane. The most common types of this colloid are alumino- or iron-silicates (clay or silt). Dissolved silica can precipitate at a concentration below saturation in the presence of metals, including calcium, aluminum, or iron, forming colloidal materials. Several methods or indices have been proposed to predict the colloidal fouling potential of feed waters, including turbidity, silt density index (SDI), and Modified Fouling Index (MFI). The SDI is the most commonly used fouling index [96].
- Organic fouling: Deposition and sorption of macromolecular organic compounds and specific LMWOCs on and/or inside the membrane.
- Inorganic scaling [21,22,23]: Precipitation or crystallization of sparingly dissolved inorganic compounds such as CaCO_3_ and CaSO_4_. Calcium phosphate scaling is a common problem in advanced municipal wastewater treatment. Metals like iron and manganese can be oxidized from soluble to insoluble forms within an RO membrane and precipitate on the membrane [69].
- Biofouling [21,25,26,27,28,29,30,31,32]: Adhesion and accumulation of microorganisms and development of biofilm on the membrane and/or feed spacer. A biofilm is described as bacterial aggregates attached to a surface; the biofilm structure includes a matrix of bacterially produced EPS. The EPS comprises polysaccharides, proteins, and nucleic acids, and has been proven to play a major role in biofouling formation [69]. A typical symptom of biofouling is an increase in the differential pressure, especially at the first stage [97,98].

Examples of these foulants are summarized in Table 4. The organic fouling is separated into macromolecules and LMWOCs. In the case of LMWOC fouling, internal fouling, along with surface adsorption, is considered to play a significant role. This phenomenon is described in subsequent sections.

Correctly identifying the foulant in RO/NF plants is essential in solving operational problems and determining corrective measures and the proper cleaning methods. However, it is not easy to identify the exact foulants from operational data and appearance. Further, in actual RO/NF plants, different types of fouling can occur simultaneously, influencing each other [99]. Membrane autopsy has emerged as a valuable tool for precisely identifying foulants. Here, rather than looking at individual fouling behavior, an overview of the foulant types affected by applications and chronological change is examined. Summarized element analysis data were first reported by Tasaka et al. [100]. They investigated the 82 LP RO elements (NTR-759HR) used for brackish water, high-purity water, and wastewater applications in Japan. The following three were reported as critical foulants for brackish water feed: silica scaling, colloids, and biofouling. Only biofouling and organic fouling were observed for the pure water systems with low TDS. It was speculated that trace amounts of absorbed organics on the membrane surface such as leachables from ion exchange resins caused flux decline.

In foulant analysis, organic fouling is usually detected by loss on ignition (LOI). LOI testing gives an approximation of the organic content of the foulant. LOI greater than 35% indicates the presence of organic matter [101]. Values higher than 60–65% represent notable organic fouling. Fourier transform infrared (FT-IR) spectroscopy analysis can support identifying functional groups associated with organic matter, such as polysaccharides and proteins.

Biofouling in membrane systems can be identified through various methods, with visual inspections serving as an initial step to identify biofilm or slime growth on the feed spacer. Typical biofilms contain over 90% moisture content [102]. For a more comprehensive analysis, adenosine triphosphate (ATP) and inorganic (inductively coupled plasma, ICP) analysis may help to quantify biofouling. Sulfur (S) and phosphorous (P) are commonly associated with biofouling [103]. However, it is still not easy to distinguish between organic fouling and biofouling by simple methods. Thus, in autopsy analysis reports, organic and biofouling are frequently combined. For example, Pietsch et al. [104] reported the summarized element analysis data conducted over three years from 2012 to 2014. The organic foulant portion was organic (31%) and organic/inorganic mix (8%). Here, organics include both biofouling and organic fouling, i.e., bacteria, fungi, yeast, and algae, as well as HA and fulvic acids.

One membrane service company and its preceding companies have conducted a series of element autopsy analyses. The data are summarized in Table 5. In most cases, organic and biofouling are combined. In the 2013 data, fouling with SOCs, such as mineral oil, chlorine compounds, etc., were separately listed. It can be seen that the organic/biofouling portion accounts for about 50% of the total, which has not changed for more than 20 years. Further, combined portions of organic/biological and particulate/colloidal fouling account for 70% of fouling causes.

**Figure 3:**
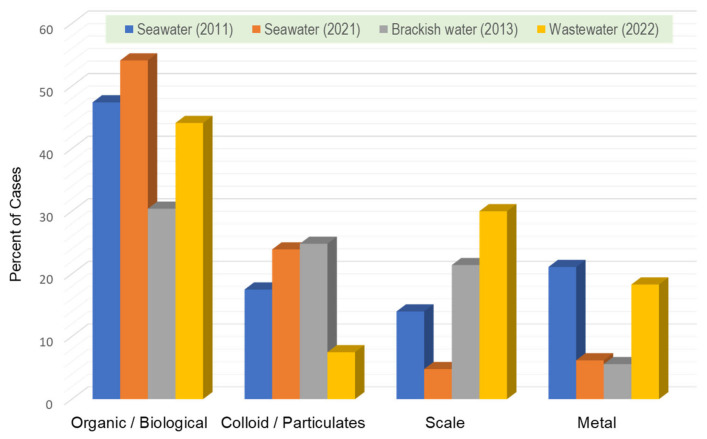
Main foulants detected in seawater, brackish water, and wastewater applications.

To better understand the foulant types in different feed sources and their chronological changes, the extracted data are shown in Figure 3. 

In the case of seawater desalination membranes, the biggest problem is organic/biofouling, which is almost unchanged between 2011 and 2021 data, accounting for about 50%. The difference in the data up to 2021 is that the contribution of scale and metal has decreased significantly. Although there have been advances in pretreatment technology for colloidal fouling over the past decade, including the use of LPMs, it can be seen that fouling caused by colloids and particulates still exists. Biofouling, colloids, and scale are equally problematic in brackish water treatment. In wastewater treatment, the data for MF/UF are also included, so a direct comparison cannot be made. Considering that backwashing with chlorine is routinely performed in MF/UF, the contribution of biofouling in RO/NF is expected to be more significant.

## 4. Organic Fouling with LMWOCs

As mentioned, organic fouling and biofouling are serious concerns in RO operations. Organics in feedwater are known to be the most difficult foulants to remove during pretreatment steps [14]. Therefore, it is not unusual for organic matter to always be present in an RO feed. This section identifies the potential organic foulants in RO/NF feedwater, followed by an explanation of the effects of LMWOCs on TFC RO performance.

### 4.1. Potential Organic Foulants in Various Feedwater Sources and Their Classifications

Organic fouling causes irreversible flux decline due to the adsorption or deposition of dissolved organic matter (DOM) [81]. Organic fouling can be the adsorption at a molecular level or as a monolayer, the formation of a gel on the membrane surface, the deposition or cake formation by organic colloids, or the pore restriction and blocking by molecules that can penetrate the membrane. DOM can be classified into three different categories according to their origins: (1) refractory NOM originated from surface water, colored groundwater, etc., (2) SOCs added by consumers and disinfection by-products (DBPs) generated during disinfection processes of water and wastewater treatment, and (3) soluble microbial products (SMPs) formed during the biological treatment processes due to decomposition of organic compounds [15,94,110]. Algal organic matter (AOM) produced by common bloom-forming algae is harmful in seawater desalination. AOM primarily comprises high molecular weight biopolymers, i.e., polysaccharides and proteins. These types of organics are summarized in Table 6, where typical feed sources to RO/NF are also indicated. SMPs and AOM are categorized into the same family. Organic matter present in biologically treated sewage effluent is referred to as EfOM and consists of NOM, SOC, and SMPs [111,112]. 

NOM in water contains organic compounds that are both hydrophobic and hydrophilic with a wide range of molecular weights [56]. There are several ways to fractionate NOM, such as the XAD (adsorptive resins) method and UF [113]. Liquid chromatography-organic carbon detection (LC-OCD) technology has become popular in characterizing NOM [114]. LC-OCD is a fractionation method based on size exclusion chromatography coupled with different online analyzers such as organic carbon detector (OCD) and UV absorbance at 254 nm. The LC-OCD chromatogram can be divided into five different fractions named: (1) biopolymers that are composed of polysaccharides and proteins, (2) HA (and fulvic acid), (3) building blocks that correspond to breakdown products of humics, (4) low molecular-weight (LMW) organic acids, and (5) LMW neutrals (alcohols, aldehydes, ketones, and amino acids) [115]. 

SOCs are man-made DOMs that are artificially added chemicals or generated during disinfection [15]. A large number of different synthetic organic molecules in rather small quantities are evident in surface water and wastewater, e.g., surfactants, organic priority pollutants, volatile organic compounds, and agricultural pesticides [116]. Amjad [117] raised the following SOCs as harmful foulants of RO/NF: surfactants, detergents, high molecular weight polymeric flocculants (Polydiallyldimethylammonium chloride, polyDADMAC), and polyacrylate-based antiscalants. It was demonstrated that the presence of low levels of polyDADMAC can significantly reduce the performance of cooling water treatment programs. Polyacrylate-based antiscalant can form an insoluble salt with calcium, thus leading to membrane fouling. SOCs have generally more adverse effects on RO/NF membranes compared with NOM [96].

Not all organic substances listed in Table 6 act as foulants for RO/NF membranes, but the ability of organic foulants to foul membranes depends on their affinity for RO membrane, molecular weight, and functionality [55]. For example, lower alcohols and mono- or disaccharides do not show a harmful effect. Furthermore, the negatively charged TFC RO membrane surfaces repulse organic polyelectrolytes with negative functional groups. Meanwhile, because most RO membranes are made of hydrophobic polymers, hydrophobic organic compounds in the feed water are preferentially adsorbed onto the membrane surfaces.

**Figure 4:**
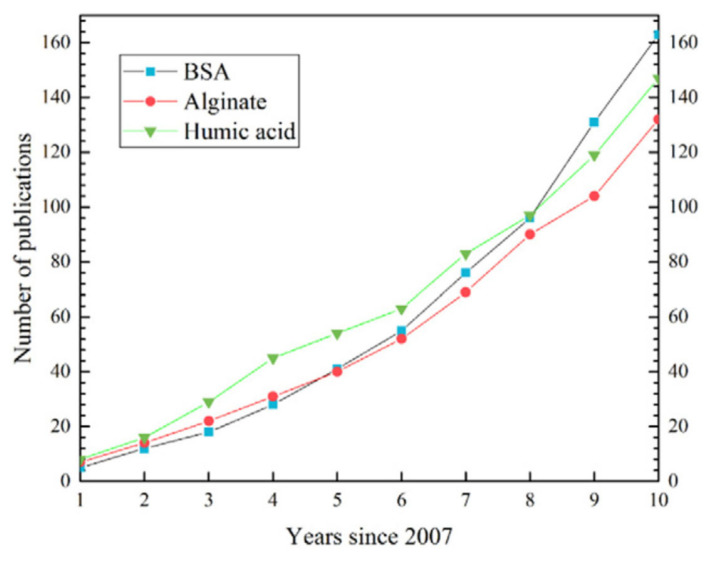
Cumulative number of publications related to three common RO organic foulants studied from 2007 to 2016 [13]. Reprinted with copyright permission.

HA, polysaccharides, and protein fouling have been extensively studied among these organics. Jiang et al. [13] show the cumulative number of publications related to three common RO organic foulants studied during the ten years starting from 2007 (Figure 4). Bovine serum albumin (BSA) and alginate have been used as model compounds of protein and polysaccharides, respectively. It seems considerable research interests exist in BSA, alginate, and HA as RO organic foulants.

On the contrary, although organic foulants with LMWOC, such as surfactants and phenolic derivatives, are known, there are few systematic studies on them. Octanoic acid, representing fatty acids in EfOM, is one of a few cases. The effect of feed solution chemistry (solution pH and Ca^2+^ concentration) on the fouling of RO membranes was investigated [118,119]. Van der Bruggen et al. [120,121,122] investigated a variety of LMWOCs as foulants for NF membranes.

### 4.2. LMWOCs as Foulants in TFC RO

It was shown that specific LWMOCs act as foulants for CA membranes. Small amounts of substituted phenols cause a permeability decrease [123,124]. The fouling was believed to occur by adsorption of medium MW organic molecules inside the pores of the membrane skin. Bromoform and chloroform also cause flux decline in CA membranes [125,126].

Aromatic polyamide (APA) TFC membranes were discovered in 1978 [127] and were filed for patent in the US in 1979 [128]. Because the TFC RO membranes have better silica and organic rejections, a wider pH operating range, and lower pressure requirements, the spiral wound TFC RO elements have been dominantly used for many applications. However, a few harmful organic compounds were identified soon after the development. Table 7 shows examples of foulants caused by LMWOCs observed in the early membrane and application development stages. 

Since the TFC RO membrane has an anionic charge at neutral pH, the membranes are susceptible to binding and fouling by cationic substances such as surfactants and quaternary biocides. During the finding of suitable disinfectants and cleaning agents, the following LMWOCs were found to have adverse effects: iodine, quaternary germicides, and phenolic compounds as biocides and nonionic surfactants as cleaning agents [36]. When testing three types of surfactants, a significant flow decline was observed for the cationic surfactant (Hyamine 3500) and nonionic surfactant (Triton X-100): 77% and 41%, respectively. An anionic surfactant, sodium lauryl sulfate, showed no flow loss. As for the nonionic surfactant, an action somewhat like plasticizers was originally speculated not as foulants [38]. The same flow loss was observed for nonionic surfactants, i.e., polyethylene glycol mono-4-nonylphenyl ether (*n* = 15). Aliphatic amines as amine components for the TFC membranes were proposed to minimize such adverse effects [129]. The TFC RO membrane consisting of an amine mixture of ethylene diamine and piperazine, and trimesoyl chloride (TMC) did not show flow decline. Commercial laundry detergents gave varying results but generally caused some loss of flux.

**Table 7:** Summary of LMWOCs foulants for TFC RO during application development.

Foulant	Comments	References
Quaternary germicides		[36,130]
Cationic detergents	Hyamine 3500 (A blend of alkyl dimethyl benzyl ammonium chlorides)	[38,130]
Phenolic compounds	Phenolic compounds	[36,130]
	Alkyl phenols	[131]
	Nitrophenols and chlorophenols, pH-dependent	[132]
Nonionic detergents	Triton X-100 (Tends to plasticize the FT-30 membrane)	[36,38]
	Polyethylene glycol mono-4-nonylphenyl Ether (n = 15)	[129]
Household soap	Shower/Laundry Water Mixture (Ivory^®^ soap)	[133,134]
Plasticizer	Tributyl phosphate (TBP)	[135]
Formaldehyde	Formaldehyde reacts with residual monomers	[38,130]
	Glutaraldehyde shows a similar phenomenon	[87]
Wastewater effluent	Secondary effluent in WF-21	[38,136]
	UF pretreated secondary effluent	[137]
	Pulp/paper bleach effluents	[138]

Water flux through the TFC RO membrane shows a significant drop with increasing phenol concentration, which cannot be accounted for by higher osmotic pressures of the feed solution. Schutte and Belfort [131] suggested two possible explanations: that the osmotic pressure at the membrane surface may be much higher than in the bulk solution, or secondly, that phenol is sorbed in the very finely porous structure of the membrane and that this causes an increase in the resistance to diffusion of water through the membrane. Bhattacharyya and Madadi [132] investigated separating phenolic compounds from water using the TFC RO membrane (FT30-BW) with various concentrations and feed pH. There was approximately a 50% flux drop for the nonionized 2,4,6-trichlorophenol (TCP) solution at a concentration of 100 mg/L and pH 4. The flux dropped at 31.7% and 72.6% recovery was approximately the same. This showed that the main flux drop occurred at the beginning of the filtration. At pH = 8.6; 100% ionized, there was only an 8.4% flux drop. The flow decline with phenol is much lower than that of TCP under acidic or neutral pH conditions. A more significant flux drop was observed for multi-component systems, such as nitrophenols and chlorophenols in water.

- Phenol: Flux drop = 13.2% (pH: 5.8, feed concentration: 95.6 mg/L)
- Phenol: Flux drop = 7.1% (pH: 7.35, feed concentration: 51.0 mg/L)
- TCP: Flux drop = 48.6% (pH: 4.0, concentration: 101.9 mg/L, recovery: 31.7%)
- TCP: Flux drop = 50.4.% (pH: 4.8, concentration: 94.9 mg/L, recovery: 72.6%)
- TCP: Flux drop = 8.4% (pH: 8.6, concentration: 95.2 mg/L, recovery: 71.0%)
- Note: pKa of phenol and TCP: 9.88 and 5.99, respectively

TBP is widely used as a solvent and plasticizer, particularly for extracting uranium and plutonium from other radionuclides in nuclear fuel reprocessing [135]. The RO tests (2.5-inch diameter SW30 element) containing 120 ppm TBP showed a drop in standardized permeate flow of over 30% after 22 h. However, the fouling was reversible. Cleaning with a caustic detergent solution fully restored the lost standardized permeate flow. 

FT-30 membrane and spiral elements have shown variable results in formaldehyde exposure tests. In spiral elements, losses in a flux of 30 to 50 percent occur within 48 to 72 h of exposure [38]. It was recommended that formaldehyde be used after the element is flushed out for at least six hours to avoid this phenomenon [130]. Aldehydes may react with residual amines or amine end groups in the PA TFC membranes. It is reported that glutaraldehyde is more reactive than formaldehyde [87].

In wastewater treatment, although it is not clear that prominent flow loss can be attributed to the LMWOCs, apparent fouling problems were encountered in TFC RO membranes. FT-30 elements were tested at the Orange County WF-21 in late January 1981 [38]. After a few hundred hours of operation, membrane flux and operating pressure changed from 10 gfd and 200 psi to 7.5 gfd and 250 psi. Simple cleaning trials were ineffective. Another test feeding the secondary effluent of municipal wastewater was conducted in Europe using a plate and frame RO module and UF as pretreatment [137]. It was, however, demonstrated that UF generally does not solve the fouling problem in RO operation. Thus, the contribution of LMWOCs to fouling may be suspected.

The RO treatment of bleach effluents from pulp and paper manufacturing was tested using three TFC RO membranes [138]. Bleach effluents are highly colored and contain high concentrations of chlorides and a range of organic compounds, including phenols, terpenes, resin acids, and lignin degradation products. The feed to the RO was cartridge filtered (10 μm) to remove suspended solids. Membrane fouling was significant, resulting in low membrane fluxes and cleaning only partially restored the membrane flux.

## 5. Symptoms of Fouling by LMWOCs

In actual plants, the LMWOC fouling is usually recognized as a problem of quick flow decline soon after the plant starts up or after a certain period, such as feed water source or quality changes. Troubleshooting efforts associated with such problems have been reported. For example, Sugiyama and Yamaki [139] reported such cases in UPW production systems where feedwater to RO was treated by ion exchange resins. They attributed the flow loss to leachables from system components such as the ion-exchange resin, i.e., cationic and anionic resins. They also mentioned that RO modules sometimes clog with eluted compounds from pipes, fiber-reinforced plastic (FRP) tanks, and concrete pits. Similar cases can be seen in the RO element analysis report [100]. Eleven (11) cases out of 82 samples analyzed were recognized as fouling with absorbed organics that were all fed from the ion-exchange effluent (pure water feed). 

### 5.1. Case Studies of LMWOC Fouling and Troubleshooting Efforts

To better understand typical symptoms of LMWOC fouling, reported case studies are reviewed in this section. 

#### 5.1.1. Peroxide in a Phenol Production Plant [140]

RO separation was considered for treating a process water stream in a phenol production plant. The stream contained several organic compounds such as peroxides (peroxide A and B), phenol, and many different organic acid salts, which demand appreciable processing for reuse or discharge. Different RO membranes were tested in a pilot plant using the actual process mixture and model aqueous feeds containing either single or groups of solutes. In all the RO membranes tested, the presence of “peroxide A” causes a significant decrease in membrane flux. After the initial prominent flow decrease, the flux was stabilized, and no further decline was observed. It was speculated that the specific interactions between the membranes and peroxide A are responsible for such flow reduction. The flux reduction was not completely recovered after a cleaning routine. On the other hand, peroxide B does not cause a significant change in the membrane flux. When tested with TFC RO, peroxide A and B rejections were 55 and 20%, respectively.

Although the article did not disclose the exact chemical names of peroxides, peroxide A might be cumene hydroperoxide, assuming the cumene process for phenol production [141].

#### 5.1.2. Alkylated Diphenylamine Compounds [142]

A manufacturer of ester-based synthetic hydraulic fluids generates wastewater from condensation reactions with various organic chemicals. This waste stream was sent to a hazardous waste treatment facility. In addition to the economic considerations, the company was concerned about the responsibility of off-site disposal of hazardous wastes. TFC RO membranes with MWCO properties of 100–200 Daltons were tested to concentrate hazardous compounds and reduce waste volume to address these concerns. During the tests, it was found that the TFC membrane exhibited a permanent loss of flux. As a result of discussions with the membrane manufacturer, it was concluded that the alkylated diphenylamine compounds in the waste stream that reacted with the membrane polymer caused this flow loss. 

#### 5.1.3. Leachables from RO Elements: Phthalate Esters [143,144]

An intensive effort was carried out to resolve a flux decline problem in a two-pass RO system. The RO system was specified to combine the advantages of both CA and TFC RO elements. The first-pass CA elements performed as expected. However, in the second-pass TFC RO elements, a severe flux decline problem was encountered immediately following start-up. The normalized permeate flowrate of the TFC RO elements immediately began to decline, losing 24.1% of the original flux during the first seven days. After four months of operation, the flux leveled off to about 50% of the original.

The supplier of the TFC RO membrane analyzed the fouled elements to identify the root causes of flow loss. During the course of analysis, several findings were revealed as follows. Conventional high- and low-pH cleanings were unable to restore the flux. However, cleaning the elements with methanol did restore flux to the original design specifications. This result suggested that a methanol-soluble foulant from the first-pass permeate was the culprit. Samples of the methanol were concentrated and analyzed by infrared (IR) spectrophotometry and gas chromatography (GC) in an unsuccessful attempt to identify the foulants. The fouled membrane elements had no noticeable weight gain. Observation of the membrane surface did not reveal any visible foulant. An extract, prepared by soaking samples of membrane and permeate spacer (tricot) from the CA element, was analyzed by GC, which indicated a peak identified as dimethyl terephthalate. Similar peaks were detected in a fouled second-pass TFC RO membrane. Foulant extraction conducted by an analytical company identified dibutyl phthalate (DnBP) as a possible foulant.

A carbon filter was applied to confirm that organic foulants can be removed. No flux decline of the TFC RO elements was observed after ten days of operation. On day 10 of the test, the carbon filter was removed. Immediately, the flux of the TFC RO element began to decline. After seven days, the flux decreased by 17%. These results indicated that the foulants were shed by the CA element and that the activated carbon (AC) removed it.

However, it should be noted that there were some conflicts about leachables from the CA membrane. A CA membrane supplier constructed two additional CA elements using a virgin polypropylene permeate spacer (instead of the standard polyester known to contain phthalate compounds), and these RO elements were installed in a pilot skid. After 15 days of operation, the second-pass TFC RO element had a flux decline of 30%. This evidence suggests that the original standard polyester permeate spacer may not be the source of the foulant.

#### 5.1.4. DnBP from a Reinforced Polyester Pipe [145]

A severe decline in permeate flow rate was recorded shortly after the start-up of a new RO brackish water desalination plant equipped with TFC RO. The normalized permeate flow rate declined by over 40% over the initial 10 days. An extensive water and fouled membrane analyses program indicated that the most likely cause for the flux decline was fouling by phthalate esters, mainly DnBP. The origin of the phthalate ester contamination was traced to the newly installed reinforced polyester pipe (14 km long), conveying brackish well water to the RO plant. 

Laboratory experiments obtained direct evidence confirming that DnBP is a foulant capable of significantly reducing permeate flux. An immediate flux decline was observed after adding one ppm of DnBP to the NaCl solution. During 17 h of testing, the permeate flow rate fell by over 50%. Salt rejection was, however, unaffected. A 0.5% NaOH cleaning in place (CIP) was not effective in restoring the flux. Then, circulating a harsh 1% NaOH solution for about two hours restored the permeate flow to 70% of its original value.

#### 5.1.5. Nonionic Surfactant (Triton X-100) as a CIP Solution [146]

The rapid bench-scale membrane test (RBSMT) was developed to predict the performance of spiral wound membrane elements [88]. The RBSMT can efficiently evaluate membrane performance under various operating conditions while simulating the hydrodynamic conditions in a spiral wound membrane element. A series of tests were conducted to compare the RBSMT’s performance with pilot-scale elements for several NF membranes and source waters.

While a new type of highly charged membrane, NTR-7450, was evaluated, some difficulties were encountered. After the flux dropped 15%, the membrane was cleaned with a manufacturer’s recommended detergent, Triton-X100, resulting in an additional 60% drop in flux for both RBSMT and the pilot systems. Approximately 24 h operation after cleaning, the pilot and bench systems had regained only 50% and 60%, respectively, of the flux lost during cleaning, indicating that the cleaning resulted in significant irreversible fouling of the membranes. Next, a detergent in an acid solution was recommended, and again, a 60% drop in flux was observed for both systems after cleaning. After this event, the optimal cleaning solution was identified as a 2% solution of sodium lauryl sulfate, adjusted to a pH of 11.0 with sodium hydroxide.

It was known at the time that Triton-X100 could be used as a CIP chemical for the CA membranes but is incompatible with the TFC RO membranes [38,147]. Thus, it became clear that Triton-X100 is incompatible with the anionically charged NF membrane. 

#### 5.1.6. Bisphenol A (BPA) Leached from an NF Permeate Spacer [148]

A hybrid NF-RO two-pass process was proposed and started to treat ground and surface waters contaminated with ammonium sulfate (amsul) in a metal refinery site. The first pass used NF membranes to produce an amsul-rich concentrate for the evaporators. NF permeate was then concentrated by a PA RO and forwarded to newly constructed double-lined evaporation ponds.

Within hours of start-up, the RO membrane permeability was observed to have decreased. The flow rate had fallen to 50% of the starting value after the first few months of operation. This phenomenon was unexpected as the NF permeate fed to the RO plant was considered superior quality. Several investigations were undertaken, including membrane autopsies and plant tests. Small sections of flat sheet membrane inserted in the feed line to the RO plant showed a rapid drop of 50 to 55% of the predicted flux. Passing the RO feed through an AC bed successfully prevented the flux loss for the flat sheet tests. Investigations suggested that the likely foulant was BPA, which had leached from the NF permeate spacer. As a corrective measure, additional RO elements were installed in the first stage, and a packed bed AC filter was installed in the feed line. 

#### 5.1.7. Unknown Organic Compounds Present in the Demineralized Water [149]

A new hybrid NF-RO two-pass system was installed to concentrate cooling water blowdown. After the membrane system started up, the water permeability of the RO unit decreased with time. One of the chemicals added to the cooling water was suspected to cause the flux decline. However, it was not clear whether this caused the flow decline. Thus, the cartridge filters immediately upstream of the RO elements were replaced with AC-coated cartridge filters, but this did not help much.

Small household RO elements operated on about 24 different water streams in the plant. The water stream that caused the flux loss of the RO unit was found to be the demineralized water. Since demineralized water at 40–50 °C was easily available at the site, this water was used for membrane flushing and cleaning solutions. According to the findings, organics originating in the condensates mixed in with the fresh make-up water upstream of the ion exchange equipment fouled the RO membrane.

#### 5.1.8. Aliphatic Halogenated Hydrocarbons in Feed Water [150]

Boiler feedwater taken from river water has been treated by the TFC RO elements since 2004. Originally, it had two trains, but a third train was added in 2009 to increase the capacity. For the first train, the flow rate decreased dramatically during the first month of operation compared to the projected start-up performance. Conventional cleaning methods were ineffective, but with dichloroisocyanurate, the flow was recovered. This oxidative cleaning was applied thrice but discontinued because it negatively affected salt rejection.

When the new third train was started in 2009, a similar flux decline was observed, although at a slower rate, followed by a period of stable performance. This kind of initial flux loss was observed with all membranes. Aliphatic halogenated hydrocarbons have been identified on the membranes; such substances are known as strong membrane foulants. A possible origin is the wastewater from the site blended into the pretreatment section. Slight flow recovery was seen with cleaning at pH 13, but based on previous experience, the oxidative cleaning was not applied. The positive side effect of that fouling was an increase in the system salt rejection from a projected value of less than 99% to about 99.7%.

### 5.2. Typical Symptoms of LMWOC Fouling

Organic fouling occurs when specific LMWOCs come into contact with RO/NF membranes. The following typical symptoms can be seen for LMWOC fouling when reviewing the reported case studies and fundamental research results.

- Sharp flow decline within a short time of contact;
- Salt rejection sometimes increases;
- No appreciable differential pressure increase;
- Feed organic concentration is very low, a few ppm or less;
- No visible foulant can be seen when fouled elements are autopsied;
- Flow loss is sometimes irreversible or difficult to restore through regular cleaning;
- Methanol (50%), high concentration of nitric acid, or oxidative cleaning are needed to restore performance;
- AC pretreatment is effective in alleviating problems.

#### 5.2.1. Initial Flow Behavior

**Figure 5:**
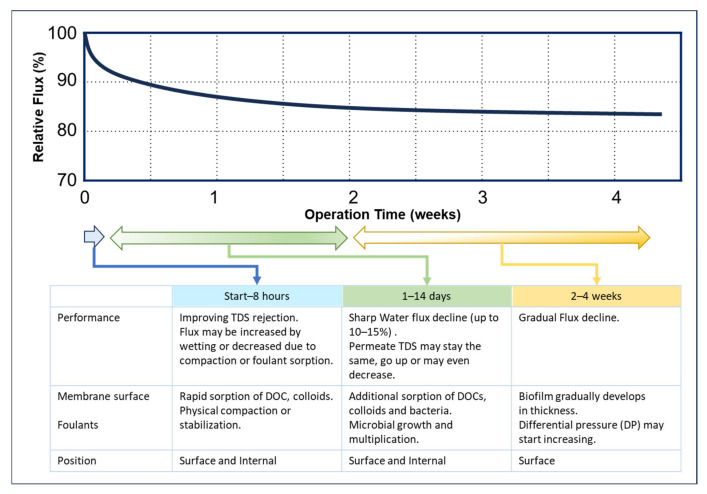
Water flux as a function of operation time and typical events in one month of operation.

In RO/NF plants, some flow decline is expected. Paul and Rahman [8] explained relative flow changes and typical events during operation, i.e., three phases. Khan et al. [151] also proposed a similar concept of fouling development as a dynamic phenomenon involving different phases in the progressive development of a fouling layer. According to their phased fouling development concept, a schematic image of flow decline and fouling events is shown in Figure 5. 

Here, initial flux decline is attributed to the rapid sorption of DOC, deposition of colloids, and physical compaction. Since some RO/NF elements are shipped in non-stabilized or dry conditions, these elements may show a relatively prompt flow decline [88,152]. It is said that dry membranes tend to start at a slightly higher flow [96]. This is not a case of membrane fouling. In the second phase (1–14 days), water flux may continue to decline (up to 10–15%) depending on pretreatment conditions and foulant types. In the third phase, the permeate flow tends to be stabilized.

As for LMWOC fouling, the most critical phenomenon is a sharp flow decline after contact with feed water. Thus, knowing whether other foulants might show similar prominent initial flow decline is essential. Many researchers reported fouling phenomena by using model compounds such as BSA [153], alginate [154], HA [155,156], silica colloids [157,158], and actual surface water and wastewater. It can be seen that some foulants show sharp flow declines under specific conditions [159,160]. The following are such cases:

- High concentration of colloids (e.g., aluminum oxide and silica) [161,162];
- HA under low cross-flow velocities and high initial flux. Ca^2+^ ion also plays an important role in this case [155,156];
- Alginate with under high initial flux [154].

It is well known that several operational factors significantly influence initial fouling rates, including initial flux, cross-flow velocity, ionic strength, foulant concentrations, other components like Ca^2+^ ion, pH, etc., and membrane characteristics. Among those factors, the initial flux profoundly affects initial fouling behavior [155]. Tang et al. [156] evaluated the effect of the initial flux on the flux reduction by using HA (5 mg/L). At lower flux levels (41.7 L/m^2^/h (LMH)), membrane flux reductions were less affected by solution chemistry, and the stable fluxes were more reflective of the intrinsic clean membrane fluxes. The same behavior was observed for the colloidal silica fouling [157].

Incidentally, no fouling was observed for the smooth surface piperazine polyamide (PPA) NF membranes, even with a very high silica concentration of 200 ppm. In the case of TFC RO, heavy flux decline was seen under the higher flux conditions depending on the type of membranes. However, under low flux, like 17 LMH, flux loss was minimal [158].

**Figure 6:**
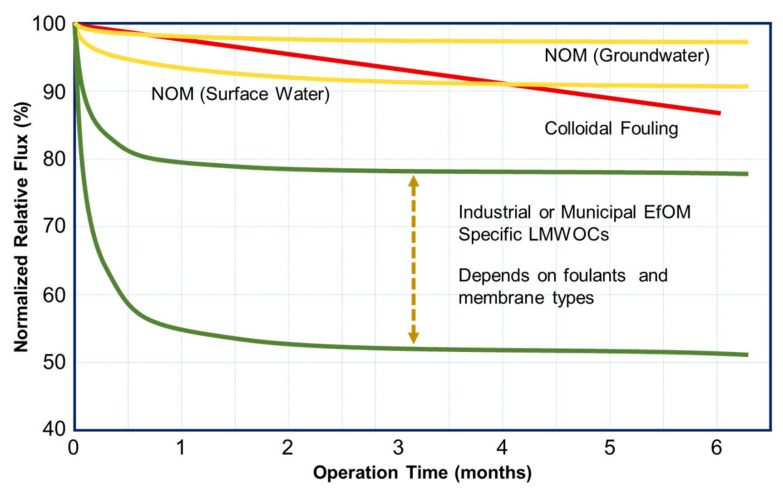
Schematic normalized relative flux changes in actual RO/NF plants with different feed sources.

Actual RO/NF plants have been typically designed and operated at an average flux between 14–15 LMH (seawater, wastewater), 17–20 LMH (municipal wastewater), and 30–35 LMH (second-pass RO). Therefore, there might be some discrepancy between the lab test results and actual plant performance for the initial fouling propensity. Reported data and case studies were reviewed to elucidate the fouling behavior of actual plants and pilot tests. According to that information, the flow rate changes for (i) high NOM concentration waters, (ii) colloidal fouling cases, and (iii) wastewater and LMWOC fouling cases during the first six months are schematically shown in Figure 6.

RO/NF plants treating high NOM concentration groundwater and surface water show less initial flow decline and fouling in many cases, as seen in Figure 6. For example, when treating a slightly brown color and high TOC well water, the total normalized flow rates dropped slightly by about 8% over the first year [163]. After one year of operation, a standard caustic and acid cleaning was undertaken. The cleaning solutions were analyzed at various intervals over a one-hour recycle of cleaning solution. Although the feed water contains about 5 to 10 ppm TOC, the organic matter apparently did not foul the membrane since less than 2 ppm of TOC was found in the cleaning solution even after one hour of recycling. Similar results can be seen in a 1-year NF membrane pilot study in southern California to evaluate DBP precursors and color removal (11.1 mg/L TOC) from groundwater with color values of up to 200 color units [164].

It is reported that Ca ion significantly impacts HA fouling. An NF test with conventionally pretreated groundwater with elevated hardness (Ca^2+^: 115 mg/L; Mg^2+^: 12 mg/L; DOC = 2.9 mg/L) was carried out in a German water treatment plant to elucidate the effect of hardness [165]. Within an operation period of 4 weeks, no significant fouling occurred. However, at 85% recovery, the feed DOC and Ca ion concentrations were increased to 20.4 mg/L and 329.6 mg/L, respectively, due to concentrate recycling. This condition caused a gradual decrease in the flux over the first four days, and then the flux stayed constant. A gradual growth of the gel layer at the membrane surface explained the decrease in flux during the first four days. Another case is seen in a water treatment plant in Canada, where groundwater contains high levels of iron, manganese, TOC, and hardness [166]. Biofilters remove iron and manganese, but high TOC (8 mg/L) and hardness (647 mg/L) remain in the feed to the hybrid RO-NF units. It was reported that no evidence of discoloration by precipitated Fe/Mn oxides, biofouling, or precipitates reaching the membranes was observed after 210 days of RO operation.

For surface sources, water quality might deteriorate compared with groundwater due to the effects of mankind’s activities and the watershed environment. Thus, RO/NF plants are expected to be more susceptible to fouling when using a surface water feed. There are many municipal and industrial RO/NF plants that treat surface water. Pilot tests have been conducted to obtain information on membrane performance and fouling prevention. For example, with support from the AWWA Research Foundation (AWWARF), two NF membranes with MWCO of approximately 200 to 300 Daltons were examined for ultrafiltered surface water [167]. The source water has 301 mg/L TDS, 5.2 mg/L TOC, and 12 NTU turbidity. The NF membranes were operated at a constant flux rate of 30.5 LMH (18 gfd 20 °C). Specific flux profiles for two NF membranes were stable during 100 days of operation, although a slight flow decrease was observed. Similar results were seen in other plants [168,169]. One such case was for an NF pilot test treating lake water with high color (45–55 mgPt/L) content. The permeate flow was stable for the first 60 days [168].

**Figure 7:**
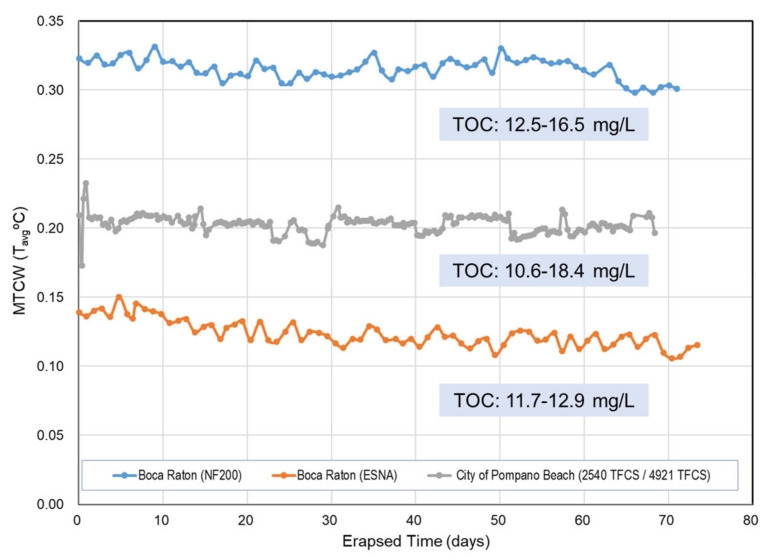
The MTCw changes for high TOC groundwater during the ICR pilot tests.

The US Environmental Protection Agency (USEPA) Information Collection Rule (ICR) requested certain utilities to conduct DBP precursor removal studies [170]. The utilities investigated either granular AC or membrane processes in the bench- or pilot-scale to evaluate the impact on potable water treatment. The fourteen pilot-scale studies conducted included six surface water and eight groundwater feeds. The pilot-scale membrane test data are available in the ICR Treatment Study Database [171]. When reviewing the data, neither surface water nor groundwater showed any sharp flow decline. A part of the pilot test data (water mass transfer coefficients, MTCw) is shown in Figure 7, along with feed TOC levels.

For the second case, Handley [172] reported the linear specific flux changes due to the mixed colloidal (silt and iron oxide) and organic fouling in a municipal water treatment plant in California. The facility’s feed water is sourced from brackish groundwater wells containing high manganese concentrations. The feedwater is treated through an iron and manganese removal system. A steady specific flux decline has been observed at the facility since its startup in 2010. When the fouled elements were autopsied in 2013, the foulant was found to be 43% inorganic and 57% organic material based on a LOI test. The inorganic foulant was composed of iron, silicon, calcium, and aluminum. This composition is consistent with silts and clays with some iron oxide. This case is an example of the normalized flow being linearly decreased.

The third case is one in which the flow rate decreases immediately after starting the operation. Some examples have been introduced, including nonionic surfactants, plasticizers, etc. The fouling behavior of octanoic acid as a model compound of fatty acids differs significantly from that of BSA, HA, alginate, and colloids [118]. The flux loss is almost instantaneous at feed pH 3.9. The same result was observed for a nonionic surfactant (alkyl phenyl ether type: C9+Phe+EO8.5) [173]. Within a few hours of operation, the initial flux decreased by 60% from 41.7 LMH (1.0 m/d) to 16.7 LMH (0.4 m/d). For actual RO/NF plants, such a sudden flow decline phenomenon is observed in municipal and industrial wastewater plants [174,175,176]. For municipal wastewater treatment, such sharp initial flow decline was observed at the Groundwater Replenishment System (GWRS) in the Orange County Water District (OCWD) [174] and the West Basin Municipal Water District (WBMWD) [175]. OCWD has continued to evaluate RO membranes to qualify multiple RO membranes for use in the GWRS. Many RO elements were assessed using the first-stage vessels operating at 25.2 LMH (14.8 gfd) and 55% recovery. Typical feed TDS and TOC are 884 mg/L and 7.9 mg/L, respectively. Severe flux loss was seen soon after the pilot start-up, and the flux was stabilized after 1–3 months of operation for almost all elements tested. Rigorous cleaning could restore the flux somewhat, but the flux decline continued thereafter. Similar phenomena were observed in an industrial (steel manufacturer) wastewater plant [177]. Even after rigorous pretreatment consisting of sedimentation, sand filtration, and UF, the feed water to the RO had high COD (35.4 ppm) and TOC (9 ppm). After 20 days of operation, the normalized permeate flow was decreased and stabilized at certain levels depending on membrane types. Since it is thought to contain a certain level of harmful LMWOCs in the EfOM, it might be reasonable to consider that the initial sharp flow loss is due to the LMWOCs.

Xu et al. [160] summarized the flux change of RO membranes treating various waters, including municipal secondary effluent, treated industrial wastewater, surface water, and groundwater. These normalized flux changes are consistent with the trends shown in Figure 6. Industrial wastewater’s initial flow decline rate is especially fast compared to any other feed type. It is reported that the normalized flux (J/J_0_) is decreased to 0.1–0.3 in specific cases.

#### 5.2.2. Other Symptoms

Other key symptoms include difficulty cleaning with common CIP chemicals and detecting organic foulants from membrane surfaces or feed solutions. Sorbed foulants to the membrane surface in a monomolecular layer are hard to identify and remove [35]. Tasaka et al. [100] reported that the direct FT-IR analysis of the membrane surface did not detect foulants due to their minute quantities. However, the FT-IR analysis could identify a nonionic surfactant by extracting foulants with hexane from the hydrochloric acid solution used to clean the membrane. Similar efforts were reported for fouled membranes from a municipal wastewater treatment plant [176].

## 6. Other Relevant LMWOC Foulants

The previous sections showed that certain LMWOCs induce significant flow reductions, but not all LMWOCs behave as RO foulants. For example, lower alcohols, lower fatty acids, mono- and disaccharides, etc., do not cause a significant decrease in flow rate, except for the effects of osmotic pressure and concentration polarization. Ethylene glycol (EG) and propylene glycol (PG) were previously used as additives for preservatives for RO/NF membranes. Citric acid and sodium dodecyl sulfate (SDS) are used to clean membranes. On the other hand, although benzene and phenol are hydrophobic and may be adsorbed to the membranes, no significant decrease in flow rate was observed [178]. 

This section summarizes typical LMWOC foulants, including those discovered in basic research investigating the removal effectivity of LMWOCs.

### 6.1. Surfactants

Surfactants are one of the most ubiquitous and vital families of organic compounds. They have two essential properties: their ability to lower the surface or interfacial tension and their capacity to solubilize water-insoluble compounds. Thus, surfactants in different formulations have been used in many industries like cosmetics, personal care, household, painting, coating, textile, dyes, polymer, food, agrochemicals, oils, microelectronics, etc. [179,180].

Due to their wide range of use, surfactants are encountered in sewage and industrial wastewater at different concentrations [181,182,183,184,185,186]. Since cationic and nonionic surfactants have been known as potent organic foulants [36,38,129,146,147], this should be a critical issue when treating surfactant-containing wastewater by RO/NF. For example, nonionic surfactants are frequently used in cleaning flat panel display (FPD) components [187,188]. Severe flow loss is sometimes observed when treating wastewater from FPD production [189]. Under these circumstances, extensive research has been conducted from the following points of view and summarized in Table 8.

- Fouling mechanisms: effects of charge, membrane types (RO/NF), etc.;
- Model compounds associated with low fouling membrane development;
- Membrane surface modification for enhancing fouling resistance [190,191,192];
- Compatibility in membrane cleaning;
- Treatment of surfactant-containing industrial process waters and wastewaters;
- Prevention of fouling by surfactants.

**Table 8:** Summary of a research area in RO/NF fouling by surfactants.

Membrane Type	Surfactant Characteristics	Filtration Conditions
RO/NF Membranes - APA (RO, NF) - PPA (NF) - CA (RO, NF) - Others (NF)	Nonionic surfactants Alcohol ethoxylates (AEO) Alkylphenol ethoxylates (APEO) Anionic surfactants Linear alkyl benzene sulfonate (LAS) Lauryl ether sulfate (LES) Cationic surfactants Dodecyltrimethylammonium bromide (DTAB) Cetyltrimethylammonium bromide (CTAB) Zwitterionic surfactants Cocamidopropyl betaine (CAPB)	Concentration Below CMC Above CMC pH Temperature Flux (LMH) TDS
	Hydrophobic portion Aliphatic, alkylbenzene Perfluoroalkyl Polysilicate	
Charge/Charge density Surface free energy (Contact angle) Surface roughness MWCO	Linear or branched type Molecular weight Critical micelle concentration (CMC) Hydrophilic-lipophilic balance (HLB) Effect of mixture constituents	

The degree of fouling is influenced by membrane characteristics (materials, charge, surface roughness, etc.), surfactant properties (ionized or non-ionized, HLB), and operating conditions (concentration, flux). The following subsections discuss key factors affecting the degree of fouling.

#### 6.1.1. Effects of Surfactant Types, Membrane Materials, and Their Charge

Adverse effects from surfactants were first recognized with the TFC PA membranes [38], but flow decline was also observed with other membranes, such as polybenzimidazolone by nonionic surfactants [193]. A comprehensive investigation of the effect of cleaning agents, including various surfactants, was conducted by Ishida et al. [194]. Seven commercial RO membranes and one experimental membrane were evaluated. Each membrane was exposed to 37 chemical compounds with various chemistries. This select group of compounds included nonionic, cationic, anionic, zwitterionic (i.e., an overall neutral compound with localized positive and negative charges), chelating, and oxidizing chemical cleaners. Any surfactants had a minimal effect on the water flux of the CA membrane. The average changes in water flux (L/m^2^·day) of the seven TFC RO membranes for anionic, cationic, nonionic, and zwitterionic surfactants are −0.34, −1.62, −1.38, and −0.79, respectively. As for all five of the PA membranes, the cationic compounds benzalkonium chloride and cetylpyridinium chloride and the nonionic compounds, that is, polyethylene glycol dodecyl ether, polyethylene glycol lauryl ether, polyoxyethylene (20) sorbitan monolaurate, and polyoxyethylene (20) sorbitan monostearate caused the most significant flux decline. Along with the decline in flux, salt rejection increased when cationic and nonionic surfactants contacted the membranes. They caused the membranes to tighten up. Similar results were seen in other membrane systems and are summarized in Table 9. 

First, the CA membrane was not affected by any types of surfactants either. As for anionic surfactants, a noticeable flow decline was not observed for almost all RO/NF membranes except the aromatic polyurea membrane (NTR-719HF), which has positive charges. Inversely, negatively charged membranes show significant flow loss with cationic surfactants. Thus, it can be said that combinations of cationic membrane with anionic surfactant or anionic membrane with cationic surfactant result in significant flux decline [196]. The NTR-729HF membrane showed less fouling for three types of surfactants. NTR-729HF, which has a neutral and hydrophilic polyvinyl alcohol structure, was thought to be resistant to fouling by surfactants. Since the TFC PA membranes have amphoteric characteristics, their charge and charge density are affected by feed pH. At neutral or higher pH, the PA membranes have negative charges; at low pH, the membranes have positive charges. Thus, a more significant flux decline was observed for the FT30 membrane by adding SDS at low pH [198]. The influence of surfactants on RO membrane properties and the rejection of trace nuclides in low-level radioactive wastewater was evaluated [199]. After a 12 h fouling test using surfactants SDBD, Tween-80, and CTAB, the permeate flux declined by ~55.8%, 44.86%, and 40.61%, respectively. Because the feed water contains 500 mg/L of boron, SDBD may have shown significant flow loss due to the expected lower pH. In addition, by adding SDBS and Tween-80, the boron rejection increased ~12.5% and 18.1%, respectively.

Halleb et al. [181,200] investigated the effect of surfactant concentrations ranging from 10 mg/L to 10,000 mg/L. The relative flux of the PA membrane was decreased with increasing surfactant concentration up to 50 mg/L. However, over this concentration, the permeate fluxes from all types of surfactants (SO, TW20, and CTAB) remained almost stable. This phenomenon means surfactant micelles do not contribute to additional colloidal fouling. Surface adsorption on the PA membrane surface may reach almost equilibrium at lower concentrations than CMC, and stable flux was observed in a wide range of surfactant concentrations.

Fluorinated surfactants are a unique class of compounds characterized by their hydrophobic tail, which is either partially fluorinated or replaced totally with fluorine molecules [201]. Perfluorooctane sulfonate (PFOS) is a fluorinated surfactant with an eight-carbon perfluorinated alkane with a sulfonate group at one end. In the semiconductor industry, PFOS was used in multiple photolithographic chemicals. However, due to growing health concerns, its usage in this sector has been discontinued. One of the current issues on poly- and perfluoroalkyl substances (PFAS) is how to remove sub-ppb levels of PFAS from contaminated surface and well waters [202]. Thus, fouling with PFAS may not be an issue in such low concentrations.

Meanwhile, high concentrations of PFOS were detected from wastewater in a semiconductor plant. Tang et al. [203,204] evaluated the fouling phenomenon for APA and PPA TFC membranes. Although the actual wastewater pH ranged from 2.85 to 3.0, the pH of the testing solution (10 ppm of PFOS) was adjusted to 4.0. Heavy fouling is expected since all membranes have positive charges at this pH. However, flux decline was less than 16% at high flux conditions. It was also found that the flux decline rates depend on initial flux but vary with each membrane type. Under the same initial flux (1.8 m/d, 75 LMH), NF90 and ESPA3 exhibited greater flux reduction, and the PPA membranes (NF270 and DK) showed less flux losses. The high flux membrane ESPA3 was more sensitive to feed concentration than the other membranes. While the flux reduction for ESPA3 was 10% at a concentration of 10 ppm, it increased to 60% at 500 ppm PFOS.

Surfactant fouling tendency has been mainly evaluated under the following conditions: monitoring flux after adding surfactants or measuring flux before and after membranes are statistically or dynamically contacted. Another method is to evaluate the fouling irreversibility. The fouled membrane was washed by circulating pure water. The irreversible fouling can be estimated by comparing the pure water flux of the initial and fouled membrane. Ikeda et al. [205] conducted these tests with 10 ppm and 1000 ppm of surfactant concentrations. At 10 ppm of concentration, the flow decline of AEO (MW 1040) was the same as the anionic surfactant (SDBS), both showing about a 12% decrease. However, at 1000 ppm of concentration, pure water flux was decreased to about 32% compared with non-fouled membrane (ES-10). Simple pure water flushing was insufficient to restore the flux at higher surfactant concentrations. Such irreversible fouling was more prominent for cationic (benzalkonium) and amphoteric (n-dodecyl-N,N-dimethylglycine) surfactants. The same results were obtained for the following surfactants: CTAB, SDS, and Triton X-100 [206].

#### 6.1.2. Effects of Nonionic Surfactant Chemical Structure

Electrostatic interactions play a significant role in anionic and cationic surfactant fouling. However, other mechanisms must be considered for nonionic surfactants, such as hydrophobic interactions, membrane surface roughness, membrane pore structure (e.g., pore size), etc. For example, ULP RO membranes experience more profound flow loss with nonionic surfactants. The ULP RO membranes are thought to have rougher membrane surfaces and larger pores than LP RO membranes [207]. Fusaoka et al. [208,209] reported fouling test results using ULP, LP, and surface-modified low-fouling RO membranes. Although the ULP RO membrane initially has a large flux under the surfactant-free condition, the flux decreases by 47% after being fouled with 100 ppm of octylphenol ethoxylate. On the other hand, a flux decreases by 36% for the LP membrane and 27% for the low-fouling membrane. 

Apparent differences in fouling nature for nonionic surfactants were observed between APA and PPA membranes. As seen in Table 9, the PPA NF membrane (NF270) has a better tolerance for nonionic surfactants. This low-fouling nature is consistent with the disclosed PA membranes consisting of aliphatic amines and aromatic acid halides. For example, the TFC RO membrane, which consists of an amine mixture of ethylene diamine and piperazine, and TMC did not show a decline in flow [129]. Several research studies exist to explore the effects of NF membranes on nonionic surfactants, which are summarized in Table 10.

The exact membrane chemistry of the Desal 5DL and 51HL is not well known. However, it is reported that these NF membranes have an active layer made of PPA [212]. Assuming this is correct, NF membranes listed in Table 10 are classified into PPA and PES-based. All PPA-based NF membranes show less decline in flux. In the case of the UTC-20, a prominent flow increase was observed after contact with the surfactant.

On the other hand, the PES-based NF membranes showed a significant decrease in flow. After the feed surfactant solution was replaced by deionized water, flux was restored to the original pure water level for NF270 [210]. However, for NTR7450, fluxes stayed low. Thus, fouling is considered to be irreversible. The hydrophobic interaction between membranes and nonionic surfactants might explain this phenomenon. The PPA membranes have lower contact angles, that is, they are more hydrophilic compared with PES-based ones [213,214]. These fouling characteristics are consistent with UF membranes. Nonionic surfactants adsorb strongly to pores of UF membranes, thus causing flux decline, especially for hydrophobic membranes [215].

**Figure 8:**
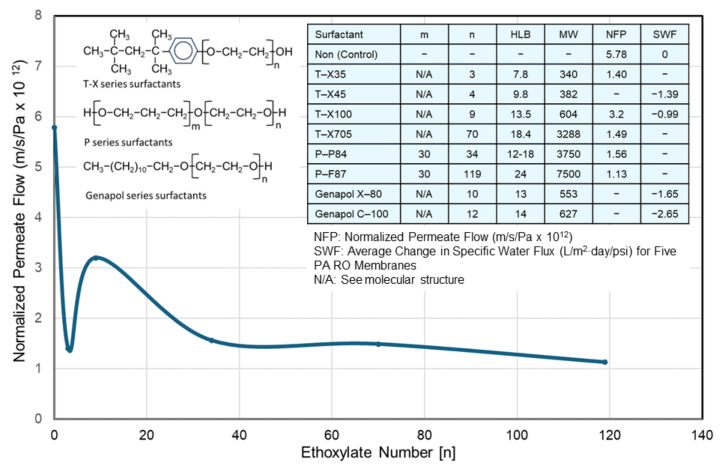
Normalized permeate flow (NFP) as a function of ethoxylate number [192] and average change in specific water flux (SWF) [194].

Surfactants are amphiphilic molecules that have hydrophobic and hydrophilic components. Thus, the following factors must be considered when considering the fouling behaviors and mechanisms: the HLB value, hydrophilic- and hydrophobic-chain length, etc., as shown in Table 8. Wilbert et al. [190,191,192] evaluated the effect of nonionic surfactants on improving the fouling resistance of RO membranes. However, unlike the original purpose, treating the surface of the APA RO membranes with any surfactants caused a decline in flux. A homologous series of commercial polyethylene oxide (PEO)-based surfactants, with either alkylphenol or polypropylene oxide “head” groups, were tested for commercial samples of CA blend, APA TFC RO, and NF membranes. After soaking the RO/NF membranes in 0.1% surfactant solutions for 24 h, membrane performance was evaluated. The results of the APA membrane are shown in Figure 8, together with the results reported by Ishida et al. [194].

A significant flow decrease was observed for the T-X35, which had a low HLB value of 7.8. However, the T-X100, with a HLB value of 13.5, showed less flux decline than the T-X35. The T-X705, with a higher HLB number (18.4), again reduced the flow rate, similar to that of the T-X35. No apparent changes in flow decline were observed by further increasing the degree of ethoxylation. The same results were seen in T-X45 and T-X100, where SWF was −1.39 and −0.99, respectively. The T-X100 induced the least flux decline among nine nonionic surfactants [194]. This fouling behavior might be interpreted that hydrophobic interaction is important for lower HLB surfactants. For higher HLB surfactants, longer chains of ethoxylates may have more substantial interaction with RO membranes.

Kawakatsu et al. [173,216] conducted fouling tests with nonionic surfactants to investigate the effect of hydrophobic and hydrophilic portions on LP RO membrane fouling. They used two types of nonionic alkyl ethoxylate (AE) surfactants: C12+EO7 (the alkyl chain contains twelve carbons and seven ethylene oxide units.) and C12+EO20. A rapid flux drop was observed from an initial flux of about 1 m^3^/m^2^/d. The stabilized permeate fluxes are shown in Table 11. The stabilized flux rates of AE(C12+EO7) were 0.80, 0.55, and 0.23, respectively, after applying 0.1, 1, and 10 mg/L of surfactant solutions. A similar RO membrane test was conducted using 1 mg/L aqueous solutions of fatty acids (C12), PEG (EO20), and AE (C12+EO20). The resulting stabilized fluxes were 0.85, 0.67, and 0.42 m^3^/m^2^/d, respectively. Fatty acid (C12) as a hydrophobic portion of the surfactant does not significantly reduce the permeation flux. On the other hand, PEG (EO20), a hydrophilic portion of the surfactant, showed a more significant flux decline than that of fatty acid. The AE, consisting of both, reduced the flux the most. 

Next, to see the effect of PEG molecular weight, three PEGs, PEG106 (molecular weight: 106), PEG1470, and PEG7100, were tested. PEG106 did not affect the flux, and the higher molecular weight PEG largely decreased the flux at lower concentrations (PEG1470: 1 ppm, PEG7100: 0.1 ppm). Since the effect of alkyl groups on the flux was not so strong, it was concluded that the strongly adsorbed ethylene oxide group causes significant flow reduction. Furthermore, the hydrophilic PEG was indicated as a stronger foulant when the molecular weight increased.

A sharp flux decrease is observed for cationic surfactants due to electrostatic interaction. However, for polycation, e.g., PEI, no appreciable flux decline was observed, as shown in Table 9 and a disclosed patent [217]. The presence of adsorbed PEI was confirmed by ESCA analysis [218]. Thus, Ikeda [195] speculates that their effects on reducing flux might differ, although PEI and cationic surfactants have positive charges. Hence, it might be meaningful to compare the fouling behavior of polycations with PEGs. 

Polycations such as cationic flocculants have been thought to harm TFC RO membranes [147]. For instance, an immediate decline in permeate flux was reported after dosing 1 ppm of cationic flocculant (Hiset C-200) [219]. Therefore, some types of polycations seem to significantly reduce the flow rate of RO membranes while others do not [220].

**Figure 9:**
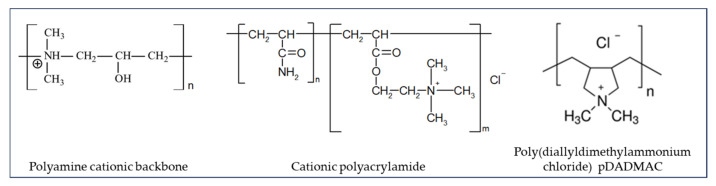
Molecular structure of polycationic flocculants.

Chesters et al. [221,222] investigated the effect of cationic flocculants on RO fouling. It was found that polyamines having cationic charges within a central backbone, as shown in Figure 9, are less fouling to RO membranes. Meanwhile, cationic polyacrylamides tend to attach their cationic pendant group to the anionically charged membrane surface and membrane pores like hook-and-loop fasteners attaching to the recipient surface. Polyamines with a central charge on a backbone are considered to straddle membrane pores and not penetrate them. PolyDADMAC, having a bulky cationic group within a polymer backbone, does not show appreciable flux decline either [223].

It is known that polyelectrolyte complexes are formed by simply immersing anionic-charged membranes in polycation solutions [224,225,226]. By utilizing this technique, layer-by-layer (LBL) membrane surface modification has been investigated. A polycation and a polyanion are alternately deposited on a substrate and are adsorbed by electrostatic interaction. The deposition of polyelectrolytes on a commercial RO membrane via LBL assembly was reported, and poly(sodium 4-styrenesulfonate) and poly(allylamine hydrochloride) were used as polyelectrolytes [227]. The permeability was decreased from 4.6 to 3.1 L/(m^2^·h·atm) with six bilayers and to 2.5 L/(m^2^·h·atm) by coating twelve bilayers due to increased hydrodynamic resistance in the membrane. It should be noted that even with a thick coating (12 bilayers), the permeability reduction was about 50%.

This behavior differs significantly from the fact that a 70% decrease in flow rate was observed with 1 ppm of PEG solution. When observing the adsorptive fouling by polycations and increased flow resistance with LBL surface deposition, PEG fouling cannot be solely attributed to surface adsorption. In addition, internal fouling (pore constriction and blockage) must also be considered. Since PEG is a linear hydrophilic polymer, the ethylene oxide chains may penetrate the PA membrane matrix by a snake-like movement and be permanently trapped due to their low diffusivity. This interpretation could apply to the fouling behavior of ethoxylated nonionic surfactants.

#### 6.1.3. Model Compounds for Low-Fouling Membranes Assessment

Surfactants are used as model foulants along with HA, proteins, and polysaccharides in the development and validation of low-fouling RO/NF membranes. The process typically involves comparing newly developed membranes with regular commercial membranes as a reference. Table 12 provides an overview of surfactants used in evaluating low-fouling membranes, particularly those prepared by surface modification. Non-charged PVA and PEG are frequently applied to make membranes less charged. Consequently, cationic surfactant DTAB may have been utilized to confirm that the electrostatic interactions are minimized. The PVA-coated NF membrane showed less flux decline for benzalkonium chloride [228]. However, no prominent effect was observed for HA and protein (bovine serum albumin), although less fouling characteristics were observed for a calcium-added HA system.

Membrane manufacturers tend to utilize nonionic surfactants to prove their low-fouling RO membranes. There may be a gap between the needs of industry and the research targets in academia.

#### 6.1.4. Surfactant Fouling in Various Industrial Wastewater Reclamation

Surfactants are widely applied in many industries for production and cleaning processes. Due to their wide usage range, surfactants are frequently observed in sewage and industrial wastewater [181]. Thus, when RO/NF is applied to reclaim such wastewater, fouling problems might be inevitable. Table 13 summarizes some examples of surfactants containing industrial wastewater treated by RO/NF.

Since these wastewaters contain many organic and inorganic contaminants, it has been reported that the fouling behavior is different compared with the case of a surfactant present alone. Srisukphun et al. [254] investigated the effect of foulant interaction between surfactant, reactive dye (anionic dye), and EfOMs in textile wastewater on RO membrane fouling. The EfOMs were prepared by filtering an activated sludge through an MF membrane with a pore size of 0.45 μm. Interestingly, aggregation between EfOMs and dye, EfOMs and surfactants, and dye and non-ionic surfactant improved the permeate flux of the RO membrane compared with the wastewater containing a single foulant. Further, not only effluent constituents but also pretreatment chemicals have some impacts on surfactant fouling. It was reported that antiscalant (organo-phosphorous) dosing to a model effluent containing LAS and a commercial detergent reduced fouling intensity to some extent [258]. The relative flux decline rate was improved from 72 to 56% by adding 8 mg/L of the antiscalant.

One of the measures to avoid severe surfactant fouling problems in industrial wastewater treatment is to use low fouling membranes such as the PPA NF membranes. However, even though using such membranes (e.g., NF270), severe flow decline was observed when filtering a particular rim cleaner (nonionic surfactant-based) [260]. Thus, another (less fouling) rim cleaner was suggested to replace the alkaline rim cleaner. Another idea is to eliminate the nonionic and cationic surfactants from industrial effluents by oxidation and granular activated carbon (GAC). Prior to supplying the wastewater to RO/NF, activated sludge biological treatment and solid-liquid separation are commonly applied. Sugiyama et al. [267] found a specific strain that degrades nonionic surfactants quickly and uses nonionic surfactants as the sole carbon source. A laundry wastewater recycling facility utilizing such a strain in the MBR process was developed. Another approach is to use membrane-compatible nonionic surfactants [268,269]. It was found that the Guerbet alcohol ethoxylates comprising branched alkyl groups (i.e., R_1_-(OC_2_H_4_)_n_—OH, wherein R_1_ is a branched C_8_ to C_10_ alkyl group and n is from 2 to 10) instead of linear alkyl groups have better membrane compatibility [269]. Although the membrane compatibility data are only shown for UF treatment, less fouling propensity could be anticipated for RO/NF membranes. In the case of linear nonionic surfactants and PEG, the passage of surfactants and PEGs with molecular weights much higher than the MWCO of RO/NF membranes was observed in an oil-field produced water treatment [266]. These findings imply that the linear nonionic surfactants and PEGs could penetrate the membrane polymer matrix and pores but not the surfactants with branched alkyl groups.

### 6.2. Phonolics

Phenol and phenolic compounds are present in the effluents of various industries such as oil refining, petrochemicals, pharmaceuticals, coking operations, resin manufacturing, plastics, paint, pulp, paper, and wood products [270]. Phenol has been designated as a priority pollutant by the USEPA. Thus, there is an apparent need to treat wastewater containing phenolic compounds before discharge. With the growing interest in applying RO for industrial wastewater treatment, vigorous research for phenolic compound removal has been conducted [131]. During such works, appreciable fouling phenomena were observed for specific phenolics. Table 14 summarizes the research on various phenolics. 

When considering the fouling of phenolics, the effect of feed pH and concentration needs to be considered. For example, phenolics’ acid dissociation constant (pKa) varies greatly, as shown in Table 15. At neutral conditions, phenol exists as a non-dissociated form. Meanwhile, 2, 4-Dinitrophenol (DNP) exists as a dissociated form with a negative charge. With a feed pH higher than pKa, flow decline rates are decreased when RO/NF membranes have a negative charge. In the case of TCP, at acid conditions (pH 4–4.8), nearly a 50% flux drop was observed. However, at pH 8.6, the flux drop was 8.4% [132]. A similar behavior was observed in Nitrophenol (NP) [279]. When treating DNP under acidic conditions, i.e., pH 3–4, the extent of flux drop might be increased since the TFC RO/NF membranes are positively charged at this pH. 

When considering the fouling of phenolics, it might be appropriate to classify them as follows:

- Phenol, di, and tri-hydroxybenzene: Hydroquinone, Resorcinol, Catechol, Phloroglucinol, Pyrogallol;
- Mono-substituted phenols: 2AP, 2FP, 2CP, 2NP, etc.;
- Di- and Tri- substituted phenols: DCP, TCP, DNP;
- Others: BPA.

Different data exist regarding the fouling behavior of phenol. A flux reduction of more than 50% was found for the FT-30 RO membrane with 2.5 mmol/L (235 mg/L) and 15.2 mmol/L (1430 mg/L) of phenol [131]. Van der Bruggen et al. [121] observed 33% and 15% flux decline for NF70 and UTC-20 NF membranes with 10 mmol/L (940 mg/L) phenol. On the other hand, no flux decline was observed for 0.5 mM of phenol at pH 4.5–4.6 [178]. For NF90, TFC-HR, and BW30 membranes, 8%, 6%, and 6% of flux decline were reported, respectively, with 100 mg/L of phenol at pH 5.5 [276]. The effect of feed concentration can explain this phenomenon. With increasing phenol concentration, the flux decline was increased from 20% at 2.5 mmol/L to 40% at 20 mmol/L for the NF70 membrane [121]. The same behavior was observed for 4NP [279]. Thus, the concentration effect must be carefully considered when judging the fouling tendency of phenolic compounds.

Phenol, di, and tri-hydroxybenzenes have low fouling properties compared with chloro- or nitro-substituted phenolics due to their hydrophilic nature and less adsorption capability to RO/NF membranes [273,274,275,276].

Several researchers conducted a series of experiments using mono-substituted phenolics. The degree of flux decline was reported as follows:
AP > NP > CP > FP > PHE, (FT30-BW, pH4.5–4.6)[178]NP (Relative Flux 0.67) > 3NP, 2NP (0.74) > 4CP, 3CP (0.76) > 2CP (0.79) > PHE (0.94), (BW30, pH5.5)[275,276]2NP (0.83) > 2CP (0.92), (BW30)[277]4NP > 3NP > 2NP > PHE, (CPA2, pH7, 5 mg/L)[281]

These orders were well correlated with adsorption amounts to RO/NF membranes. Arsuaga et al. [275,276] correlate the solute adsorption amount (Q) and flux decline with the logarithm of the octanol-water partition coefficient (log K_ow_). Good correlations between Q and log K_ow_ and relative flux and log K_ow_ were observed. Thus, they pointed out that the extent of phenolic compounds’ adsorption should be promoted by hydrophobic interactions between them and the membrane structure.

As for tri-substituted phenols, TCP shows the most significant flux decline, where about a 40% decline was observed at pH 4.5 and 100 mg/L of TCP [132]. This result can be attributed to the large adsorption amount and log K_ow_. The adsorption trend was found to be consistent for FT30 and NF40 membranes: TCP > DCP > CP. Regarding the di-substituted phenols, DNP’s apparent deviation was observed at pH 3–4 [178]. DNP caused a very high flux drop, even though DNP had a similar adsorption amount of 2NP. It was suggested that the pKa for the phenolics is a good measure of the water flux decline phenomenon. Another possible explanation is the strong electrostatic interaction between the FT30 membrane and DNP, since at pH 3–4, the FT30 membrane is positively charged, and a part of DNP has a negative charge. At pH 7, the flux decline rate of NDP for the CPA2 membrane was similar to 4NP, 2CP, and 4CP [281]. 

Williams et al. [178] reported that the TCP-fouled membrane compositions (carbon, nitrogen, oxygen, sulfur, and chlorine) were analyzed by ESCA. In situ argon ion sputtering was used to etch away the membrane barrier layer to obtain compositions as a function of depth. A chlorine atom was detected in an entire PA active layer. These data may suggest that pore blocking might cause severe fouling of TCP trapped in a PA matrix or pores due to strong interactions. In contrast, negligible flux decline was observed for benzene due to its lack of hydrogen-bonding capability, although benzene had a high physical adsorption. 

The effect of membrane type, APA vs. PPA, and LP vs. ULP shows the same behavior observed for surfactants. The CA membrane showed less flux drop for phenol at high concentrations [131]. The flux drop of the FT30 is more significant than that for the piperazine type of NF40 with 50 mg/L TCP, even though adsorption amounts are similar [271]. The NF70 (APA) showed a higher flux decline than UTC-20 with 10 mmol/L phenol [121]. Regarding LP vs. ULP, ULP type of NF90 showed a more considerable flux decline than TFC-HR for 100 mg/L of phenolics [274] and BW30 for 100 mg/L of 2CP and 2NP [277]. However, it should be noted that there is a report that no prominent difference was observed among NF90, TFC-HR, and BW30 for various phenolic compounds [276].

As mentioned, leached BPA from the RO/NF permeate spacer was detected from fouled RO/NF membranes. This topic is discussed in more detail in the subsequent section. Although it is known that trace amounts of BPA have an adverse effect on RO/NF membranes, detailed studies about organic fouling are limited. Escalona et al. [278] investigated BPA degradation during Fenton’s process under different operational conditions and a process combined with subsequent NF to treat low-concentration remnant BPA and compounds derived from oxidation. The study observed significant fouling for BPA and the effluent after Fenton oxidation for five commercial NF membranes. Similar to phenolic compounds, BPA also showed intense concentration-dependent fouling behavior. After 200 min of filtration, the normalized flux decreased from 58.3 to 23.5% when the BPA feed concentration increased from 25 to 300 mg/L.

Regarding the effect of membrane type, all the membranes, including the CA membrane, showed apparent flow loss for 300 mg/L of BPA. The normalized flux dropped by between 47 and 77% within the first minutes after the filtration started, and then the normalized flux was stabilized. The normalized flux for ESNA1-LF2 (ESNA) presented the slowest decline, followed by NF90, NFD, NF270, and CK (CA). The pure water flow recovered remarkably after flushing the fouled membranes with deionized water. However, depending on the type of membrane, its recovery rate varies. Irreversible membrane fouling increased in the following order: NFD < NF270 < CK < NF90 < ESNA. In this research, the Fenton oxidation was employed to eliminate BPA. NF filtration was applied for the Fenton-treated effluent containing 571 ± 50 mg/L of COD, 222 ± 20 mg/L of TOC, and pH = 2.71± 0.04. Although the Fenton process virtually eliminated BPA, apparent flow loss was observed for four TFC NF membranes. The CK membrane featured the lowest permeate flux decay (ca. 20%), followed by NFD, NF270, and ESNA with similar flux decays (ca. 55–60%), and finally by NF90 with the highest flux decline (ca. 75%). The fouling seemed irreversible because deionized water flushing was insufficient to restore permeate flow.

In the case of TCP degradation by ozone treatment (a 1.8 L stirred reactor with a flow of 0.20 standard liters per minute O_2_ containing 2% ozone), after 5 min of ozonation, the TCP concentration was reduced from 50 mg/L to 24.7 mg/L, and after 15 min, was <0.5 mg/L [271]. Flux drop for the FT30 membrane was notably reduced after 15 min of ozonation. These results might indicate that an advanced oxidation process (AOP) should be carefully selected to eliminate phenolic compounds when combined with RO/NF membrane processes.

### 6.3. Tannins and Tannic Acid

Sometimes, both terms (tannic acid and HA) tend to be used interchangeably regarding organic color in natural waters. Tannic acids are typically low molecular weight phenolic/carboxylic compounds obtained as soluble extracts from certain plants’ bark or fruit, such as oak or sumac. In a previous instance, when dealing with highly colored well water, laboratory studies were conducted at a membrane manufacturer to characterize the organics. FTIR spectra did not show sufficient differences. At the subsequent examination with UV, the well water sample exhibited a UV absorption maximum at 270 nm, which matched that of the HA but not with the tannic acid spectrum [163]. 

RO/NF, separation of tannins and tannic acid, involves the following three areas:

- Wastewater treatment in the tanning (vegetable tannins) industry;
- Investigation as membrane performance enhancers and rejuvenation agents;
- Use as a model for NOM or DBP precursors.

Vegetable tannins are polyphenols with molecular weights from 500 to 20,000 that are used in the tanning process to convert hides and skins to leather and as retanning agents [283]. An NF process was investigated to recover tannins and water from exhausted baths and to reuse them as tanning agents and washings [283,284,285]. Significant fouling was observed for various NF membranes, although the extent of flow loss varies among membranes. However, it is challenging to elucidate the effect of tannin itself since the feed water contains many organic and inorganic impurities.

Gallotannins are formed when gallic acid units are added to the galloyl (3,4,5-trihydroxyphenyl) groups. This type of hydrolyzable tannin is commonly referred to as tannic acid [286]. Although tannic acid is considered a foulant to RO/NF membranes, tannic acid has been used to enhance RO/NF membrane performance, especially the PA hollow fiber RO [287]. The B-9 and B-10 “Permasep” permeator was post-treated with PT-A (polyvinyl methyl ether) during manufacturing. B-10 permeators are also post-treated with PT-B (tannic acid). PT-A increases salt rejection by reducing salt passage through membrane or fiber imperfections. New B-10 permeators usually had to be treated with PT-B before placing them on-stream and after any cleaning operation [288].

Tannic acid has been examined to enhance TFC RO/NF membrane performance, that is, salt rejection, chlorine tolerance, etc. Sato and Tamura [289] investigated the effect of tannins and tannic acids (hydrolyzable, condensed tannins, deviation from tannin, and synthetic tannins) on separation performance and flux in NF membranes, LES90 (APA TFC). It was found that Chinese gallnut tannic acid had the highest effect on improving salt rejections. Meanwhile, the flow decline was limited and less than 10% for all tannins evaluated. Since LES90 is a tight NF that may have a similar pore structure to RO, the permeation or plugging of tannic acid might be limited due to a higher MW of 1700 for Chinese gallnut tannic acid. Phadunghus et al. [290] compared NF and RO membranes with 70% and 90% salt rejection under operating pressure 0.4 MPa and NaCl concentration 0.01 M. A severe decline in the flux of the NF membrane was observed for 10 mg/L of tannin compared to RO. This result was attributed to pore blocking by tannic molecules on the membrane surface.

Three commercial NF membranes (NF40, ROM 378, DRA 4020) were examined for removing chlorinated organic compounds from the first alkaline extraction effluent originating from a kraft mill [272]. Several reference solutes, including saccharides, TCP, and tannic acid, were also examined during the tests. The production rates of 2000 ppm of tannic acid are significantly lower than those of saccharides (2000 ppm) and TCP (100 ppm). 

Tannic acid has been used as a model foulant for NOM and DBP precursors. Tu et al. [291] conducted NF tests to conceptualize and develop a membrane transport model for removing natural organics and tannic acid, a NOM model compound. The tests showed that the NF-45 membrane composed of PPA was more susceptible to organic fouling by tannic acid than the NF-70 membrane made of cross-linked APA. 

The removal of DBP precursors in the presence of calcium was investigated using NF70 membrane, with resorcinol, phloroglucinol, 3-hydroxybenzoic acid, and tannic acid serving as model compounds [273]. The relative flux drop with 5 mg/L tannic acid was more than 20% at pH 7, which is higher than for low MW model compounds. The interactions of model compounds with calcium have no significant effect on flux decline. The flux increases with pH, which is consistent with the behaviors of phenolics. A comparison between membrane types NF70 (APA) and NF270 (PPA) revealed contrasting results [292]. Unlike the previous results, a more significant flux drop was observed in NF70 compared to NF270. Although there seems to be some discrepancy, medium-sized tannic acid had more profound fouling effects on NF membranes than HA.

Tannic acid is also a model foulant when developing low-fouling RO membranes. The TFC RO membranes modified by grafting hydrophilic PEG were evaluated with 100 ppm of DTAB and tannic acid [233]. Although the surface-modified membrane showed less fouling propensity, both unmodified and modified membranes showed significant flux declines (>50%) with DTAB and tannic acid. Since a laboratory prepared the TFC RO membranes, its pore structure and salt rejection characteristics are unclear. Zhao et al. [245] reported on the PEG-coated fouling-resistant membranes. Tannic acid (50 ppm) fouling was much milder than DTAB (25 ppm), even for unmodified membranes. 

### 6.4. Dyestuffs

The textile industry generates large volumes of effluent. Membrane filtration has become an essential part of advanced treatment plants for dye wastewater treatment [293]. Since the wastewater contains many organic pollutants, including surfactants, dyes, EfOMs, etc., organic fouling is of critical concern [254]. However, assessing the effect of dyestuffs themselves is challenging due to the existence of mixed foulants, especially surfactants. Thus, various dyestuff model compounds have been examined to identify the fouling mechanisms.

Since dyestuffs usually have larger MW than MWCO of RO membranes and bulky structures, fouling can be considered to occur mainly on the RO membrane surface, i.e., surface fouling. In the case of NF, when dye molecules are equal to or smaller than MWCO, sorption to internal pores might partially be attributed to a cause of fouling. Table 16 summarizes the research on various dyestuffs.

Since dyestuffs are typically charged (ionized), an electrostatic interaction is a contributing factor to RO/NF fouling. Thus, dyestuffs show fouling behavior similar to that of ionic surfactants. For example, acid dyes do not show notable flux decline in TFC RO/NF membranes at neutral pH because both dyes and membranes are negatively charged [253,294,295,296,297,298]. Chidambaram et al. [299] investigated the effect of the molecular charge of different dyes (three anionic and three cationic dyestuffs) and the pH of salt solution on NF water flux. Dye molecules of 50 mg/L dissolved in 2000 mg/L of NaCl were filtered using the NF270 membrane (isoelectric point of 3.3) at three different pHs (3, 7, 10). Acid dyes maintained their flux at three pH. However, the dye having weak carboxylic acid (Acid Red 87) showed substantial flux decline and membrane fouling in acidic pH. This flow loss is attributed to hydrophobic interaction at pH 3, i.e., no charge in membrane and dye. Positively charged dye molecules with relatively low molecular weights exhibit strong fouling effects in neutral and alkaline pH. 

The effects of salt and dyestuff concentration must be considered when treating dye bath wastewater. A flux decrease is observed for high concentrations of dye solutions of 1 g/L (acid, basic, and disperse dyes) at pH 6 [300]. However, after rinsing with water, the water permeability was increased and higher than the initial value for the anionic dyes (Direct Red 80 and Acid Red 4). As for the basic and disperse dyes, flux was not fully restored. In the following case, the lowest flux values were obtained at high pH in testing the Reactive black 5 and NaCl concentrations of 9 and 80 g/L [301]. This phenomenon was attributed to the formation of a strong and stable dye-salt complex that increases dye hydrophobicity [301,302].

**Table 16:** Summary of RO/NF research for treatment of dye compounds.

Dyestuffs: Acid Dye	Basic Dye	Others	NF	RO	Reference
Reactive Blue 4, Reactive Red 2				PA	[253]
Reactive Red (Benefix)			NTR-729HF LES90	ES20	[303]
Reactive Orange 16, Reactive Blue 2			UTC-60, NF70, NTR- 7450		[294]
Acid Red 4, Acid Orange 10, Direct Yellow 8, Direct Red 80, Reactive Orange 16	Basic Blue 3	Disperse Blue 56	5DK		[300]
Reactive Black 5			DS5 DK		[301]
	Crystal Violet		TFC NF		[304]
Cibacron red P-B, Cibacron red LS-B			MPF-34 MPF-36		[305]
Acid Red 4, Acid Orange 10, Acid Red 27			DK		[295]
Acid Orange 7			NF45		[296]
	Crystal Violet		NF (400 MWCO)		[306]
Marine E-EL				ES20, LF10	[254]
Tartrazine (FD&C Yellow 5)			FM NP1010		[297]
Marine E-EL				ES20	[255]
Reactive Black 5			PPA NF, CA		[307]
	Methylene Blue Rhodamine B			PROC10, CPA2, ESPA2	[308]
Everzol Black, Everzol Blue, Everzol Red			NF200, NF270		[298]
EV, Amido Black 10V, Eozine Yellow	Azure A		NF270		[309]
Acid Red 87, Direct Blue 53, Acid Black 1,	Azure A, Basic Blue 9, Basic Green 4		NF270		[299]
Reactive Black 5				TFN, XLE	[310]

### 6.5. Aromatic Compounds

Among the LMWOC foulants, aromatic compounds are recognized as strong foulants due to hydrophobic interactions between membranes and foulants. Here, additional aromatic compounds are indicated as examples of foulants.

In the previous section, alkylated diphenylamine compounds cause a decrease in flow rate. Similar behavior was observed for diphenylamine [311]. The RO membrane surface was modified with diphenylamine to improve the chlorine and fouling resistance. The membrane surface was contacted with diphenylamine after sodium dioxide treatment. Significant flow reduction was observed for post-treated RO membranes.

Li et al. [281,282] investigated the influence of various interface forces on membrane fouling using monoaromatic compounds as model foulants. Among them, aniline, 4-nitroaniline, and substituted phenolics induced significant flow decline. On the other hand, the flow decrease for phenol, hydroquinone, and benzoic acid was minor. It was mentioned that the initial fouling rate induced by the adsorption of monoaromatic compounds on the RO membrane was dominated by electrostatic attraction and hydrophobic force.

In petrochemical wastewater treatment, organic fouling is a critical concern. Li et al. [312] studied the fouling behavior of RO fed with UF-treated petrochemical secondary effluent. With the micro-analysis of the fouling layer, the residual organic foulants were the primarily responsible elements for the evolution of irreversible fouling. The aromatics, including phenol, methylphenol, benzeneacetic acid, dibutyl phthalate, and xylenol, were proposed to be the predominant organic foulants causing irreversible fouling. Alkaline solution (NaOH, pH 11.0) exhibited the best cleaning performance in permeability recovery.

The coupling of membrane separation and photocatalytic oxidation was studied to remove pharmaceutical pollutants [313]. As a part of the studies, six pharmaceuticals, including five aromatic compounds, 10 mg/L of each compound with a pH of ca. 7, were filtered with RO and NF membranes. Significant flux decline was observed during the test run for the NF90 membrane. The flux dropped steeply, around 20% at the initial stage (first 12 h), then slowly over the subsequent 12 h period, exhibiting a permeate flux decline of 5%.

Vanillin and cinnamaldehyde are known as quorum sensing inhibiting compounds that disrupt quorum sensing pathways and ultimately reduce biofilm production [314]. To improve the anti-biofouling characteristics of RO membranes, vanillin (MW: 152) and cinnamaldehyde (MW: 132) were physically deposited onto commercial TFC RO membranes (SWC5 and SW30XLE) by recirculating 1200 mg/L solution without permeation. Afterward, the membrane was washed with fresh deionized water for 15 min. There were no statistically significant differences in pure water permeate fluxes, although the average water permeate fluxes were slightly lower for modified membranes. However, modified membranes showed an apparent flow decline during biofouling experiments using artificial seawater. Modified SW30XLE membranes had a 40% lower starting permeate flux than virgin SW30XLE membranes [315].

### 6.6. Oil and Grease

Oil and grease (O&G) are recognized as harmful substances to RO/NF membranes. O&G is any material recoverable as a substance soluble in a particular solvent (Standard Methods for the Examination of Water and Wastewater, 23rd.) [316]. In this analytical determination, groups of substances with similar physical characteristics are quantified instead of an absolute quantity of a specific substance. USEPA uses the term “n-Hexane Extractable Material (HEM; Oil and Grease)” for the material that is extracted from a sample using the extraction solvent n-hexane [317,318]. O&G substances can be divided into two main classes, differing in origin and chemical constitution. The first category has mineral provenance, namely from petroleum and its derivatives, and consists of a mixture of hydrocarbons of different chemical configurations; a second group is of biological origin (animal or vegetable) and is mainly composed of triglycerides, i.e., esters of glycerin and fatty acids [319]. Thus, in the food industry, fatty acids are a major effluent component of O&G. Hydrocarbon is a critical component of effluent in the refinery and petrochemical industry.

In a particular instance, to address a concern about the effect of oil spills, key RO manufacturers were contacted to obtain information on their tolerable O&G levels [320]. One supplier responded that their membranes could be operated with 0.3 ppm of oil. The other manufacturer stated that in a “produced” water system, exposure of the membranes to an oil level of 5 ppm or greater resulted in rapid membrane fouling. Mussalli et al. [321] reported that a survey of membrane manufacturers revealed operational limits of 0.3 ppm of oil. As for TFC RO, Redondo and Lomax [322] mentioned that coagulation or AC must be applied when oils contaminate the RO feed water at levels above 0.1 mg/L. These substances are readily adsorbed onto the membrane surface. Thus, membrane manufacturers recommend <0.1 mg/L O&G in the RO feedwater [96,323,324]. Furthermore, Voutchkov [325] suggests that O&G content in the source water be maintained below 0.02 mg/L at all times. 

O&G fouling is of great concern in the following events and applications:

- Seawater contamination and oil spillage (hydrocarbon, crude oil, etc.);
- Petrochemical and oil refinery wastewater and produced water treatment;
- Food and beverage industry: vegetable oil production and wastewater treatment;
- Leakage of hydraulic fluid and pump sealing oil.

Oil spillage or contamination could prevent achieving steady and reliable operation in desalination plants. In severe cases, RO plants had to be shut down for a long period [326,327]. The problem of oily water contamination in seawater is of great interest in the Gulf area [328]. This concern significantly increased when a large amount of oil spilled in 1991, resulting from the Gulf War. Soon after the Gulf War ended, Goto investigated the effects of oil spills on seawater desalination plants in that area [329]. He reported that it is necessary to remove the oil through pretreatment due to membrane sensitivity to the oil. Since there was little information on oil removal at the time, he stressed the necessity of clarifying the pretreatment conditions and oil removal rates for various oils (dispersed and soluble oils) and further proposed examining UF technology. Kiefer and Mussalli [320] mentioned that possible treatment alternatives are dissolved air flotation (DAF), special filter media for oil separation, and NF/UF. 

It is reported that nearly all the oil (10–30 ppm of Saudi diesel oil) with both its aliphatic and aromatic components are removed by the coagulation (FeCl_3_)-filtration process, with only traces of aromatic (possibly water soluble) of less than 0.8 ppm [330]. Usually, oil does not dissolve in water. However, due to weathering like high temperature, sunlight, waves, etc., the oil dissolved and became emulsified, making it difficult to separate from water with regular oil interceptors or settling procedures [328]. It was found that weathered oil-contaminated seawater (WOCS) contained soluble components with relatively small molecular size, which are refractory to biodegradation and difficult to remove by AOPs, UF membrane, or coagulation using FeCl_3_ or polyaluminum chloride [331]. However, DOC in the WOCS was readily adsorbed to GAC.

Moreover, since the solubility of hydrocarbons in seawater is approximately 30–40% less than that in distilled water [332], it might be important to remove dissolved oils prior to RO. There are large-scale plants treating raw seawater with GAC. The Ras Abu Jar Jur plant in Bahrain was designed to operate with oil levels of up to 10 ppm in the feed [320]. However, the oil levels at this plant site have not reached 10 ppm, so there are no actual GAC operating data for these oil levels. Jian et al. [331] reported that NF membranes with higher salt rejection rates could remove more DOC from the WOCS than those with lower salt rejection NF membranes. Hence, NF systems may be used before regular RO systems as pretreatment to remove oil. However, the effect of fresh and weathered oils on long-term NF membrane performance is unknown.

Since oil composition and concentration during oil spill incidents are unpredictable and highly variable, a more robust and comprehensive pretreatment system is required. To address such risks for severe oil spills, Ogunbiyi et al. [333] recommend an integrated approach consisting of offshore cleanup, seawater intake (e.g., subsurface intake), and robust onshore pretreatment.

For petrochemical and oil refinery wastewater and produced water treatment, a high oil concentration has been a critical issue when applied to RO processes. The oil concentration of the produced water in the San Ardo oil field was reported to be 80 mg/L [334]. At a pH of 6 and 7.5, oil solubility was 50 mg/L and 225 mg/L, respectively. In addition, it was challenging to treat the produced water by RO due to the very high silica and boron concentrations, 250 mg/L and 20–30 mg/L of each. To address difficulties in scaling and fouling, a unique high pH (pH 10.6–11) RO process was examined after the feed hardness was completely removed, where the oil solubility was increased to about 350 mg/L. Other researchers have also reported the high pH process to treat the produced water [335]. Loganathan et al. [336] assessed two distinct treatment processes to evaluate the impacts of pretreatments on the RO performance of pretreated recycled water from an oil sands facility. The first process consisted of coagulant addition, ceramic UF, and antiscalant operated at natural pH. In contrast, the second process included softening, coagulant addition, ceramic UF, weak acid cation ion exchange, and antiscalant addition operated at alkaline pH. During the pilot tests, CIP was not required for both treatment configurations, although the feed O&G concentrations were 25–27 mg/L.

Two UF membranes were tested as RO pretreatment to remove oils from the produced water [337]. In the case of UF with MWCO of 3500, the RO flux reduction was less than that of hollow fiber UF with MWCO of 50,000. However, after a few days of operation, the RO system flux declined significantly in both cases, indicating irreversible membrane fouling. It was speculated that the irreversible fouling was caused by the oil molecules and other organic compounds in the produced water. 

Although rigorous O&G removal before RO is also requested in petroleum industry wastewater treatment, there is a report that the system could operate without causing a prominent flow decline despite high oil concentrations [338]. The stripped sour water within refineries containing rich phenolic content was evaluated without pretreatment. Three different wastewater samples were processed over a representative operational period; the two samples experienced no flux decline in RO with O&G of 4 and 98 mg/L. Flux decline due to hydrocarbon and phenolic adsorption was observed where O&G content in the feed was 771 mg/L.

As mentioned, fatty acids are a major effluent component of O&G in the food industry. For example, wastewater from the vegetable oil industry contains high levels of O&G, such as triacylglycerol and fatty acids [339]. Thus, rigorous pretreatment was applied to treat oily wastewater in an edible oil production facility [340].

Leakage of hydraulic fluid and pump sealing oil to RO feed water has been occasionally reported [341]. A unique triple-pass pilot plant was designed and constructed to demonstrate the feasibility of meeting U.S. Navy shipboard high-purity water needs by employing RO desalination. However, oil leaking from the first-pass pump before the 90-day endurance test fouled the first-pass elements to the extent that standard cleaning solutions could not restore the elements to satisfactory performance levels. This contamination did not affect the second- or third-pass elements.

#### 6.6.1. Hydrocarbons

Residual hydrocarbons have been recognized as harmful in RO operations. Thus, the hydrocarbons must be strictly removed in pretreatment. However, contrary results have been reported. For example, aromatics such as benzene, naphthalene, anthracene, and phenanthrene showed little drop in permeate water flux [178,271]. Although benzene had a high physical adsorption, a negligible flux drop was seen. Piekutin [342] evaluated the effect of hydrocarbons where gasoline and diesel fuel, mixed in a 1:3 ratio, were added to surface water. Three samples with varying benzene, ethylbenzene, toluene, and xylene (BTEX) concentrations, 0.8, 9.5, and 15.8 mg/L, were tested. At 1.0 MPa, a slight flow decline of 10–15% was observed compared with the surface water for all three concentrations. However, at 1.2 MPa, significant flow loss was observed for the sample of 15.8 mg/L of BTEX. Nordham [343] examined the capability of the RO system pretreatment filters to remove the light crude oil contaminant from seawater feed streams. It was found that the tested polypropylene and polyolefin cartridge filters would remove almost all of the crude oil from in the range of 172–469 ppm. The treated feed water with residual 1–4 ppm of oil was supplied to the RO system. The change in normalized permeate rates from baseline conditions was small and considered insignificant. The results from this test indicated that the short-term performance of an RO element with a few ppm of oil did not appear to be significantly affected by the oil-contaminated feedwater. Another test was conducted by using synthetic emulsified Iranian crude oil [344]. At the 0.3 vol% of oil, no significant flow loss was observed for the FT30 RO membrane.

The effect of a range of contaminants on RO membrane performance was assessed before and after exposing the RO membranes to the oil-based media [345]. The exposing solutions comprised crude oil, crude oil/water mixtures, diesel, diesel/water mixtures, hexane, and hexane/water mixtures. When they are in an emulsion form or pure hydrocarbons, these cause extremely severe reductions of RO membrane flux, as opposed to when they are in solution in the aqueous phase. Further, prominent differences were seen between crude oil and diesel oil. A short (three hours) immersion in a single-phase crude oil, oil/water solution caused a subsequent 2% decrease in permeate flux and +3% change in salt passage. A six-day exposure to emulsified crude oil caused no measurable effect on the permeate flux. Meanwhile, diesel oil considerably reduces permeate flux for both BW30 and SW30 membranes after only 30 min of exposure to fairly dilute diesel/water mixtures of 1/50 or 1/100 ratios. The flux was reduced to zero when the BW30 membrane was exposed to 1/10 mixture for 30 min.

In the case of hexane/water mixtures, When the BW30 membrane was contacted with water phase (without stirring) in a 1:50 hexane/water mixture, there was virtually no change in flux. When the BW30 membranes were immersed in a stirred fluid (two phases), they suffered damage, which caused a substantial reduction in flux. As for the dissolved hexane, no discernible changes in flux or salt passage were apparent after immersion in the solution without stirring for 1, 3, and 15 h. Owadally [346] refined the experimental conditions by contacting water/oil mixtures either to both sides of the membrane or the active layer only. It was found that the most harm was done to the polysulfone (PSF) interlayer; microscopically, it was observed that the pores of the layer were fused, causing a complete blockage of the membrane. Thus, if the pre-treatment system effectively removes the insoluble hydrocarbons, the residual O&G contaminants, provided they remain in solution, may not seriously threaten the RO membranes.

**Figure 10:**
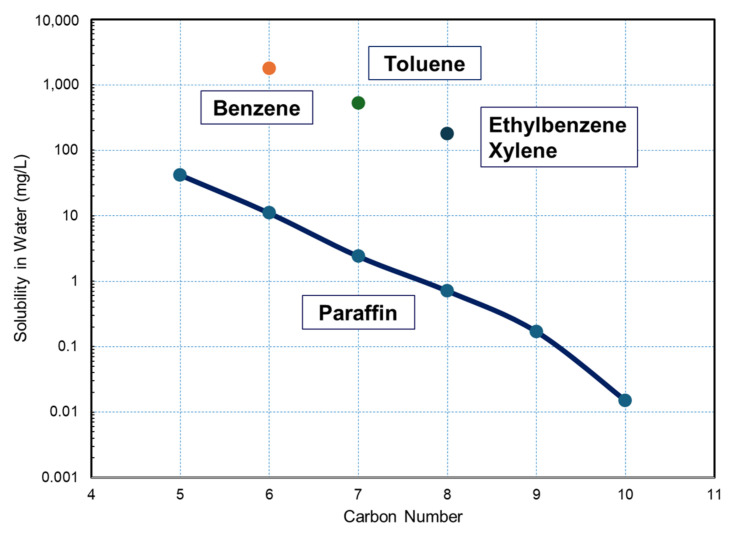
Paraffin and BTEX solubility in water [347,348].

According to the reports, hydrocarbon solubility in water could explain the fouling phenomena of hydrocarbons. As shown in Figure 10, the solubility of aromatic hydrocarbons such as BTEX is much higher than that of paraffins. 

It is reported that diesel oil consists mainly of aliphatic hydrocarbons of C9 to C26 with some traces of aromatic hydrocarbons [330]. In addition, hydrocarbon solubility is decreased with increasing salinity [332,349]. Thus, the solubility characteristics could explain the different fouling behavior between diesel oil and gasoline (or BTEX). Caution may be required when treating hydrocarbons, especially paraffins with a carbon number of more than seven (7), as a free oil layer may be formed during RO operation (concentration polarization and feed concentration).

#### 6.6.2. Fatty Acids

When treating effluents from food and dairy industries such as vegetable oil processing, membrane fouling with O&G and higher fatty acids should be of great concern. O&G is the third most abundant component of biologically treated sewage effluent [116]. However, fatty acid fouling may not be critical in treating municipal wastewater by RO since almost all fatty acids are eliminated during biological treatment [116,350].

Since the higher fatty acids consist of a long aliphatic hydrocarbon chain and a hydrophilic carboxyl group at one end, the fouling tendency is similar to surfactants. When feed pH is higher than pKa, the fatty acids behave like anionic surfactants (soaps). Meanwhile, the fatty acids have identical fouling characteristics for nonionic surfactants at lower pH (<pKa). Ang and Elimelech [118] investigated the effect of feed solution pH and Ca^2+^ concentration on the fouling of RO membranes by octanoic acid, selected to represent fatty acids in EfOM. RO membrane fouling is more significant at a solution pH below the pKa of the octanoic acid (pKa = 4.9) than at an elevated pH. The normalized flux at pH 4 was instantly decreased by 40% after contacting 2 mM (288 mg/L) of octanoic acid under the initial flux of 83 LMH. At a higher pH of 9.0, the permeate flow was gradually decreased and stabilized at 85% of the normalized flux. Octanoic acid permeates more readily at solution pH below its pKa than at elevated pH. Thus, these fouling and transport phenomena can be explained by the electrostatic interactions at higher pH and the attractive hydrophobic interactions at lower pH. 

At pH below the octanoic acid pKa, fouling behavior is unaffected by calcium ions, whereas at elevated pH, the flux decline rate decreases with higher calcium ion concentration. When the light scattering measurements were performed in various Ca^2+^ concentrations at low pH and elevated solution pH, the scattered light intensities increased instantly in the presence of Ca^2+^ at elevated solution pH. The increased scattered light intensities imply that the octanoic acid molecules aggregate in the presence of Ca^2+^ (calcium soap). This phenomenon was explained by the formation of octanoic acid-calcium complexes as micelle-like structures with hydrophilic ends containing calcium and face bulk water. Thus, it might be considered that the fouling mechanism is changed from octanoic acid sorption to colloidal fouling by adding calcium ions.

Li et al. [119] conducted the investigations with additional factors, i.e., effects of octanoic acid concentration (0.21–3.0 mM), bulk ionic strength, and temperature. Furthermore, attenuated total reflection Fourier transform infrared (ATR-FTIR) spectroscopy was utilized to semi-quantitatively determine the adsorption of the foulants on the membrane surface. The flow decline rates were well correlated with the adsorption amount, as seen in peaks of carbonyl groups of FTIR. Regarding the effect of the ionic strength, the flux decline is insignificant when the ionic strength is 1.0 mM. There is a significant change in the flux profiles when the ionic strength is increased to 10 and 20 mM. These changes were explained by the shielding effect generated by the counterions. However, the fouling tended to decrease at an ionic strength of 50 mM. This phenomenon was attributed to micelle formation at a higher ionic strength of 50 mM. Lower temperature was found to increase the rate of octanoic acid fouling, which was attributed to changes in membrane permeability and mass transfer.

Ang et al. [351] compared the fouling of RO membranes with typical organic foulants. The model organic foulants chosen to represent the polysaccharides, proteins, HA, and fatty acids were sodium alginate, Suwannee River NOM, BSA, and octanoic acid. The extent of flow decline was insignificant, about 10%, at 25 mg/L concentration, under the initial flux of 83 LMH and pH of 6, and was approximately the same for all four types of foulant. Puro et al. [352] tested three types of NF membranes with a high octanoic acid concentration of 500 mg/L. Although a slight flux decline was observed, pure water flux was restored after flushing with pure water. Kim et al. [353] reported the case of linoleic acid. RO experiments were conducted at 15 bar with 1, 10, and 100 μM of linoleic acid. 1 μM of linoleic acid decreased the flux rate by less than 10%. On the other hand, dosing with 10 and 100 μM (28 mg/L) concentrations resulted in a significant flux decline. Thus, solubility may be a limiting factor for higher fatty acid fouling.

In summary, since O&G can be considered an ill-defined parameter to express fouling tendency, it may not be suitable for defining RO/NF feed water quality requirements. For example, fouling by hexane (C6) and octanoic acid (C8) is insignificant, with lower feed concentrations under regular RO operating conditions (pH, flux, temperature, etc.).

### 6.7. Leachables from RO/NF Elements and Plant Components

As mentioned in Section 5, leachables from RO/NF plant system components and construction materials could cause significant flow rate reduction [139]. Such components are pipes, FRP tanks, concrete pits, ion exchange resins, cartridge filters, etc. Some case studies have already been published, including leachables from RO elements (phthalate esters), a reinforced polyester pipe, and an NF permeate spacer. Regarding the cartridge filters, it is said that some types of string-wound cartridge filters comprise about 1 wt% of chemicals consisting of lubricants, surfactants, antistatic agents, antioxidants, bactericides, emulsifiers, etc. [354]. During the start-up of new cartridge filters, the leached residual chemicals enter the RO system and cause performance problems (low flow due to fouling) [355]. In some cases, the fouling is irreversible and cannot be removed by regular cleaning. In the case of polypropylene depth filters, phthalates are used in their manufacture. As little as 50 ppb of phthalate could irreversibly foul an RO membrane [356]. The author has experienced that leachables from epoxy linings applied to storage tanks, water supply pipes, etc., could harm RO membranes. 

What makes this fouling phenomenon different from other types of organic foulants is that a sudden flow loss is unexpected. Although some flow reduction due to organic fouling can be expected in municipal and industrial wastewater treatment systems, this fouling is often observed soon after an operation starts, which is troublesome for plant operators and membrane suppliers. This subsection covers the following four topics: plasticizers, leachables from epoxy, spiral RO/NF elements, and ion exchange resins.

#### 6.7.1. Plasticizers

Plasticizers are additives in the plastic industry that increase the durability and flexibility of plastics [357]. Diesters of phthalic acids, known as phthalates, have been commonly used as plasticizers. Thus, fouling with leached phthalates has been occasionally encountered, originating from pipes, cartridge filters, RO/NF elements, etc. [144,145,355]. Even if no acute problems occur, analysis of fouled membranes operating for a certain period may detect phthalates [358]. It is known that conventional high and low-pH cleanings were unable to restore the flux [144], and extreme cleaning methods are required that may restore the permeate flow partially [355]. One membrane supplier reported that cleaning with methanol restored flux to the original design specifications. The other supplier mentioned that flux was increased by 50% to 60% after cleaning with a high-pH caustic solution, followed by a 25% nitric acid solution. This CIP method might be based on their experience using a high concentration of nitric acid [100].

Three types of membranes, RO, NF, and UF, have been examined for phthalates removal from tap water [359]. The following mixed phthalates were used for the experiments: diethyl phthalate (DEP), DnBP, and di-2-ethylhexyl phthalate (DEHP). Three membranes showed apparent flux declines at 1.4 mg/L of mixed phthalates. The relative flux of RO, NF, and UF was decreased to 0.76, 0.66, and 0.81, respectively. The NF membrane showed the most significant decline in flux. 

Hasson et al. [145] conducted lab tests using flat sheet membranes to elucidate the effect of DnBP. First, 1 ppm of DnBP was added to the NaCl solution, which resulted in an immediate flux decline. During a period of 17 h, the permeate flow rate fell by over 50%. After that, 0.5% and 1% NaOH cleaning were examined. The cleaning operation restored the permeate flow rate to 70% of its original value. Then, the 1 ppm DnBP mixture was again circulated for 24 h. No further changes in permeate flow rate and salt rejection were noted. Next, the DnBP concentration was increased to 5 ppm. This increment caused a rapid increase in permeate flux accompanied by a corresponding increase in salt passage. It was considered that a high level of DnBP plasticizes the PA and lowers the glass transition temperature, resulting in increased mobility of polymer segments and increased flow rate. These behaviors resemble interactions between polyvinyl chloride (PVC) and plasticizers: antiplasticization at a low plasticizer concentration and plasticization at higher concentrations [360]. Jackson and Caldwell [361] observed that the modulus and tensile strength of the films are increased, and the elongation is decreased by incorporating certain additives in bisphenol A polycarbonate. This effect is called antiplasticization because the opposite results are obtained on plasticization—decreased modulus and tensile strength and increased elongation. Kinjo and Nakagawa [360,362] observed the same phenomena for slightly plasticized PVC with various plasticizers, including phthalates. The Young’s modulus vs. plasticizer content curve exhibits a maximum, which has been interpreted as an example of antiplasticization. However, the underlying mechanisms and implications of these observations remain unclear. Additional fundamental research is warranted.

To evaluate RO and NF membrane interactions with phthalates, static adsorption experiments were performed at different pH and ionic strengths [363]. The results showed that low MW dimethyl phthalate (DMP) and high MW DEHP strongly adsorb on the membrane compared with DEP and DnBP. Thus, a V-shape adsorption curve was observed when plotting against MW. This result demonstrates the co-existence of hydrophobic force and size exclusion in the adsorption of phthalates. Both pH and ionic strength demonstrated significant effects on adsorption. All the membranes showed a clear trend of increase in contact angle values after adsorption. Cheng et al. [364] investigated the adsorption of four kinds of phthalates: DEP, DnBP, dicyclohexyl phthalate (DCHP), and DEHP on NF membranes (NF90 and NF70). The order of adsorption amount was DCHP > DEHP > DnBP > DEP due to their hydrophobicity, molecular weight, and water solubility. This adsorption order is consistent with the previous study when eliminating the DMP result. It was also found that the presence of HA and Ca^2+^ influences phthalate adsorption on the NF membranes. 

However, in these reports, the adsorption amounts were not correlated with membrane fouling characteristics. Further investigation is necessary to clarify the effect of the phthalate adsorption amounts on flow losses and salt rejection.

TBP is a plasticizer for nitrocellulose, CA, etc., and is used to extract uranium and plutonium from other radionuclides in nuclear fuel reprocessing. When an effluent treatment facility at the Savannah River plant evaluated RO technology to treat low-level radioactive wastewater containing TBP, the fouling effect of TBP on RO performance was investigated [135]. RO tests with SW30 membrane revealed that 50 ppm TBP results in a 10% loss in the RO’s standardized permeate flow within 5 h and a 46% loss within 51 h. Higher feed TBP concentrations resulted in faster drops in RO performance. The RO tests demonstrated that RO fouling is reversible, and caustic detergent cleaning restored the permeate flow to 100%. In addition, filtered water circulation was effective for less severely TBP-fouled membranes to restore the flow rates. The effect of feed pH was seen in the subsequent report [365]. The water flux at pH 11 was higher than that obtained at pH 3. The stabilized dimensionless flux ratios were 0.6–0.65 at pH 3 and 0.85 at pH 11 for 50 mg/L of TBP. This test restored the water flux to prefouled levels by chemically cleaning the membrane with a pH 1.8 of oxalic acid solution.

#### 6.7.2. Leachables from Epoxy

Fouling caused by leachables from epoxy is a problem mainly in the following two cases: leachables from the permeate spacer of spiral-wound elements [148] and tanks and pipes applying epoxy lining. The primary leachable substance from epoxy is BPA. Since fouling phenomena caused by BPA have already been mentioned, supplemental explanations are provided here.

BPA can be considered as a primary component of epoxy leachates. However, when chlorine is present in feed water, chlorinated BPAs and TCP are also eluted from epoxy materials [366,367]. Thus, the contribution of chlorinated BPAs and TCP may not be neglected since TCP is known as a harmful foulant in RO systems.

One of the origins of BPA is from the permeate spacer. Currently, two types of permeate spacer exist [368]. One type of spacer is made of a bicomponent polyester material or a mono-component polyester, which is then coated with epoxy to provide stiffness [369]. The other type is non-epoxy-coated. This type of spacer was developed to address an issue of higher permeate TOC when applying RO to UPW application. A new type of spacer without melamine or epoxy resins was invented in which the fiber threads are fixed to each other with a hot melt fiber adhesive [370]. 

A spacer supplier mentioned that the epoxy-coated spacer has a limiting factor arising from the fact that during use, an unidentified chemical leaches out of the epoxy-coated fabric into the permeate stream [371]. In a two-pass membrane system, the second-pass membrane becomes partially fouled. In extreme cases, the second-pass RO membrane is fouled almost immediately to the extent that it loses up to 50% of its flux [148]. Non-fouling epoxy resin system for the permeate spacer was disclosed to mitigate this issue [371].

#### 6.7.3. Leachables from Spiral RO/NF Elements

Sometimes, leachables from the RO/NF elements themselves can cause a significant decrease in flow rate. The effect of leachables from the permeate spacer has been outlined. Sources of organic leachable components include organic solvents remaining during the formation of the porous support, leachables from fabrication materials composed of non-woven fibers used for the support, feed and permeate spacers, an anti-telescoping device (ATD), and FRP [372]. In addition, residual monomers, e.g., m-phenylenediamine (MPD), TMC, and its hydrolyzed acid and additives, are contained in a new element. Some RO membranes are coated with glycerin, a humectant that prevents drying [373]. The concentration of the humectant in flat sheet RO/NF membranes was evaluated by measuring TOC [374]. However, the TOC values may contain contributions from other monomers. When analyzing the NF (NF90) membrane leachables, liquid chromatography detected two large peaks. One of the detected peaks was identified as MPD [375]. 

It was observed that leachables from new membranes contribute to precursors of N-nitrosodimethylamine (NDMA) DBP formation [376]. However, since the actual TOC level quickly decreases during operation, the NDMA formation may not be a critical problem in municipal water treatment. It is reported that permeate TOC testing with a single element is decreased to less than 20 ppb after 24 h of operation [377]. To further reduce permeate TOC concentration and achieve quick rinse-down performance, oxidative treatment, such as chlorine, hydrogen peroxide, etc., was proposed [372,378]. It was speculated that MPD reacts with chlorine to convert the amine groups in MPD to chloramines, and then a part of the MPD is decomposed. This method is consistent with the proposal that washing membranes with a small dose of chlorine (e.g., 1 mg/L) is a method for minimizing the leaching of NDMA precursors [376].

There are two main cases where the leachables from RO/NF elements cause an operational problem: a double-pass RO system where the permeate from the first element is fed to the next membrane and a membrane system where the concentrate or permeate is recycled to the feed water. Brine recirculation is applied to increase system recovery in small- to medium-sized plants. Recently, a new RO process called batch RO has been attracting attention, in which the brine exiting the RO module is recirculated back to the feed side without mixing with fresh feed [379]. Permeate recycling is sometimes applied, e.g., in pharmaceutical and dialysis water production systems where the excess permeate water is recirculated back to the inlet of the RO [380,381]. For hot water sanitization RO systems, hot water is recycled within brine and permeate lines. When hot water sanitization is applied soon after installing new membranes, great care must be taken as a high concentration of leachables may be generated.

**Figure 11:**
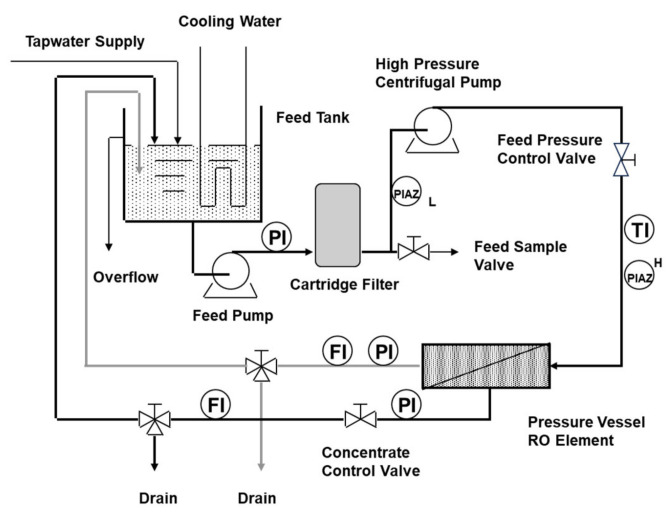
Schematic flow diagram of experimental element testing unit.

As for RO/NF membrane testing units, both permeate and concentrate water are typically recycled, as shown in Figure 11. A cartridge filter is usually placed in front of the RO.

Wagner et al. [382] performed crossflow filtration experiments to characterize the three commercial PA RO membranes. A more significant increase in salt rejection and decline in permeate flux was observed when the recycled water was directly sent to RO. On the other hand, flux and rejection showed somewhat stabilized performance when an AC filter was installed. They speculated that particulate matter introduced from dust or wetted stainless steel parts might cause significant flow loss. 

Since the AC filter is proven to prevent the leachable fouling originating from RO/NF elements [144], the AC filter should be installed to obtain the correct inorganics performance, i.e., non-fouling performance. For this purpose, an AC fiber cartridge filter might be suitable because there is no carbon-fine leakage [383]. However, tests should be done without the AC filter after thorough flushing with pure water when evaluating organic compounds.

#### 6.7.4. Leachables from Ion Exchange Resins

In UPW production, ion exchange resin processes are sometimes installed in front of RO, where the primary role of RO is to remove residual TOC. Those processes include cation and anion resins (two-bed, three-tower pure water system, 2B3T), a mixed bed ion exchange unit, etc. Although almost all inorganics are removed in the 2B3T, flow loss has occasionally been reported [100,139]. In the 2B3T process, leachables from anion exchange resins are suspected as a cause of RO fouling [384]. The mixed bed ion exchange resin generates high TOC leachables after regenerating the resins [100]. A long-term fouling phenomenon was reported for the mixed bed ion exchange unit [385]. 

Ikeda reported the effect of leachables from various ion exchange resins (macroporous and gel-type resins) on TFC RO membrane performance [195]. None of the resins induced appreciable flow decline. Thus, leachables after long-term operation may significantly impact membrane performance since the anionic resins degraded ∼1.6 to 7 times faster than the cationic resins [386]. Agui et al. [387] analyzed the leachables from anion exchange resins (OH form). The major TOC component was trimethylamine (about 50% or greater of the TOC values). The high MW leachables ranged from above five hundred to less than ten thousand. According to reverse phase chromatography, three unknown prime substances were identified in the low MW leachable components. The chemical structures of the two substances were speculated as phenol and benzoic acid quaternary ammonium derivatives. Trimethylamine is also known to cause some degree of flux reduction [195]. Thus, trimethylamine and quaternary ammonium derivatives could be the substances that cause flux loss.

In order to prevent deterioration of the RO membrane caused by leachables from the ion exchange resin, the following measures have been proposed, e.g., installing a cation exchange resin unit after anion resin [384] and using low surface zeta-potential RO membranes [385].

### 6.8. Industrial Wastewater

**Figure 12:**
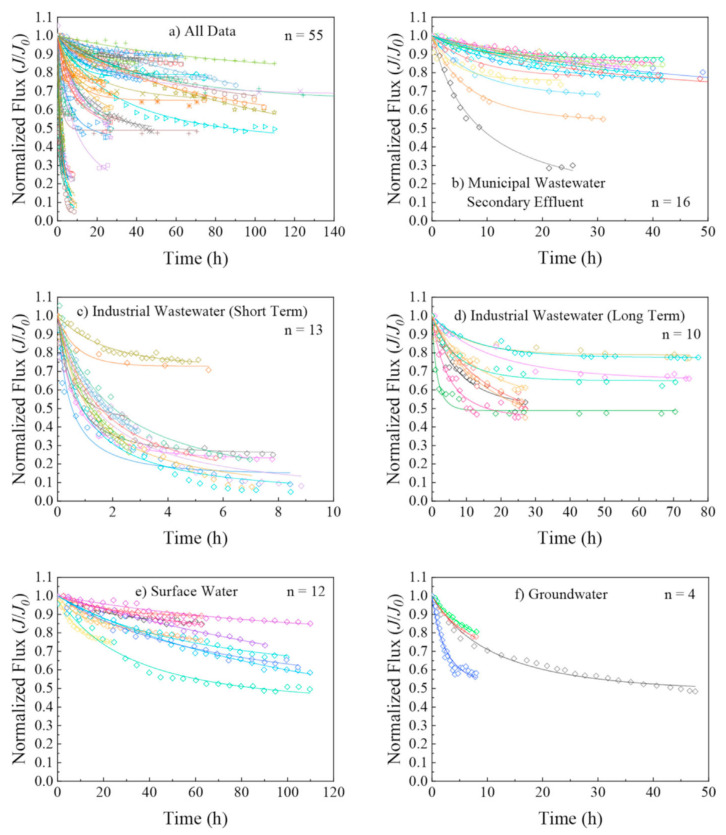
Changes of initial normalized flux for various feed sources [160]. Reprinted with copyright permission.

Xu et al. [160] summarized the normalized flux changes of RO membranes treating various water samples, including municipal secondary effluent, treated industrial wastewater, surface water, and groundwater, as shown in Figure 12. 

In the data sets, the initial membrane flux was 2.76–377 LMH, and the majority (10~90%) was 13.2–190 LMH. Since it is reported that the flux loss behavior depends on foulant types, initial flux, membrane type, etc. [388], it may not be easy to speculate the exact cause of fouling by simply observing the initial flux. However, the flux decline trend for most industrial wastewater differs from municipal wastewater and surface water, i.e., quick flux decline and fast stabilization within 10 h. Thus, specific LMWOCs may partly contribute to the fouling of industrial wastewater treatment. Such cases are listed below as examples:

- Cooling tower blowdown (CTBD) treatment: [389,390,391];
- Pulp and paper industry: [138,392,393,394,395,396];
- Petrochemical industry: [312,397];
- Oil and gas industry: [337,398,399,400,401];
- Carwash industry: [260,402];
- Pharmaceutical industry: [313,403,404];
- Textile industry: [254,405,406,407,408,409,410];
- Tannery industry: [411].

Recovery of CTBD water is one of the important wastewater reuse targets in industries. However, fouling is a critical concern for RO treatment since the CTBD water contains many potential foulants to RO, e.g., high BOD for biofouling, particles, and chemicals [391]. Conditioning chemicals added to the cooling tower water include biocides, corrosion inhibitors, and scale inhibitors (antiscalants) [412].

Löwenberg et al. [389] investigated three different pretreatment technologies: powdered activated carbon (PAC) adsorption, coagulation with ferric chloride, and UF. The LC-OCD analysis evaluated the capability of the pretreatment options to remove DOC. The LC-OCD showed complete biopolymer removal, partial humic substance removal, and building block removal at concentrations above 20 mg/L of Fe^3+^ and pH 5.5. With PAC adsorption, a slight removal of all DOC fractions with a preference towards LMW substance adsorption was achieved. UF treatment substantially reduced turbidity, while the DOC concentration remained unchanged.

Regarding fouling tests, the pretreatment by Fe^3+^ with subsequent UF showed no improvement in RO performance. Meanwhile, PAC/UF pretreatment improved the RO performance (by an average of 32%) compared with untreated CTBD. This evidence shows that LMWOCs have more influence on RO fouling. Investigation of the chemical additives revealed a biodispersant, which is believed to considerably impact membrane fouling.

## 7. LMWOC Contributions in NOM Fouling

Fouling by NOM can occasionally be an operational problem; therefore, many research reports have been written on this subject. However, as mentioned in the previous section, it is not an acute problem in practice, but in most cases, the flow rate gradually decreases. In such cases, it is possible to continue the stable operation of the plant by performing membrane cleaning in a timely manner. 

NOM is found in groundwater, surface water, and seawater and exists as a component of EfOM in sewage. Many RO/NF plants have been operating in Florida, USA and elsewhere to remove NOM (DBP precursors or color) from groundwater. Thus, many pilot tests and the operating results of actual plants have been reported. According to their results, in most cases with RO/NF treatment of groundwater, the initial flow decline rate is minor and often continues to decrease gradually over a long period [163,164,165,413,414,415,416,417,418,419,420,421,422]. Jin et al. [423] analyzed HA as fouling by organic colloids based on light scattering measurements. It may be possible to conclude that the long-term flow rate decrease is due to gel or cake layer formation by the HA colloids. 

Nevertheless, an appreciable initial flow loss was occasionally observed when acid and an antiscalant were added to high TOC water [415]. It was found that lowering the pH destabilizes the NOM, causing them to coagulate more quickly in the presence of antiscalants and contributing to fouling. After eliminating the pH adjustment, the adverse effect on performance disappeared. The same phenomenon was also observed in a pilot test for surface water treatment from the Rhine River, although the TOC level was not so high, about 1.5 mg/L [424,425,426]. Significant fouling was observed when operating the pilot plant at a high recovery of 90%, along with sulfuric acid and antiscalant injection. By eliminating the antiscalant, the plant performance was stabilized. In this case, the poor performance was attributed to biofouling induced by the antiscalant, which acts as a nutrient for biological growth. However, since sharp flow loss occurred in all three stages and no induction time was observed after CIP, organic fouling may also have to be considered.

Surface water may be affected by urban or agricultural activities, and depending on the intake location, feed water may contain SOCs in addition to NOM. This composition variation makes the behavior of fouling complex [424]. In lake or river water, where there is little human activity, fouling behaves similarly to groundwater [427,428,429,430,431]. However, since surface water generally contains a lot of silt and colloidal components, it may show signs of colloidal fouling, i.e., continuous flux decline, if pretreatment is insufficient [432,433,434,435]. In such cases, applying UF for pretreatment makes it possible to stabilize the operation of the RO/NF membranes [78,167,436].

Notably, an initial decrease in flux is observed in surface water affected by urban or agricultural activities [437,438,439]. During a pilot test at the Heemskerk water treatment plant in the Netherlands, a decrease in the initial MTCw of about 20% was observed. By adding GAC filtration in front of UF at the full-scale plant, the MTC of RO remained stable from the beginning, and for over three years after start-up, no chemical cleanings were applied [440,441].

To eliminate the effects of particles/colloids, evaluating the isolated NOM from surface water might be a way to elucidate the effect of NOM itself. A water sample was taken from a reservoir consisting of upland forest and mixed agricultural/residential development [442]. NOM was isolated and purified using a field RO system, a cartridge filter, and a softener. When an NF membrane was tested with 10 mg/L of NOM in 0.01 and 0.05 M NaCl background solutions, no immediate flow loss was observed, but about 10% was observed after 10 h. Lee and Elimelech [443] obtained a model NOM powder from river water by applying freeze-drying as a final step and conducted NF fouling tests. In addition, a silica colloid was used to investigate the mechanisms of combined fouling of NF membranes by the co-occurrence of both colloids and NOM under different Ca and NaCl concentrations. NOM (20 mg/L) fouling with 1 mM NaCl solution was minor, but flux decline was enhanced when NaCl concentration was increased to 10 mM. NOM fouling in the presence of Ca^2+^ is much more severe. The role of Ca ions in NOM fouling is due to the specific binding of the Ca ions to acidic functional groups of NOM. Furthermore, a mixture of NOM, colloids, and calcium ions showed a sharper initial flux loss.

NOM in water consists of organics with a wide range of molecular weights, e.g., biopolymers, HA (and fulvic acid), building blocks, LMW organic acids, and LMW neutrals. For the long-term effect, the colloidal nature of NOM may play a more important role in RO/NF fouling. However, knowing which fraction of organic matter has a more significant impact on short-term fouling would be of value. Although there is little research in this field, there are reports that medium to LMW organics exhibit more significant fouling behavior [413,444,445]. 

Nilson and Digiano [446] fractionated NOM collected from a river into hydrophilic and hydrophobic fractions by the XAD-8 resin and pretreated with PAC. Hydrophobic NOM was shown to be responsible for the decline in the permeate flux of the PSF hollow fiber NF membrane. LMW (<1 kDa) and high MW (>30 kDa) portions of NOM were increased after PAC treatment. This portion of NOM showed a more significant initial flux decline. It was explained that PAC pretreatment preferentially removes some of the hydrophilic NOM.

Higashi et al. [447] investigated the effect of different MW fractions of NOM on the performance of an RO membrane. The NOM from Lake Biwa (Japan) was fractionated into three components by two UF membranes (MWCO: 30 kDa and 1 kDa). The Suwannee River NOM was fractionated into two components by UF (MWCO: 1 kDa). In this fractionation, hardness was removed by a softening resin. The lowest MW fraction (≤1 kDa) exhibited the worst flux decline for both samples. Cleaning tests were conducted for the Suwannee River NOM to confirm whether the flux could be restored and whether the fouling was reversible. The performance of the unfractionated membrane was almost 100% restored by alkaline cleaning. On the other hand, the fouled membrane with the fractionated NOM (≦1 kDa) did not recover the flux after cleaning (alkaline and acid). Regarding the fouling mechanism, it was speculated that LMWO components could be adsorbed and diffused into the membrane, resulting in pore blocking.

## 8. EfOM Fouling in RO/NF Processes

The OCWD started a 5 million gallon per day RO treatment facility in 1977, the largest in the world operating on municipal wastewater at that time [448]. This unit was a part of the district’s advanced wastewater treatment plant known as WF-21. The FT-30 elements were tested at OCWD in 1981 [38]. However, continual loss of flux was encountered. Simple cleaning trials were ineffective. Later, two additional TFC PA membranes were pilot-tested at the WF-21 [136]. The permeate flow of both membranes prominently decreased. Cleaning gave little improvement in the flux for one type of membrane. Due to these results, CA membranes have long been considered suitable for wastewater treatment. However, it was recognized that the TFC RO membranes have better TOC and micropollutant removal capability than CA membranes.

This situation began to change in the mid-1990s, and the following factors can be considered as promotors for switching from CA to TFC PA membranes:

- Many pilot tests carried out during this period gained a greater understanding of TFC PA membranes;
- Reliability improved as actual plants were put into operation without any significant issues;
- Higher-quality water has been required for reuse applications (indirect potable reuse, boiler make-up water, etc.);
- MF/UF has been utilized as pretreatment for secondary effluent.

In the mid-1990s, many pilot tests were conducted in the US. Freeman and Morin [449] summarized some of them and reported that most facilities use CA membranes, and the application of PA membranes has not been as successful. The failures with PA membranes were mostly due to severe, unrecoverable flux decline. However, it was recognized that some of the TFC RO membranes ran well at the Vero Beach facility and a test unit at WF-21 with a lime softening pretreatment scheme. In the Vero Beach plant, the success of the PA membranes was seen when bituminous AC was used for pretreatment. The bituminous carbon may remove fouling organic species that other pretreatment methods have not removed. In the WF-21 study, TFC HR RO membranes (Fluid Systems) operated on MF-treated effluent containing 3 to 5 mg/L combined chlorine at pressures less than 200 psi. Since similar results were obtained from other tests, Leslie et al. [450] mentioned that acceptance of the TFC RO membrane should be increased as an alternative to CA in full-scale municipal wastewater reclamation plants. 

The relative flux (J/J_0_) has been utilized to compare TFC RO with CA membranes. A significant relative flux decline was observed for TFC RO, but the absolute flux is still higher than that of CA at stabilized conditions. This advantage was recognized in a pilot study in the City of San Diego. At 10 gfd (gallons/ft^2^/day) flux, the CA membranes exhibited an average pressure over the 2500 h test of 215 psi. Meanwhile, the TFC RO units exhibited an average pressure of 130 psi. The specific flux (gfd/psi) was 0.045 and 0.08 for CA and TFC membranes, respectively. This translates to a significant operating performance advantage for TFC over the CA membranes [451].

Large-scale wastewater treatment plants utilizing TFC RO were first built in India. The Madras Refineries Ltd. built a plant (9550 m^3^/d) in 1991 with A-15 (DuPont) [452] and a second plant (8600 m^3^/d) in 1993 with the APA membrane (BW30, FilmTec). The BW30 membrane encountered an initial rapid flux decline but maintained the relative flux (or fouling factor) at around 0.75 [453]. After gaining more insight into the fundamental characteristics of TFC RO in wastewater and operational data in actual plants, the TFC RO has come into widespread use. 

Nevertheless, the initial rapid flux decline is still a significant concern for wastewater applications. Bartel and Franks [176] speculated that the neutral, hydrophilic fractions with low MW are the key foulant for an initial flow loss and tend to “plug” the larger macro-cycles (aggregate pores) within a PA matrix. Since the fouling symptoms are similar to those observed for LMWOCs and internal fouling, several comparisons are made in the following subsections.

### 8.1. LMWOCs in EfOM

EfOM contains many organic substances with different molecular weights, NOM, SOC, and SMP [110], and has an average MW of around 1 kDa in MF/UF prefiltered secondary effluent [454]. It was reported that long-chain fatty acids and alkylphenol polyethoxy residues were the major contributors to the permeate TOC of the CA membrane in the WF-21 [136]. This result may indicate the presence of relatively high concentrations of these LMWOCs in the pretreated feed water. According to contaminant analyses by the U.S. Geological Survey from streams being susceptible to contamination (i.e., downstream of intense urbanization and livestock production), three groups (detergent metabolites, plasticizers, and steroids) contributed almost 80% of the total measured concentration [455]. Luo et al. [456] summarized the occurrence of micropollutants and removal during wastewater treatment. Micropollutants occurred in wastewater influent in the concentration range between 0.1 and 10 μg/L, while some pharmaceutical compounds, a biocide (triclosan), nonylphenol, and DEHP exhibit relatively high occurrence concentrations. Wang et al. [457] reported micropollutant concentrations in secondary effluent in China. These include polycyclic aromatic hydrocarbons, phosphorus flame retardants, phthalates, benzothiazoles, and phenol. These compounds may have adverse effects on the TFC RO membranes.

### 8.2. Initial Rapid Flux Decline and Salt Rejection Improvement

Numerous data are available, from lab and pilot tests to full-scale plants [176,458,459,460,461,462,463,464,465,466]. As seen in Figure 12, the behavior of the flow rate decline in municipal wastewater treatment can be roughly divided into two types: first, where the flow rate declines rapidly at the beginning, then stabilizes or does not decline much further, and the other where the flow rate continues gradually declining after the initial decline. These different types of fouling behavior may depend on the composition of the EfOM, pretreatment, RO membrane type, experimental conditions, etc. The first type of behavior can be seen in pilot tests conducted at OCWD and the WBMWD, where MF was used as pretreatment [174,175,467,468,469,470,471,472,473,474,475]. OCWD and WBMWD conducted independent studies to determine if low-fouling or fouling-resistant membranes would perform better in municipal wastewater treatment in 2001–2002 [175]. However, it was found that the low-fouling membranes did not operate at lower operating pressure than the previously tested membranes. The stabilized specific flux of the low-fouling elements was 0.06–0.07 gfd/psi, while the ULP RO (ESPA2) showed 0.08–0.09 gfd/psi flux after 5000 h of testing. Subsequently, OCWD focused on evaluating ULP RO elements, which typically showed 0.11–0.14 gfd/psi stabilized flux (acceptable minimum specific flux of 0.085 gfd/psi). 

Pretreatment also affects the secondary flow decline. The use of UF as pretreatment minimized the degree of fouling compared with non-UF-treated tertiary effluent [462]. Using UF for TFC RO reduced the average operating pressure by 33% compared with MF at a pilot test in OCWD [450]. Reardon et al. [476] conducted a pilot study to gain insight into which MF and UF are more suitable as pretreatment for RO. Initial fouling tends to be stabilized for UF-treated secondary effluent. However, for MF, the flux continued to decline. Based on these observations, the secondary flow decline might be partially caused by HA and colloidal components that form gel or cake layers. Since high molecular weight components, e.g., HA, vary with treatment processes and seasons, these may affect the fouling behavior [463].

Care must be taken in lab and bench tests since the apparent flow rate may decrease due to a possible increase in feed water concentration or the effects of substances eluted from the membrane and equipment during recirculation.

### 8.3. Effets of AC to Eliminate EfOM Fouling

As mentioned, AC treatment can reduce fouling caused by LMWOCs. A similar phenomenon has been reported in municipal wastewater treatment. Geselbracht and Freeman [477] reported that AC pretreatment was successful for TFC RO membranes. Originally, lignite-based carbon was used in the GAC filters. However, particulate matter was increased after repeated backwashing due to carbon friability. It was found that bituminous-based carbon has a greater density and smaller pores for more significant adsorption per volume, with a stronger attraction for the smaller, volatile organics [478]. Using a secondary effluent with 18.9 mg/L TOC, column testing provided an effluent TOC of 4.1 mg/L with the lignite-based carbon and an effluent TOC of 2.1 mg/L with the bituminous-based carbon. Similar results were obtained when GAC was applied to MBR effluents [479]. The DOM removed by GAC pretreatment is composed mainly of hydrophobic and biodegradable components.

On the other hand, it was reported that RO fouling is more pronounced in AC-treated water (media filtration followed by AC treatment) than in UF [480]. These differences may be explained by the results of a comparative pretreatment test conducted by Shon et al. [481,482,483,484]. In this study, four different pretreatment methods, ferric chloride flocculation, PAC adsorption, flocculation followed by adsorption, and GAC biofilter, were compared to examine UF membrane (NTR 7410) fouling. Their findings revealed that the UF membrane fouling was significantly reduced by treating the biologically treated sewage with flocculation followed by PAC compared to treatment with GAC or PAC directly. The same results were obtained for NF membranes (NTR 729HF).

Secondary effluent contains organic matter of various MWs, which may compete for AC adsorption, reducing the capability of LMWOC removal. In addition, the removal performance of LMWCOs may differ depending on the type of AC. For example, tests at WF21 showed that the removal of plasticizers, DnBP and DEHP, is lower than that of DMP and DEP [485]. Thus, it is crucial to remove high MW components in advance and select appropriate AC types to improve effectiveness.

### 8.4. Effect of Membranes on Initial Fouling

Many RO/NF membranes have been evaluated for suitability from the aspect of fouling, micropollutant removal, costs, etc. [449,477,486]. These include CA membranes, APA and PPA membranes, PVA membranes, sulfonated PES, etc. [462,468,487,488]. Among those, the CA membranes are well known for their low fouling propensity and have almost no initial flow rate drop. As mentioned, APA membranes typically show initial rapid flow decline. Gagliardo et al. [451] reported that the initial specific flux of the TFC RO membranes (0.16 gfd/psi) decreased to 0.10 gfd/psi after a few days of operation. Sulfonated PES membranes also have the same characteristics [482,483,488].

Meanwhile, certain types of RO/NF membranes were reported to show a less severe flow drop [486,489,490,491]. It has been reported that PVA membranes and PPA NF membranes have minor initial fouling compared to APA membranes [488]. Simple acid cleaning (0.2% oxalic acid) was enough to restore the initial flux for those membranes. However, the acid cleaning alone was insufficient for the APA membrane; the flux decreased gradually after several cleanings. These fouling propensities are consistent with those observed with surfactants.

Currently, APA membranes have been used in many wastewater treatment plants. As shown in Table 17, APA membranes are classified into four types based on their operating characteristics and rejection performance [492]. Among those four types, SULP RO and APA NF membranes, in particular, cause a significant flow drop compared to LP RO [458,462,470,491,493]. Bellona et al. [468] examined the influence of membrane type in lab and pilot tests. One of the NF membranes, NF-90, exhibited twice the flux decline (30%) of ULP RO membranes, TMG10 and ESPA2 (15–16%) but maintained a greater specific flux because of its high initial permeability. Even though the initial flux varies for ULP RO and APA NF membranes, the final specific flux does not differ much, i.e., 0.025–0.03 LMH/kPa (0.10–0.12 gfd/psi) [470].

On the other hand, the degree of initial fouling of PPA NF membranes is minor [491,494,495]. According to a study by Bellona et al. [494], methanol, ethanol, and urea rejections are increased significantly after fouling tests in the APA NF membrane (NF-90). In contrast, no significant change was observed in the PPA NF membrane (NF-270). The same behavior was observed for various RO/NF membranes [491,496]. Salt rejection (conductivity-based) of SULP (XLE) and NF90 membranes was increased but not for LP RO and PPA NF membranes [491]. Fujioka et al. [496] reported that the rejection of LMW N-nitrosamines such as NDMA was increased by membrane fouling, and the impact was pronounced for membranes that have high membrane permeability. LMWOCs are thought to be trapped in aggregate pores within ULP and SULP RO membranes due to the strong interaction between membranes and foulants [176,494].

In addition, the PPA membrane has a smooth surface compared to the rough structure (ridge-and-valley) observed in ULP RO membranes, which may also affect the low fouling properties.

### 8.5. Contributions of LMWOCs to RO/NF Fouling by EfOM

When treating secondary or tertiary effluents with RO, the initial flow decline cannot be suppressed even when LP membranes are used in pretreatment. This indicates that LMWOCs are undoubtedly involved in RO/NF fouling. Generally, UF cannot reduce TOC, including humic substances. Antony et al. [497] reported that the molecular weight of the humic substances contained in municipal wastewater varies from 355 to 503 Da. Studies have been conducted to determine which composition (hydrophobic or hydrophilic) and molecular weight components are involved in initial fouling.

No significant differences were observed when EfOM was fractionated using ion exchange resins. Jarusutthirak et al. [459] investigated fouling behavior by fractionating EfOM into colloids, hydrophobic (HPO), and transphilic (TPI) components. While colloidal components showed a somewhat significant flow rate drop, no notable difference was observed for HPO and TPI. Liquid chromatography analysis shows that HPO and TPI contain low-molecular-weight components of the same order, including humic substances. Similar results were seen in the study by Zhao et al. [454], who fractionated EfOM into four fractions, i.e., aquatic humic substances, hydrophilic acids, hydrophilic bases, and hydrophilic neutrals. Although their study showed interesting results, such as differences in fouling behavior due to the effect of calcium ion, no significant differences were observed in the initial flow rate drop among the fractions.

Zhang et al. [498] investigated fractioned samples using UF membranes with MWCO ranging from 2 to 100 kDa. As a result, they found that hydrophobic components with MW of 2 kDa or less, in particular, exhibited high fouling propensities. Fujioka et al. [499] conducted fractionation experiments focusing on low MW. Three UF (MWCO: 1 kDa, 10 kD, and 50 kDa) and one NF membrane (MWCO: 300 Da) were used for sample fractionation. The same initial fouling behavior (rapid normalized pressure increase) was observed between the 1 and 50 kDa samples for the ULP RO (ESPA2). This result indicates that the MWs of the major foulants are less than 1 kDa. When feeding the NF permeate to the RO, the extent of fouling was reduced. A similar fouling tendency was observed for the LP RO membrane (BW30). Although some previous publications [176,465] have pointed out that low MW components contribute to the initial flow rate decrease, this report revealed the contribution of significantly lower MW substances. Characterization of fouling substances in the NF-treated wastewater found that fouling substances contained LMWOCs such as tryptophan (MW = 204.23 g/mol) (or tryptophan-like substances).

Fujioka reported that rejection of N-nitrosamines such as NDMA was increased after RO membranes were fouled with tertiary effluent [496]. In addition to EfOM, the effect of several model fouling substances (sodium alginate, BSA, HA, or colloidal silica) on the rejection behavior of N-nitrosamines was evaluated and found to be considerably less than that caused by tertiary effluent. Thus, it was speculated that enhanced NDMA rejection can occur by forming a densely packed fouling layer with LMWOCs on the membrane surface, in which the clearance is too small for NDMA to pass through. These findings should support the understanding that the initial flow loss is due to LMWOCs in EfOM. 

## 9. LMWOC Fouling for Other Membrane Processes

Fouling by LMWOCs is not limited to RO/NF. A certain LMWOC strongly interacts with polymeric membranes and induces fouling [67]. Antifoaming agents such as polypropylene glycols (PPGs) are reported to cause rapid fouling of certain UF membranes [500,501]. Although this review does not intend to elaborate on the fouling of LMWOCs for any other membrane processes, some examples are shown in this section since the LMWOC fouling can be ubiquitous in membrane processes, and its mechanism may have some similarities with RO/NF. In Table 18, membrane processes and their LMWOC foulants are listed.

In the past, fouling was believed to be virtually absent in pervaporation and gas separation with dense membranes [53]. However, it has been known that certain types of membranes are fouled with LMWOCs, e.g., poly[l-(trimethylsilyl)-1-propyne] (PMSP). However, since PMSP is a high free volume polymer, it is uncertain whether it is appropriate to call it a dense membrane. PMSP is known to be the polymer with the highest gas permeability [502]. However, its transport characteristics are unstable due to an aging effect [503]. Nagai et al. [504] summarize the impact of contamination or fouling on membrane transport phenomena. Sorption of vacuum oils and plasticizers, such as dioctyl phthalate (DOP), into the PMSP membrane were indicated as typical foulants during membrane storage and evaluations. Okamoto et al. [505] reported the free volume distribution of PMSP and changes after DOP vapor sorption, estimated by positron annihilation lifetime spectroscopy (PALS) measurements. The PMSP has a bi-modal distribution of free volume holes. The estimated average size of large free space holes is 3.0 nm^3^. When DOP vapor was sorbed in PMSP films, the intensity of the large pores (1–7 nm^3^ ranges) decreased, and that of the small pores (below 0.7 nm^3^) increased, probably because of filling up free space holes with larger size. This result suggests that the decrease in gas permeability is due to LMWOC sorption or internal fouling.

The following example may not be an exact fouling case but a case in which the residual solvents act as an inhibitor of gas transport. Ahmad et al. [506] measured the gas permeability of PES membranes cast from different solvents, i.e., N,N-dimethylacetamide (DMAc), N,N-dimethylformamide (DMF), and N-methyl-2-pyrrolidinone (NMP). From thermo gravimetric analysis (TGA), residual solvents were detected, and the amount of residual solvent was in the order of NMP > DMF > DMAc. PES-DMF and PES-DMAc membranes displayed an order of magnitude higher gas permeability than PES-NMP membranes.

The author’s group experienced the same phenomenon when measuring PES gas permeation [507]. First, the PES membrane was cast with dimethyl sulfoxide (DMSO) as a solvent. However, gas permeability was quite low compared with membrane film prepared by compression molding. Further analysis found that the residual DMSO causes low permeability and acts as an antiplasticizer that reduces the free volume of the PES membrane [508,509]. 

**Table 18:** Fouling of LMWOCs for various membrane processes.

Membrane Process	Material	Foulants (LMWOCs)	References
Gas transport Gas separation	PMSP	DOP (rubber gaskets in permeability testing)	[505,510,511]
	PMSP	Vacuum pump oil vapor	[504,512,513,514,515]
	PES	Casting solvent such as NMP	[506]
Low Pressure Membrane (LPM) MF UF	NA	Antifoaming agents such as PPGs	[41,61,516]
	Noncellulosic	Nonionic detergents	[517]
	PSF	Nonionic surfactants (Triton, Dobanol)	[518]
	PSF	LMW anion-exchange resin leachables	[519]
	PSF	Nonionic surfactant as a model antifoam	[520]
	Acrylic polymer	Tannic acid	[521]
	PES	Tannic acid	[522]
	PSF, PES, Polyaramide	Octanoic acid	[523,524,525,526,527,528]
	PES	Nonionic surfactant (Triton X100)	[529]
	PES, RC, PVDF	Medium to LMW component of NOM	[530]
	PES	Abietic acid	[531]
	PES	Cationic surfactant (CTAB)	[532]
Pervaporation (PV)	PMSP	Organics in ABE fermentation broth Lipids, stearate, palmitate, diols, etc.	[533,534,535,536]
	PMSP	Butyric acid and long-chain fatty acids	[537]
	Zeolites, Microporous silica	Amides (DMF, DMAc), glycols and phenol	[538]
	LTA zeolite	Long-chain hydrocarbon	[539]
	ZSM-5 zeolite particles dispersed in silicone rubber	Organic acids, esters, alcohols, and esterification reaction products	[540]
	ZSM-5 zeolite	Contaminants in bio-oil	[541]
	T-type zeolite	Contaminants in bio-oil	[542]
Membrane Distillation (MD)	Polypropylene	Methylene blue	[543]
	Polypropylene	Dye (Methylene blue, indigo, acid red 4, etc.)	[544]
	Polytetrafluoroethylene	HA (LMW organics)	[545,546]
Electrodialysis (ED)	Cation exchange membrane	Strong organic bases (MW > ca. 350)	[547]
	Anion exchange membrane	Strong organic acids (MW > ca. 300)	[547,548]
	Anion exchange membrane	Dodecylbenzene sulfonate	[549,550,551,552]
	Anion exchange membrane	Fatty acid and an anionic surfactant	[553]
	Anion exchange membrane	Aromatic amino acid (phenylalanine)	[554]
Forward Osmosis (FO)	Not disclosed	Octanoic acid	[555]
	Cellulose triacetate (CTA)	Octanoic acid	[556]
	TFC PA	LMW organics in municipal wastewater	[557]

RC: Regenerated cellulose, PVDF: Polyvinylidene fluoride.

## 10. Mechanism of Fouling with LMWOCs

When considering the mechanism of fouling by LMWOC, the first critical aspect to consider is the relative importance of surface fouling versus internal fouling [68,79]. To gain insight into this issue, it is helpful to compare the symptoms of fouling by LMWOC with those of other foulants primarily associated with surface fouling. A comparison with other types of membrane fouling is shown in Table 19.

Fouling caused by LMWOC is characterized by a rapid drop in flow rate (1–2 h in laboratory tests) shortly after contact, which is not generally seen with other foulants such as NOM, as mentioned in the previous section.

For example, in the case of silica colloid fouling, the extent of initial flow drop depends on the concentration, ionic strength, permeate flow rate, flow velocity, membrane surface morphology, etc., [157,443,558,559,560]. Associated with the decrease in permeation flux, silica colloids were observed to accumulate on the membrane surface [560]. It has also been reported that the membrane surface flow velocity has a significant effect, and fouling is reduced at high linear flow velocities [561]. Regarding the impact of foulant concentration, in an experiment using a high concentration of 200 mg/L silica colloid, it took about 5 h for the flow rate to drop by 30%, even for the membrane with the most severe fouling [559]. In addition, results of lab-scale experiments showed that colloidal fouling caused a marked decrease in the rejection of inert organic molecules with molecular weight smaller than about 100 g/mol [562].

The next example is a case of adsorption of polycations. No significant flow decline was observed in fouling caused by the adsorption of PEI or polycations with quaternary amine groups in the main chain [195,221,223]. However, when PEI was adsorbed at a high concentration of 2000 ppm, the pure water flux dropped by 40%. When this membrane was fouled with DTAB, a further rapid drop in flow rate of 20–30% was observed [246], suggesting that fouling mechanisms due to PEI adsorption and DTAB fouling are different. Furthermore, the degree of flow rate drop was insignificant even when the membrane surface was modified through layer-by-layer adsorption of polycations and polyanions (as for 2–3 layers) [227].

As for cleanability, as mentioned in the NOM section, in a fractionation test using NOM from the Suwannee River, the permeate flow rate of the membrane fouled with NOM was restored by alkaline cleaning, whereas the components fractionated by UF (<1 kDa) showed poor cleanability [447].

As it is difficult to identify foulants using regular surface analysis such as FT-IR, it can be assumed that fouling by LMWOCs is mainly caused by internal membrane fouling.

### 10.1. Evidence of Internal Fouling

The importance of internal fouling caused by LMWOC has already been mentioned, but some supportive experimental results are shared here. It is known that TCP causes a significant decrease in flow rate among phenolics. Williams et al. [178] measured the chlorine atom concentration inside the membrane using ESCA combined with the Ar+ ion etching technique. The TCP-treated membrane has chlorine atoms present in the thickness direction of the membrane at about 20–30% concentration. Similar data were reported for PFOS [204]. PFOS is known to induce a decline in the flow of RO/NF membranes. After a total etching time of 240 s, corresponding to a nominal etched depth of 42 nm, the F signal was still measurable for the BW30 RO membrane. 

When a PA NF membrane (MWCO = 400) with a double skin layer, which is different from conventional TFC membranes, was treated with a dye, it was confirmed by optical microscope measurements that the dye was trapped in the entire membrane [306].

Next is the case that internal fouling could be speculated by measuring changes in the LMWOC feed concentration and rejection during a continuous filtration experiment. Kimura et al. [563] reported that, from sorption and filtration experiments, the sorption of hydrophobic compounds was more significant in dynamic tests than in static tests. This result suggests that more sorption sites are accessible for molecules during membrane filtration, i.e., internal pores or free volume. It was also found that the initial permeate concentration was lower due to the effect of sorption. 

NF filtration experiments (TFC-SR2) were conducted using three pharmaceuticals with similar molecular weights (sulfamethoxazole, carbamazepine, and ibuprofen) as model substances for pharmaceuticals [564]. For sulfamethoxazole and carbamazepine, which have low rejections, no change was observed in the feed and permeate concentrations during the circulation test at pH4. In contrast, the feed concentration decreased during the test for hydrophobic ibuprofen, indicating the effect of sorption into the membrane. Due to this sorption effect, the concentration of ibuprofen in the permeate gradually increased, reaching a steady value after 2.5 h. Despite the similar molecular weights of these three pharmaceuticals, ibuprofen had the highest rejection of about 50%. This phenomenon may be due to the membrane’s pore size becoming smaller as ibuprofen was trapped by sorption to the membrane. 

In addition, the increase in organic concentration in the permeate correlated with the decrease in flow rate. This phenomenon was seen in the filtration test of TCP [178]. Performance changes were synchronized: flow rate and rejection reached a steady value after 2–3 h. As a result of examining the rejection characteristics of phenolics by NF membranes (NF90 and NF270), no decrease in the apparent rejection of phenol, resorcinol, and hydroquinone was observed for NF90 over a 3 h measurement, whereas the rejections of m-nitrophenol and m-chlorophenol decreased over the first two hours, indicating sorption to the membrane [565]. This behavior is consistent with the initial flow loss observed in phenolics [280]. Similar phenomena have been reported [566,567,568], with particularly notable changes observed with organic molecules slightly smaller than the MWCO of the RO/NF membranes [289,569].

### 10.2. Free Volume Reduction by LMWOC Sorption into RO/NF Membranes

**Figure 13:**
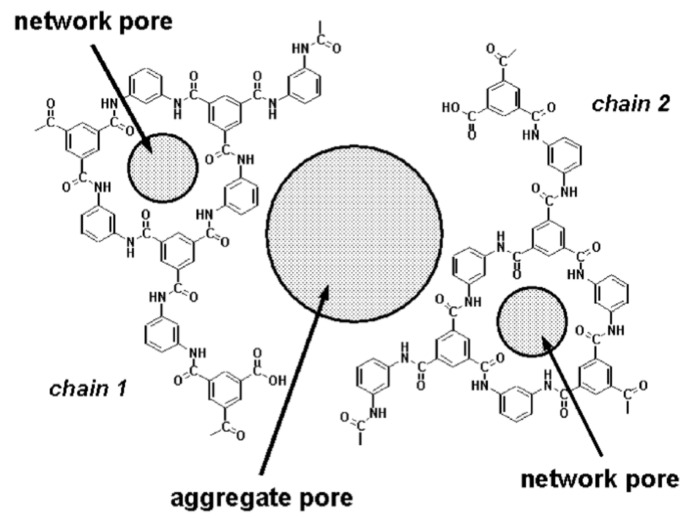
Schematic representation of possible molecular structure of network and aggregate pores in APA TFC membranes [572]. Reprinted with copyright permission.

Many researchers have tried to estimate the average pore size and pore size distribution in RO membranes (often called free volume). For example, Chan et al. [570] investigated the change in membrane pore size due to heat treatment of CA membranes. They reported that untreated membranes have two pore distributions with radii of 0.6–1.0 nm and 3.6–6.1 nm. They noted that heat treatment reduces the number of large pores and the size and diameter of small pores. Henmi et al. [571] reported a correlation between pore size determined by PALS and boron rejection. Kim et al. [572] reported a bi-modal pore size distribution of TFC RO (network and aggregate pores) determined by the PALS, as shown in Figure 13.

The respective sizes were reported to be 0.21–0.24 nm and 0.35–0.45 nm. Bi-modal distributions are also commonly seen in highly permeable glassy polymers, with small pore radii of 0.25–0.35 nm and large pore radii of 0.35–0.7 nm [573]. Consolati et al. [574] stated that aggregate pores originate from the non-equilibrium state of the glassy polymer. Boussu et al. [213] also reported a bi-modal distribution for various NF membranes. However, it should be noted that there exist papers describing the uni-modal pore size distribution rather than bi-modal [575]. Shimazu et al. [576] reported that τ4, the long lifetime of ortho-positronium (aggregate pores), was not detected for PA membranes exhibiting high salt rejections. One report shows that when a TFC RO membrane was immersed in methanol, the lifetime distribution changed from uni-modal to bi-modal due to swelling [577]. 

Nyström et al. [578] investigated fouling and retention of different NF membranes using different model substances. They concluded that some types of molecules passing the pores of the membranes can be entrapped and sorbed into the membrane. This phenomenon can either increase or decrease the flux depending on the effect on the free volume. Bartels and Franks [176] pointed out that blocking large macrocycles (aggregate pores) with LMWOC could significantly reduce the flow rate of the membrane and could equally reduce the passage of salts that “slip” through the membrane barrier layer. Saenz de Jubera et al. [579] proposed the modification mechanism of NF membranes with three aramid dendrimers (respective molar masses of 1016, 2192, and 4544 g/mol) that the dendrimers are trapped in aggregate pores. They speculate that the dye (rhodamine WT) rejection is improved by reducing the free volume of large pores. 

The same phenomenon has been observed in the gas permeation phenomenon of PMSP membranes. When DOP vapor was sorbed into the membrane, aggregate pores were reduced, and the network pores increased and shifted to smaller pore sizes, resulting in decreased gas permeability [505]. Kim et al. [572] reported that adding DMSO during interfacial polymerization increases the size and number of network pores and reduces the number of aggregate pores. This phenomenon might be attributed to the reduction in aggregate pores due to DMSO being trapped in aggregate pores. As explained, the residual solvents (NMP and DMSO) reduce gas permeability and act as antiplasticizers in PES [506,507]. The addition of antiplasticizers to polymers is known to hinder gas and vapor permeation by reducing the free volume [508]. García et al. [580] tested several additives with structural properties that cause antiplasticization to modify the transport properties of the amorphous PA Trogamid T™ and determined the size of the free volume pores by PALS. The results showed that 1,5-dihydroxynaphthalene, in particular, acted as an antiplasticizer, reducing the size of the network pores. Jackson and Caldwell [361] stated that antiplasticizers must be thin, polar, and rigid molecules compatible with the polymer. Thus, antiplasticizers and LMWOC foulants in RO play a similar role in reducing free volume. The difference between antiplasticizers and LMWOC foulants in RO/NF membranes is that antiplasticizers are added during the molding and membrane formation of polymers, reducing the free volume of the finished products. In contrast, in RO/NF membrane fouling, foulants are mainly trapped in the large free volume (aggregate pores) already present due to strong interactions with the membrane, reducing the overall average free volume.

The method to estimate the decrease in pore size with the progression of fouling requires measurement of the change in rejections of non-dissociated LMW compounds, e.g., urea, methanol, EG, NDMA, and boron. As already mentioned, boron has been shown to have a reasonable correlation with the free volume radius of the membrane. Several methods have been disclosed to improve boron rejection. For example, large iodine atoms are to be immobilized in the membrane by iodine post-treatment [581]. Hot water treatment also plays the same role in reducing the free volume and improving the boron rejection [582]. Fujioka et al. [583] reported that heat treatment of RO membranes at 80 °C for 4 h enhanced NDMA separation performance considerably. Relating to this, fouling with secondary sewage effluent can be reduced by heat treatment of the membrane [584]. Chen et al. [199] investigated the effect of surfactants on the properties of RO membranes and the removal of trace nuclides in low-level radioactive wastewater. The study investigated the effect of surfactant fouling (anionic, cationic, and nonionic surfactants) on the removal of trace nuclides and the membrane permeation flux. They reported that the boron rejection increases with a significant decrease in the initial flow rate by surfactants. As for other LMWOCs, Bellona et al. [494] found that methanol, urea, and ethanol rejections increased in membranes fouled with a secondary effluent. Analysis of the fractionation characteristics of LMWOCs reported that the pore size of the NF90 membrane decreased from 0.176 to 0.163 nm before and after fouling. Fujioka et al. [585] conducted fouling tests using three substances, alkylamines, amides, and epoxides, with N-nitrosamines as a proving substance for membrane permeation. They found that alkylamines noticeably reduced the permeation flow rate. As the carbon number of the alkyl group increased from 6 to 12, the flow rate decreased linearly, and the NDMA rejection improved from 55% to 80% (40% rejection for the pristine membrane). 

In a fouling test using tertiary effluent, a significant decrease in flow rate was observed in APA-NF (NF90) [496]. An increase in the rejections of N-nitrosamines was also observed for the fouled membrane. For ULP RO (ESPA2), the rejection of low MW NDMA was specifically increased. On the other hand, the dense membranes (ESPAB) did not show appreciable rejection improvements of N-nitrosamines after the membrane was fouled. ESPAB was developed as a membrane with high boron rejection, and it is assumed that LMWOC is difficult to sorb due to its small pore size. However, the radius of the network pores measured by the PALS method was not different between ESPA2 and ESPAB [586]. They did not mention the number or size of the aggregation pores, which may have affected the removal rate of N-nitrosamines and boron. In terms of boron rejection, from indirect measurements, i.e., the concentration of dissociated carboxyl group (R-COO^−^) at pH 6 and 10 and the temperature at which 20% weight loss occurs, Suzuki et al. [587] speculate that aggregate pores play an important role in boron permeation through RO/NF membranes. Further research is anticipated to elucidate the effect of the aggregate pores on removing LMWOCs and boron and its relation with fouling.

From these results above, it can be inferred that fouling of LMWOC occurs when the foulants are sorbed in the large free volume in the membrane (the aggregate pores and larger pores within network pores), which reduces the average free volume and the permeation flow rate. On the other hand, for hydrophilic substances such as linear PEG, it is speculated that increasing the molecular weight reduces their diffusivity through the membrane and causes them to become permanently trapped within the membrane, thereby restricting the flow channels and decreasing the flow rate.

### 10.3. Factors of LMWOC Governing Fouling

So far, it has been mentioned that fouling with LMWOCs is affected by sorption (surface and aggregate pores) and diffusivity in the membrane. It was also reported that sorption of LMWOCs to the membrane also occurs within the PSF porous support layer [131]. Therefore, the PSF support layer is thought to contribute to flux reduction since it is known that some organics reduce UF flux. However, there have been few studies on the contribution of PSF support. Thus, this area should be a topic for future research. The interaction between the membrane and the organic substance governs the sorption of LMWOCs to the membrane. From the diffusivity perspective, the molecules should have an appropriate size to pass through or be trapped in the membrane aggregate pores. These factors are further examined below.

Williams et al. [178] investigated phenolics and benzene fouling behavior. They found that the flux of non-ionized ortho-substituted phenols decreased according to the amount of adsorption. However, there was no correlation between the amount of adsorption and the flux reduction for di- and tri-substituted phenols (DNP, DCP, TCP). In addition, phenol had a low adsorption amount and only a slight flux reduction, whereas benzene showed only a slight decline despite its high physical adsorption. The flux reduction of phenolics showed a good correlation with pKa but not the amount of adsorption. Notably, this pKa correlation cannot apply to non-dissociable organics such as benzene.

Bruggen et al. [122] investigated the effects of organics’ physicochemical characteristics, such as dipole moment, the n-octanol-water partition coefficient (log P), and molecular size, on various organics adsorption and flux reduction of NF membranes. They found that the flux reduction occurs due to a combination of two effects: the molecules should have the appropriate size to fill the membrane pores, and the hydrophobicity of the components enhances the adsorption process.

Hydrophobic and electrostatic interactions are known to play essential roles in LMWOC sorption. Jiang et al. [588] studied the interactions between three membrane materials (Polysulfonamide, polybenzimidazolone, and APA) and about 90 organic solutes by the high-performance liquid chromatography method. Interactions between membrane materials and organic solutes strongly depend on the structure and chemical properties of polymers and organic solutes as follows:

- Increasing the number and hydrophobicity of the hydrophobic groups of organic solutes favors interactions between membrane materials and organic solutes;
- Introducing electron-releasing groups in the aromatic compounds favors interactions;
- Introducing electron-withdrawing groups in the aromatic compounds does not favor interaction;
- Aromatic compounds have stronger geometric matching interactions with membrane materials containing aromatic rings than the corresponding aliphatic compounds.

The hydrophobic interaction has been judged from the n-octanol/water partition coefficient [589,590]. A good correlation was found between the adsorbed mass and the logarithm of the octanol-water partition coefficient (log P) [121,122,591]. In addition, when only molecules of similar size were selected, a clear correlation was found between log P and normalized water flux [122]. Molecules with a positive log P have a larger influence on the water flux when the partition coefficient is larger. 

In order to evaluate the hydrophobicity of charged compounds such as phenolics, aniline, and benzoic acid, one idea is to use the logarithm of the pH-dependent n-octanol/water distribution coefficient (log D_ow_) instead of log P. Li et al. [281] evaluated RO membrane fouling with weak acidic and basic organic compounds and examined how to correlate the log D_ow_ with a decline in flux. A reasonable correlation between the initial fouling rate and log D_ow_ was found at pH 7.0, except for positively charged aniline and 4-nitroaniline. On the contrary, there is no good correlation between irreversible flux decline and log D_ow_, pH 7.0. It seems challenging to correlate membrane flux decline with log D_ow_ alone since membranes and organics are both charged, resulting in strong electrostatic interactions.

Regarding the effect of organics MW or molecular size, the effective diameter and molecular weight were found to affect the flow rate reduction. For NF70 and UTC-20, the flux reduction is correlated with the molecular size, and both membranes show a peak flux reduction at an effective diameter of about 0.5 nm, with the effect of NF70 being somewhat more rapid than that of UTC-20 [120]. The average flux reduction of NF70 is more significant and for molecules between 0.45 and 0.55 nm of effective diameter, the average flux reduction being 3.4% for UTC-20 but 11.0% for NF70. In terms of molecular weight, it can be seen that molecules with MWs of 90 to 120, which were most significantly adsorbed to the membrane surface, also have the most significant effect on the water flux [122]. The effect of DOC on the water flux can only be seen in a relatively narrow range of MWs, which is somewhat lower than the MWCO of the membrane. 

For NF membranes, the fractionation characteristics vary from membrane to membrane, and the size of the aggregate pores may also vary. For the LES90 NF membrane, the Chinese gallnut tannic acid with a MW of 1700 significantly affected performance changes [289], suggesting the presence of large aggregate pores. Since the MWCO of RO is usually 100 or less, it is speculated that smaller molecules with an effective diameter have a prominent effect compared with NF membranes. 

In the case of benzene (log P = 2.13) and n-hexane (log P = 4.11) [592], the flow rate reduction is low despite their large log P value. This phenomenon may be because the effective diameter is small, which results in a high diffusion coefficient and makes them less likely to be trapped by the membrane.

## 11. Conclusions

Fouling is a serious concern when treating marginal waters. In RO/NF, biofouling, organic and colloidal fouling, and scale formation are particularly problematic. Of these, organic fouling, along with biofouling, has been considered difficult to manage. Fouling by LMWOCs such as surfactants, phenolics, and plasticizers is known, but there have been few comprehensive reports. This review sheds light on fouling behavior by LMWOCs and its underlying mechanisms.

By reviewing the case studies and troubleshooting efforts, typical symptoms of LMWOC fouling were identified as follows:

- Sharp flow decline within a short time of contact;
- Salt rejection sometimes increased;
- No appreciable differential pressure increase;
- Organic concentration is very low, sometimes a few ppm or less;
- No visible foulant can be seen when fouled elements are autopsied;
- Flow loss is sometimes irreversible or difficult to restore through regular cleaning;
- AC pretreatment is effective in alleviating problems.

Next, relevant foulants were thoroughly reviewed, including surfactants, phenolics, tannic acid, dyestuffs, aromatic compounds, O&G, and leachables from plant components. The role of LMWOCs is also outlined for other polymeric membranes. The LMWOC fouling is not a specific case but ubiquitous in RO/NF treatment. The following are key findings:

- Electrostatic and hydrophobic interactions between membranes and organics play essential roles in fouling phenomena;
- Surfactants, especially nonionic surfactants, phthalates, and leachable from epoxy resins, are the foulants that require the most attention;
- In the case of nonionic surfactants, hydrophilic longer-chain ethoxylates contribute to fouling. Thus, prominent fouling is seen in PEG as well;
- Low MW aliphatic and aromatic hydrocarbons, e.g., n-hexan, benzene, etc., do not show noticeable flow decline at low concentrations. Thus, O&G may not be a good feedwater quality indicator;
- Leachables from plant components might be an issue for newly constructed plants;
- Similar fouling can be seen in other membrane processes, e.g., UF, gas separation membranes, etc.;
- NOM and EfOM are mixtures of various organics. Low MW portions contribute more initial fouling.

Regarding the fouling mechanism, internal fouling is more important than the external fouling. LMWOCs are trapped in aggregate pores. Trapped organics hinder water permeation, and as a result, salt rejections can be improved. Two factors are critical for the entrapment of organic compounds within membrane pores: molecule size (or MW) and membrane-organic interactions. The molecules should have the appropriate size to fill the membrane pores, and the hydrophobicity of the components enhances the sorption process. The effect of LMWCOs on the water flux can only be seen in a relatively narrow range of MWs, which is somewhat lower than the MWCO of the membrane.

Hydrophobic interaction can be judged by the n-octanol/water partition coefficients. A clear correlation was found between log P and normalized water flux. Molecules with a positive log P have more influence on the water flux when the partition coefficient is larger.

This review aims to provide necessary information on LMWOC fouling that has not been focused on before. The author hopes this review can be used as a one-stop reference database when readers want to know about their fouling problems, symptoms, mechanisms, corrective measures, etc.

## Figures and Tables

**Table 1:** Pressure-driven membrane processes and definition [1,39,41].

Membrane Process	Terminology for Membrane Processes	Pores	TMP
Microfiltration (MF)	Particles and dissolved macromolecules larger than 0.1 µm are rejected	0.1–5 µm	100–500 kPa
Ultrafiltration (UF)	Particles and dissolved macromolecules smaller than 0.1 µm and larger than about 2 nm are rejected	1–100 nm	100–800 kPa
Nanofiltration (NF)	Particles and dissolved molecules smaller than about 2 nm are rejected	0.5–10 nm	0.3–3 MPa
Reverse osmosis RO)	Applied transmembrane pressure causes selective movement of solvent against its osmotic pressure difference	<0.5 nm	1–10 MPa

**Table 2:** Key factors of membrane fouling.

Factor	Description
Locations	External surfaces, pore openings, or within pores
Mechanism	Adsorption, sorption, deposition, accumulation
Foulants	Suspended or dissolved substances (retained particles, colloids, macromolecules, salts, etc.)
Time course	Immediate or quick response, change with time
Issues or effects	Loss of performance (flux, product quality, membrane life)

**Table 3:** Typical membrane foulants.

Foulants	Description
Particulates	Suspended particles, flocs, yeast, pulp, silicon particles from wafer shaping processes [65], etc.
Colloids	Inorganic colloids: Silica, aluminosilicate, iron oxide, etc. Organic colloids: Transparent exopolymer particles (TEP)
Organics	Macromolecules: Humic and fulvic acids, polyphenols, Biopolymers, proteins, polysaccharides (pectin, cellulose, hemicellulose, and starch [66]), etc. LMWOCs: Antifoam agents such as propylene glycol [67], oil and grease, fats, fatty acids, etc.
Inorganics	Scaling, sparingly soluble salts: CaCO_3_, CaSO_4_, Ca_3_(PO_4_)_2_, etc., silica
Biological	Microorganisms, bacteria, EPS, etc.

**Table 4:** RO/NF foulants classification.

Types of Fouling		Surface	Internal	Foulant Examples
Particulate/colloidal		✓		Clay, Silt, Colloidal silica and sulfur, activated carbon fines, etc. Metal hydroxides/oxides: Al_3_+, Fe_3_+, Mn_2_+ Organic colloids: Particulate humic substances, colloidal TEP
Scaling (Sparingly soluble compounds)		✓		Carbonate: CaCO_3_ Sulfates: CaSO_4_, SrSO_4_, BaSO_4_ Phosphates: Ca_3_ (PO_4_) _2_ Metal hydroxides: γ-FeOOH, Al(OH)_3_ Others: CaF_2_, silica, alumino/iron silicates, FeS, calcium citrate, etc.
Biofouling		✓		Microorganisms, biofilms, EPS
Organic	Macromolecules	✓		NOM (biopolymers, HA, and fulvic acids), EfOM (soluble microbial products, SMP) Proteins, polysaccharides, polyphenols, Polyethylene glycol (PEG), etc. Organics added during pretreatment: Flocculants, antiscalant
	LMWOCs	✓	✓	SOCs: Nonionic surfactants, plasticizers, phenolic derivatives, quaternary ammonium biocides, leachables from anion exchange resin, etc. LMW portion of NOM and EfOM Others: Long chain fatty acids, oil and grease, etc.

**Table 5:** Summary of main fouling composition detected on membranes autopsies (%).

Feed Type	Organic/ Biological	Colloid/ Particulates	Scale	Metal	Organics	Others	Year	Reference
Overall (*n* = 150)	50.2	18.2	9.9	7.6	-	13.5	2001	[105]
Overall	23.9	38	5.4	3.3	29.3	-	2005	[106]
Overall	37.6	31.2	31.2 *^1^	-	-	-	2007	[107]
Seawater (*n* = 57)	47.4	17.5	14	21.1	-	-	2011	[108]
Overall (*n* = 500)	31.3	29	22.1	10	7.7 *^2^	-	2013	[69]
Seawater	40.5	38	1.3	20.3	15.2 *^2^	-	2013	[69]
Brackish Water	26.3	24.8	21.4	5.6	4.1 *^2^	-	2013	[69]
Seawater (*n* = 500)	54.1	23.9	4.8	6.2	-	-	2021	[72]
Wastewater (*n* = 50) *^3^	44.1	7.5	30	18.3	-	-	2022	[109]

*^1^: Scales and Inorganic deposits; *^2^: Mainly SOCs such as mineral oil, cellulose, chlorine compounds, polyacrylamide/polyacrylate, etc.; *^3^: 50 autopsies performed on MF, UF, NF, and RO.

**Table 6:** Classification of DOM contained in RO/NF feedwater.

	SF	GW	SW	IW	MW	Organics	MM	LM	Examples
**NOM**						Biopolymers	**✓**		Polysaccharides, protein
						Humic substances	**✓**		HA, fulvic acid
						Building blocks		**✓**	Hydrolysates of humic substances
						Organic acids		**✓**	Monoprotic acids, fatty acids, etc.
						LMW neutrals		**✓**	Alcohols, aldehydes, ketones, amino acids
**SOC**						Flocculants	**✓**		Polyacrylamides, cationic flocculants
						Antiscalant	**✓**	**✓**	Polyacrylates, phosphonates, etc.
						Fatty acids		**✓**	Higher fatty acids (octanoic, stearic acid, etc.)
						Oil and grease		**✓**	Long-chain hydrocarbons, BTEX, etc.
						Micropollutants		**✓**	Pesticides, PPCPs, PFAS, etc.
						Others		**✓**	Surfactants, plasticizers, dyes, phenolics, quaternary ammonium compounds, DBPs, etc.
**SMP** **AOM**						Biopolymers	**✓**		Polysaccharides, proteins
	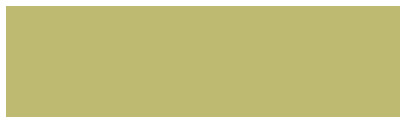		Key components				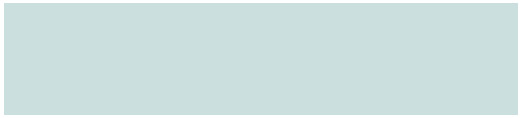		Minor components

SF: Surface water, GW: colored groundwater, SW: seawater (open intake), IW: industrial wastewater, MW: municipal wastewater. MM: macromolecules, LM: LMWOC, BTEX: Benzene, Toluene, Ethylbenzene, and Xylenes, PPCPs: pharmaceuticals and personal care products

**Table 9:** Effect of surfactants on various RO/NF membranes.

Material	CA	APA	APA	APA	PVA Derivative	Aromatic Polyurea	PPA	Sulfonated PES
Membrane RO/NF	SC-3000 RO	FT-30 RO	759HR RO	UTC-73 RO	729HF RO	719HF RO	NF270 NF	NTR7450 NF
Anionic	⚪ SO	⚪ SDS	⚪	⚪ SO	⚪	✕	⚪ SDBS	⚪ SDBS
Nonionic	⚪ TW20	✕ TX	✕	✕ TW20	△	⚪	⚪ Neo	✕ Neo, TX
Cationic	⚪ CTAB	✕ Hyamine	✕	✕ CTAB	⚪	⚪	✕ Cetrimide	✕ Cetrimide
Zwitterionic	–	–	✕	–	⚪	⚪	–	–
PEI	–	–	⚪	–	–	–	–	–
References	[181]	[38]	[195,196]	[181]	[195,196]	[195,196]	[197]	[197]

APA: Aromatic polyamide, PA: Polyamide, PVA: Polyvinyl alcohol, PES: Polyether sulfone, SO: Sodium oleate, SDS: Sodium dodecyl sulfate, SDBD: Sodium dodecyl benzenesulfonate, TW20: Polyoxyethylene (20) sorbitan monolaurate, TX: Triton X-100, Neo: Neodol, CTAB: Cetyltrimethylammonium Bromide, Hyamine 3500: Blend of alkyl dimethyl benzyl ammonium chlorides, PEI: Polyethyleneimine. Flux Decline: ⚪: ≤15%, △: >15% <30%, ✕: ≥30%.

**Table 10:** Effect of nonionic surfactants on various NF membranes.

Membrane	Material	Contact Angle (°)	Surfactant	Concentration (ppm)	Relative Flux (%)	Reference
NF40	PPA	–	Triton X-100	1% (immerse)	113	[192]
NF270	PPA	27	Fasavin CA 73	200	80	[210]
			Neodol	40	88	[197]
Desal 5DL	PA	44	Fasavin CA 73	200	111	[210]
Desal 51HL	PA	47	Neodol	40	86	[197]
UTC-20	PPA	36	Fasavin CA 73	200	167	[210]
XN-45	PPA	–	NPE	50	80	[211]
NTR-7450	Sulfonated PES	70	Fasavin CA 73	200	5.7	[210]
			Neodol	40	30	[197]
N30F	PES	88	Fasavin CA 73	200	1.5	[210]
			NPE	50	16	[211]
NF PES10	PES	72	NPE	50	34	[211]
			Neodol	40	55	[197]

Fasavin CA 73: A mixture of several nonionic surfactants and minor amounts of anionic surfactants. The molecular size of the monomers ranges from 742 up to 1072 Da. The hydrophobic carbon chain length ranges from 9 to 18 carbon atoms, while the size of the hydrophilic ethoxy group varies between 10 and 16. Neodol: RO(CH_2_CH_2_O)_n_H where R = C_9_ − C_11_ and n = 5. NPE: Nonylphenol ethoxylate, n = 9.

**Table 11:** Stabilized permeate flux at 1.2 MPa after adding surfactants, fatty acid, and PEGs.

Additives	Concentration (mg/L)	Stabilized Flow (m^3^/m^2^/day)
None	-	1.0
AE (C12+EO7)	0.1	0.80
AE (C12+EO7)	1	0.55
AE (C12+EO7)	10	0.23
AE (C12+EO20)	1	0.42
Faty acid (C12)	1	0.85
PEG (EO20)	1	0.67
PEG 106	1	0.9–0.95
PEG 106	10	0.9–0.95
PEG 1470	0.1	0.9
PEG 1470	1	0.55
PEG 7100	0.1	0.55
PEG 7100	1	0.3

**Table 12:** A list of surfactants used as model foulants during low-fouling membrane development.

Surface Modification	Cationic	Nonionic	Anionic	Reference
Polyvinyl alcohol (PVA)	✓			[229]
Cross-linked hydrophilic compounds		NCW-601A		[230]
Proprietary surface modification (neutrally charged)	✓	✓	✓	[231]
Cross-linked hydrophilic polymer		POE		[209]
Hydrophilic coating	DTAB			[232]
PEG Graft	DTAB			[233]
PVA coating	DTAB			[234]
A brush-like polymer containing PEG chains	DTAB			[235]
Polyelectrolytes layer-by-layer assembly	DTAB			[227]
Commercial low-fouling RO membranes	DTAB	Triton X		[236]
Proprietary hydrophilic monomers and cross-linking polymerization	DTAB	Triton X-100		[237]
Commercial low-fouling RO membranes		Triton X-100		[238]
Commercial low-fouling RO membranes	BAC	NCW-1002	SDS	[239]
Cross-linked PEG-based hydrogels	DTAB		SDS	[240]
PVA grafting	DTAB		SDS	[241]
Polyvinylamine grafting	DTAB			[242]
PVA—a pressurizing method	BAC			[228]
PEG derivatives	DTAB			[243]
PEG diglycidyl ether	DTAB		SDS	[244]
Cross-linked PEG	DTAB			[245]
PEI by self-assembly	DTAB			[246]
PEI-PEG dendrimer	DTAB			[247]
PEG-modified APA RO membrane	DTAB		SDS	[248]
In situ modification with amidosulfonic acid, diethanolamine, and piperazine	DTAB			[249]
Zwitterionic polymer coating	DTAB		SDS	[250]
Grafting of dialdehyde carboxymethyl cellulose	DTAB	POE	SDS	[251]
Fluorinated polyethyleneimine	DTAB			[252]

DTAB: dodecyl trimethylammonium bromide, SDS: sodium dodecyl sulfate, BAC: benzalkonium chloride, NCW-601A: polyoxyethylene alkyl phenyl ether, POE: polyoxyethylene octyl phenyl ether, NCW-1002: polyoxyalkylene alkyl ether.

**Table 13:** Surfactants containing process and wastewater in various industries.

Industry/Applications	Constituents Other Than Surfactants	References
Textile	Various dyes (Reactive, direct, etc.), waterglass, builders, etc.	[253,254,255,256]
Laundry	Oil-in-water emulsion, builders, fat, COD/TOC	[193,257,258]
Car wash	Oil-in-water emulsion, TDS, hardness, SS	[259,260]
CIP (Aqueous cleaners)	Oil-in-water emulsion, oil and grease, builders, additives, etc.	[261,262,263,264]
Oil and gas (Produced water)	Hydrocarbon, additives for enhanced oil recovery, etc.	[265,266]

**Table 14:** Summary of RO/NF research for treatment of phenolic compounds.

Evaluated Phenol Derivatives	Membranes	Reference
Phenol (PHE), Alkyl phenols (4-Methylphenol, 4-Ethylphenol, 2,6-Dimethylphenol, 4-n-Propylphenol, 4-Isopropylphenol)	FT-30	[131]
PHE, 2-Chlorophenol (2CP), 4-Chlorophenol (4CP), 2,4-Dichlorophenol (DCP), Chlorocresol, 2-Nitrophenol (2NP), 4-Nitrophenol (4NP), DNP, TCP	FT30-BW	[132]
2CP, DNP, TCP	NF40, FT30	[271]
TCP	NF40, ROM378, DRA4020	[272]
PHE, 2-Aminophenol (2AP), 2-Flourophenol (2FP), 2CP, DCP, TCP, 2NP, DNP	FT30-BW	[178]
PHE	NF70, UTC-20	[121]
Phloroglucinol, Resorcinol, 3- Hydroxybenzoic acid	NF70	[273]
PHE, Catechol, Resorcinol, Hydroquinone	TFC-HR, NF90	[274]
PHE, Catechol, Resorcinol, Hydroquinone, Pyrogallol, Phloroglucinol, 2CP, 3-Clorophenol (3CP), 4CP, 2NP, 3-Nitrophenol (3NP), 4NP	NF90	[275]
PHE, Catechol, Resorcinol, Hydroquinone, Pyrogallol, Phloroglucinol, 2CP, 3CP, 4CP, 2NP, 3NP, 4NP	TFC-HR, BW30, NF90	[276]
2NP, 2CP	NF90, BW30	[277]
BPA	NFD, NF90, NF270, ESNA1-LF2, CK	[278]
4NP	RO90	[279]
2CP	TW30	[280]
PHE, Hydroquinone, 2CP, 4CP, 2NP, 3NP, 4NP, DNP	CPA2	[281]
PHE, 2CP and 2NP	CPA2	[282]

**Table 15:** pKa of phenolics.

Phenolic Compounds	Phenol (PHE)	4-Nitrophenol (4NP)	2,4,6-Trichlorophenol (TCP)	2,4-Dinitrophenol (DNP)
pKa	9.99	7.15	6.23	4.09

**Table 17:** APA RO and NF membranes.

Membrane Type	Typical Flow (m^3^/d)	Membrane Area (ft^2^)	Testing Conditions
LP RO	44–48	440	225 psi (1.55 MPa), 2000 ppm NaCl
ULP RO	44–50	440	150 psi (1.0 MPa), 2000 NaCl
Super ULP (SULP) RO	46–50	440	100 psi (0.7 MPa), 500 ppm NaCl
APA NF	30–45	400	70–75 psi (0.48 MPa)

**Table 19:** Comparison of fouling symptoms by LMWOCs and surface fouling.

Fouling by Certain LMWOCs	Surface Fouling (NOM, Colloids, Polysaccharides, Proteins, etc.)
Flow rate drops sharply after a short time of contact	Generally, fouling progresses slowly
Salt, boron, and LMWOC rejections increase.	Rejection improvements of LMWOC are not observed.
Foulant concentration is low, i.e., <a few ppm	High concentration causes rapid fouling symptoms
No visible foulants are found, which are difficult to detect by standard surface analysis.	Detectable by FT-IR, ESCA, SEM, etc.
Flow loss is sometimes irreversible and difficult to clean	Cleaning generally restores performance

## Data Availability

The author confirms that the data supporting the findings of this study are available within the articles, conference proceedings, patents, etc., listed in References.

## Abbreviations

2B3T	Two-bed, three-tower pure water system
2CP	2-Chlorophenol
2NP	2-Nitrophenol
4CP	4-Chlorophenol
4NP	4-Nitrophenol
AC	Activated carbon
AE	Alkyl ethoxylate
AEO	Alcohol ethoxylate
AOM	Algal organic matter
AOP	Advanced oxidation process
APA	Aromatic polyamide
APEO	Alkylphenol ethoxylate
ATD	Anti-telescoping device
ATP	Adenosine triphosphate
ATR	Attenuated total reflection
BAC	Benzalkonium chloride
BPA	Bisphenol A
BSA	Bovine serum albumin
BTEX	Benzene, Toluene, Ethylbenzene, and Xylenes
BWRO	Brackish water reverse osmosis
CA	Cellulose acetate
CAPB	Cocamidopropyl betaine
CEB	Chemical-enhanced backwash
CF	Cartridge filter
CIP	Cleaning in place
CMC	Critical micelle concentration
COD	Chemical oxygen demand
CTA	Cellulose triacetate
CTAB	Cetyltrimethylammonium bromide
CTBD	Cooling tower blowdown
DBP	Disinfection by-product
DCHP	Dicyclohexyl phthalate
DCP	2,4-Dichlorophenol
DEHP	Di-2-ethylhexyl phthalate
DEP	Diethyl phthalate
DMAc	N,N-Dimethylacetamide
DMF	N,N-Dimethylformamide
DMP	Dimethyl phthalate
DMSO	Dimethyl sulfoxide
DnBP	Dibutyl phthalate
DNP	2, 4-Dinitrophenol
DO	Dissolved oxygen
DOC	Dissolved organic carbon
DOM	Dissolved organic matter
DOP	Dioctyl phthalate
DP	Differential pressure
DTAB	Dodecyltrimethylammonium bromide
ED	Electrodialysis
EDTA	Ethylenediaminetetraacetic acid
EfOM	Effluent organic matter
EG	Ethylene glycol
EPS	Extracellular polymeric substances
ESCA	Electron spectroscopy for chemical analysis
FPD	Flat panel display
FRP	Fiber-reinforced plastic
FT-IR	Fourier transform infrared spectroscopy
GAC	Granular activated carbon
GC	Gas chromatography
gfd	Gallons/ft^2^/day
GWRS	Groundwater Replenishment System
HA	Humic acid
HEM	n-Hexane Extractable Material
HLB	Hydrophilic-Lipophilic Balance
HPM	High-pressure membrane
HPO	Hydrophobic
ICP	Inductively coupled plasma
ICR	Information Collection Rule
IEX	Ion exchange
IR	Infrared
LAS	Linear alkyl benzene sulfonate
LBL	Layer-by-layer
LC-OCD	Liquid chromatography—organic carbon detection
LES	Lauryl ether sulfate
LMH	L/m^2^/h
LMW	Low molecular weight
LMWOCs	Low molecular weight organic compounds
Log Dow	Logarithm of the pH-dependent n-octanol/water distribution coefficient
Log P	Logarithm of the octanol-water partition coefficient
LOI	Loss on ignition
LP	Low pressure
LPM	Low-pressure membrane
MBR	Membrane bioreactor
MF	Microfiltration
MFI	Modified Fouling Index
MPD	m-phenylenediamine
MS	Membrane softening
MTCw	Water mass transfer coefficients
MWCO	Molecular weight cut-off
NDMA	N-nitrosodimethylamine
NF	Nanofiltration
NMP	N-methyl-2-pyrrolidinone
NOM	Natural organic matter
NP	Nitrophenol
OCWD	Orange County Water District
O&G	Oil and grease
O&M	Operation and maintenance
PA	Polyamide
PAC	Powdered activated carbon
PALS	Positron annihilation lifetime spectroscopy
PAN	Polyacrylonitrile
PEG	Polyethylene glycol
PEI	Polyethyleneimine
PEO	Polyethylene oxide
PES	Polyether sulfone
PFAS	Perfluoroalkyl substances
PFOS	Perfluorooctane sulfonate
PG	Propylene glycol
PHE	Phenol
pKa	Acid dissociation constant
PMSP	Poly(1-(trimethylsilyl)-1-propyne)
POE	Polyoxyethylene octyl phenyl ether
polyDADMAC	Polydiallyldimethylammonium chloride
PPA	Piperazine polyamide
PPCPs	Pharmaceuticals and personal care products
PPO	Polyphenylene oxide
PSF	Polysulfone
PT-A	Polyvinyl methyl ether
PT-B	Tannic acid
PV	Pervaporation
PVDF	Polyvinylidene fluoride
RBSMT	Rapid bench-scale membrane test
RO	Reverse osmosis
SDBD	Sodium dodecyl benzenesulfonate
SDGs	Sustainable development goals
SDI	Silt density index
SDS	Sodium dodecyl sulfate
SEM	Scanning electron microscopy
SMPs	Soluble microbial products
SO	Sodium oleate
SOC	Synthetic organic compound
SULP	Super ultra-low pressure
SWRO	Seawater reverse osmosis
TBP	Tributyl phosphate
TCP	2,4,6-trichlorophenol
TDS	Total dissolved solids
TEP	Transparent exopolymer particles
TFC	Thin-film composite
TFN	Thin-film nanocomposite
TGA	Thermo gravimetric analysis
TMC	Trimesoyl chloride
TMP	Transmembrane pressure
TOC	Total organic carbon
TPI	Transphilic
UF	Ultrafiltration
ULP	Ultra low-pressure
UPW	Ultra-pure water
USEPA	US Environmental Protection Agency
XAD	Polymeric adsorbent resin
WBMWD	West Basin Municipal Water District
WF-21	Water Factory 21
WOCS	Weathered oil-contaminated seawater
